# Cancer Biomarker Discovery: The Entropic Hallmark

**DOI:** 10.1371/journal.pone.0012262

**Published:** 2010-08-18

**Authors:** Regina Berretta, Pablo Moscato

**Affiliations:** 1 Centre for Bioinformatics, Biomarker Discovery and Information-Based Medicine, The University of Newcastle, Callaghan, New South Wales, Australia; 2 Information Based Medicine Program, Hunter Medical Research Institute, John Hunter Hospital, New Lambton Heights, New South Wales, Australia; 3 Australian Research Council Centre of Excellence in Bioinformatics, Callaghan, New South Wales, Australia; Queen Elizabeth Hospital, Hong Kong

## Abstract

**Background:**

It is a commonly accepted belief that cancer cells modify their transcriptional state during the progression of the disease. We propose that the progression of cancer cells towards malignant phenotypes can be efficiently tracked using *high-throughput technologies* that follow the gradual changes observed in the gene expression profiles by employing Shannon's mathematical theory of communication. Methods based on Information Theory can then quantify the divergence of cancer cells' transcriptional profiles from those of normally appearing cells of the originating tissues. The relevance of the proposed methods can be evaluated using microarray datasets available in the public domain but the method is in principle applicable to other high-throughput methods.

**Methodology/Principal Findings:**

Using melanoma and prostate cancer datasets we illustrate how it is possible to employ Shannon Entropy and the Jensen-Shannon divergence to trace the transcriptional changes progression of the disease. We establish how the variations of these two measures correlate with established biomarkers of cancer progression. The Information Theory measures allow us to identify novel biomarkers for both progressive and relatively more sudden transcriptional changes leading to malignant phenotypes. At the same time, the methodology was able to validate a large number of genes and processes that seem to be implicated in the progression of melanoma and prostate cancer.

**Conclusions/Significance:**

We thus present a quantitative guiding rule, a new unifying hallmark of cancer: the cancer cell's transcriptome changes lead to measurable observed transitions of *Normalized Shannon Entropy* values (as measured by high-througput technologies). At the same time, tumor cells increment their divergence from the normal tissue profile increasing their disorder via creation of states that we might not directly measure. This unifying hallmark allows, via the the *Jensen-Shannon divergence*, to identify the arrow of time of the processes from the gene expression profiles, and helps to map the phenotypical and molecular hallmarks of specific cancer subtypes. The deep mathematical basis of the approach allows us to suggest that this principle is, hopefully, of general applicability for other diseases.

## Introduction

In a seminal review paper published nine years ago, Hanahan and Weinberg [1] introduced the “*hallmarks of cancer*”. They are six essential alterations of cell physiology that generally occur in cancer cells independently of the originating tissue type. They listed: “*self-sufficiency in growth signals, insensitivity to growth-inhibitory signals, evasion of the normal programmed-cell mechanisms (apoptosis), limitless replicative potential, sustained angiogenesis, and finally, tissue invasion and metastasis*”. More recently, several researchers have advocated including “*stemness*” as the seventh hallmark of cancer cells. This conclusion has been reached from the outcomes of the analysis of *high-throughput* gene expression datasets [2], [3]. The new role of stemness as a hallmark change of cancer cells is also supported by the observation that histologically poorly differentiated tumors show transcriptional profiles on which there is an overexpression of genes normally enriched in embryonic stem cells. For example, in breast cancer the activation targets of the pluripotency markers like NANOG, OCT4, SOX2 and c-MYC have been shown to be overexpressed in poorly differentiated tumors in marked contrast with their expression in well-differentiated tumors [4].

Other authors suggest different hallmarks, with many papers pointing alternative processes as their primary focus of their research. The difference may stem from the fact that these authors prefer to cite as “*key hallmarks*” physiological changes which occur at a “lower level” scale closer to the molecular events. These authors cite, for example, “*mitochondrial dysfunction*” [5], [6] (including, but not limited to “*glucose avidity*” [7] and “*a shift in glucosemetabolism from oxidative phosphorylation to glycolysis*” [6], [8], “*altered glycolysis*” [9], “*altered bioenergetic function of mitochondria*” [10]), “*dysregulation of cell cycle and defective genome-integrity checkpoints*” [11], “*aberrant DNA methylation*” [12] (“*promoter hypermethylation of hallmark cancer genes*” [13] and “*CpG island hypermethylation and global genomic hypomethylation*” [14]), “*shift in cellular metabolism*” [15], [16], [17], “*regional hypoxia*” [18], “*microenviroment acidosis*” [19], “*abnormal microRNA regulation*” [20], [21], “*aneuploidy*” and “*chromosome aberrations*” [22], [23], [24], [25], [26], “*disruption of cellular junctions*” [27], “*avoidance of the immune response*” [28], “*pre-existing chronic inflammatory conditions*” [29], [30], “*cancer-related inflammation*” [29], “*disabled autophagy*” [28], “*impaired cellular senescence*” [31], “*altered NF-kappaB signalling*” [32], “*altered growth patterns, not altered growth per se*” [33], “*disregulated DNA methylation and histone modifications*” [34], “*tissue dedifferentiation*” [35], [36], and “*somatically heritable molecular alterations*” [37]. This research enriches the list of the most important cancer hallmarks. However, these physiological changes occur at a “lower” molecular level they are likely related sub events of the orginial seven instead of newly discovered “key hallmarks”. More recently, Luo et al attempted a “stress-based” description of some of the hallmarks in terms of “stresses” (“*DNA damage/replication stress, proteotoxic stress, mitotic stress, metabolic stress, and oxidative stress*”) [38]. While this is an interesting descriptive grouping, it is still a phenotypical characterization. What is needed is a higher level unifying genotypical characterization, from which individual disregulated processes can be identified in a quantitative way using the existing high-throughput data capture methodologies. It is clear that a unifying hallmark is needed if we aim at quantifying the cell's progression. It is then evident for us that a unifying mathematical formalism is necessary to uncover the cell transcriptome's progression from a normal to a more malignant phenotype.

We start our quest assuming an implicit working hypothesis common to many research groups around the world: *the macroscopic physiological changes (i.e. Hanahan and Weinberg's “hallmarks”) must also correlate with global alterations of the molecular profiles of gene transcription*. It is also assumed that the “*hallmark changes*” occur along a certain timeline, but that some of the sub-processes discussed before are concurrent. These processes may start in a slow incremental way with some of the major changes being early events while others (e.g. tissue invasion and metastasis) are likely later processes triggered by new events during cancer progression. The timeline is not explicit and it is also likely that cancer subtypes progress to similar timelines. In some cases the sequence of events are better understood (e.g. some leukaemia subtypes [39]). The elicitation and regulation of molecular events is likely to be an ongoing quest during this century for many types of cancer.

It is not to be assumed that some of the transitions of the transcriptome are gradual. That is a hypothesis that is unnecessary in this study. We envision that the progression of cancer may have “*switches*”, with a number of concurrent converging events leading to macroscopic observable changes in the gene expression profile resulting in dramatic variations of expression patterns. For instance, these molecular switches could not be characterized by an “oncogene” but by a large number of the genes that have changed its transcriptional state. These abrupt changes may be triggered by the confluence of several non-linear interactions, and are likely to be related to the physiological hallmarks we refer to above.

The presence of macroscopic observable changes that are computable from a large number of relatively smaller changes mean that *it may be possible to find an objective mathematical formalism to infer the turning point at which these radical changes occur*.

It is then evident that computing the *Jensen-Shannon divergences*, the *Normalized Shannon Entropy*, and the *Statistical Complexity* of samples reveal different global transcriptional changes. It is, however, not easy to infer if these changes would correlate with a gradual progression or sudden changes. However, one valid mathematical possibility is that the most important “*hallmark of cancer*”, a unifying principle above all, is the existence of a measurable gradual “progression” from a well-differentiated gene expression profile (corresponding to a healthy tissue). This would reveal the timeline of a higher level process that is observable and measurable via a change of *Normalized Shannon Entropy* and an increment of *Jensen-Shannon divergences* from the originating tissue type. If this is the case, by correlating the changes in *Information Theory* quantifiers with the expression of the genes we would be able to not only uncover useful biomarkers to track this progression but to explain the “*hallmarks*” in an ordered timeline. The timeline also yields clinical and translational important outcomes. Such analytical methodology will naturally produce “*a continuous staging*” of the cancer samples, based on a solid foundations of *Information Theory*, based on the knowledge of transcriptional profile of healthy cells as reference to measure divergences. In addition, as a mathematical methodology, it can be applied to other *high-throughput technologies* for which a probability distribution function of observed abundances has been computed.

With these ideas in mind, we provide a “transcriptomic-driven” method revealing important biomarkers for cancer progression a direction of time for which they are presented. The method, however, is generalizable to other type of *high-throughtput techonologies* (e.g. proteomic studies). We have chosen two types of cancers to study which are almost at the antipodes in terms of progression rates: *prostate cancer* and *melanoma*.

Prostate cancer progresses very slowly. Pathological samples are common in autopsies of men as young as 20 years old. By the age of 70 more than 80% of men have these alterations, a fact that already shows a relationship of this cancer type with increasing age. The clinical management of prostate cancer requires the identification of the so-called *Gleason patterns* in the biopsies [40], which after almost fifty years is still “*the sole prostatic carcinoma grading system recommended by the World Health Organization*”. However, undergrading, underdiagnosis, interobserver reproducibility and variable trends in grading have been observed as major problems [41], [42]. Melanoma, on the other hand, differs from prostate cancer in its rapid progression [43] and it is considered one of the most aggressive types of cancer. One of melanoma's usual markers of progression and concern (i.e thickness) is measured in millimetres, which gives a rough idea of how devastatingly fast the disease can spread.

We will present our results starting with one prostate cancer dataset, followed by another in melanoma, to come back to the prostate cancer discussion using another highly relevant dataset. This is a departure from the alternative approach in which each disease is discussed in separate sections. However, after considering several possibilities, we are convinced that our approach is the most appropriate to showcase the technique and its power. Details on the datasets and methods used are given in the ‘Materials and Methods’ section of this paper. We also refer to the original studies and manuscripts associated to the three datasets we analysed.

## Results

### Prostate Cancer – Lapointe et al.'s dataset (File S1)

**Figure 1:**
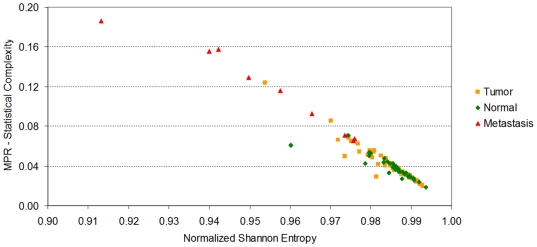
The *Normalized Shannon Entropy* and the *MPR-Statistical Complexity* for each of the 112 samples in Lapointe et al. [**44**]. Metastatic samples have typically lower values of *Normalized Shannon Entropy* than normal samples and prostate cancer primary tumors. The reduction in *Normalized Shannon Entropy* indicates that there exists a significant reduction on the expression of a large number of genes, or that the gene profile of metastatic samples has a more “peaked” distribution (due to the upregulation of a selected subset of genes). Both possibilities just cited are not mutually exclusive. We also note that neither the *Normalized Shannon Entropy*, nor the *MPR-Statistical Complexity* (as a single unsupervised quantifier), can help differentiate between tumor and normal samples, indicating that other Information Theory quantifiers are required for this discrimination.

The first dataset is the one from Figure one in Lapointe et al. [44]. This data is available from http://microarray-pubs.stanford.edu/prostateCA/images/fig1data.txt and supplemen-tary material is also available from http://microarray-pubs.stanford.edu/prostateCA/.

In the original study, the authors used a cDNA microarray technology that allowed them to measure gene expression of several thousand genes on 112 samples, including 41 normal prostate specimens, 62 primary prostate tumours and 9 lymph node metastases. From that set, a subset of 5,153 probes were selected as differentiating prostate cancer samples from normal and metastases (this is the set from figure one in Lapointe et al. [44] and available at the web address given above). After imputation of missing values, we first calculated the *Normalized Shannon Entropy* and the *MPR-Statistical Complexity* for the each sample.

The flowing section explains the context in which our results were generated (refer to the ‘Materials and Methods’ section for detail on how our quantities are computed). The *Normalized Shannon Entropy* measure is widely used in ecosystem modelling to quantify species diversity, where it is acknowledge as having great sensitivity to relative abundances of species in an ecosystem [45]. We utilise the same sensitivity to differentiate a samples in cancer datasets. Figure 1 shows that the *Normalized Shannon Entropy* of prostate cancer tumor samples do not differ much from normal samples. This is in contrast to lymph node metastasis samples that appear to have smaller values of *Normalized Shannon Entropy*.

A mathematical interpretation of this result is that the samples from lymph node metastases have cells that not only varied their transcriptomic profile, they have also “peaked” the distribution of expression values with significant fold increases on a smaller number of probes. This explains the reduction in *Normalized Shannon Entropy*. We note that there are several mechanisms that can explain a macroscopically observable global reduction of transcription. For instance, this may indicate that a relatively large number of genes have reduced their expression levels by genome damage, changes in gene regulation, or other silencing processes. It is reassuring to observe that the changes of the most prototypical quantitative measure we can draw from *Information Theory*, the *Normalized Shannon Entropy* correlate well with the transition between normal samples with to ones with metastases. However, it is also evident from that normal samples do not differentiate much from the tumor group (the *Normalized Shannon Entropy* values do not differ much). It is then not the number of genes with high expression values, but the change in the distribution of expression levels on the molecular profile, that can provide the other measure that could distinguish these other samples. This must be handled by the other statistical complexity measures to be discussed next.

Several statistical complexity measures can be defined which aim to clarify our argument. We will first discuss the results of computing the *MPR-Statistical Complexity* measure (in the previous figure the *y*-coordinates correspond to the *MPR-Statistical Complexity* values of each sample). The *MPR-Statistical Complexity* is proportional to both the *Normalized Shannon Entropy* associated to the transcription profile and the *Jensen-Shannon's divergence* between that probability density function and the uniform probability distribution. Again, we refer the reader to the ‘Materials and Methods’ section for an explanation of how these magnitudes are computed.

Although the results of using the *MPR-Statistical Complexity* might not seem particularly impressive, there are a few reasons why we introduce them at this stage. We want to illustrate a fact that can already be observed when we employ this measure on this dataset. In this dataset, for a given entropy value interval, normal tissue samples tend to have relatively lower *MPR-Statistical Complexity* values than tumor and lymph node metastasis. This means that both prostate cancer and metastases samples diverge from a “more uniform” distribution indicating that the distribution “peaks” in fewer active genes. It also means that, in terms of *Jensen-Shannon's divergence*, the transcriptional profile of a normal prostate cell sample is “*closer*” to a uniform distribution than to the one that is observed in a prostate cancer cell sample.

The reader will readily argue, and with reason, that the transcriptional profile of a normal cell is tissue-specific and that it hardly resembles that of a uniform distribution of expression values. That is correct and this observation motivates the introduction of two new statistical complexity measures. We generically call these two variants as ‘*M-complexities*’ (with ‘*M*’ standing for “*modified*”). They have the same functional form as the *MPR-Statistical Complexity*, but instead of computing the Jensen-Shannon's divergence from a uniform probability distribution we compute it against an ad hoc probability distribution functions derived from the data. In this sense, these measures are more supervised then the *MPR-Statistical Complexity* is. Another perspective is that the *MPR-Statistical Complexity* is a special case of this measure in which the ad hoc probability distribution function of reference is the equiprobability distribution. The relevance of this measure derives from being a general definition that allows accommodating several different reference states. We will use it to measure divergences to the “initial” and “final” transcriptomic states (two states of reference). Taken as computed averages over normal samples, and respectively metastatic ones, these measures will allow tracking the processes of differentiation of a cancer cell from a particular tissue type.

For example, using Lapointe et al.'s dataset, the *M-Normal* statistical complexity quantifier first requires the computation of the probability distribution function of the average gene expression profile of all normal prostate samples. Afterwards, the *Normalized Shannon Entropy* and the *Jensen-Shannon's divergence* of any sample profile will be computed using the divergence to that averaged normal distribution. Analogously, we compute the *M-Metastases* statistical complexity quantifier by first calculating the average profile of the metastases samples, and then generating the corresponding probability distribution function, finally computing the *Jensen-Shannon's divergence* with that profile. We refer to the ‘Materials and Methods’ section for details of the calculations.

**Figure 2:**
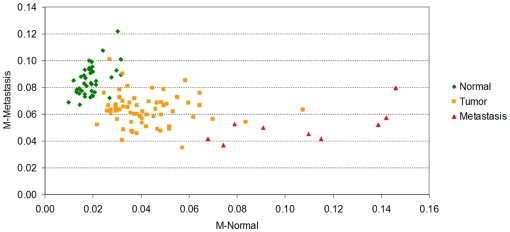
M-Normal against M-Metastases for the samples in Lapointe et al. [**44**]. We have seen in Figure 1, that the *Normalized Shannon Entropy* and the *MPR-Statistical Complexity* differentiate the metastatic samples from the normal samples, but that these two measures can not help to discriminate the primary tumors from the normals. We show here the results of two statistical complexity measures which are in some sense supervised (i.e. dependent on the dataset being interrogated). We call these two stastical mesured *M-Normal* and *M-Metastases*. They have the same functional form of the *MPR-Statistical Complexity*, but they use the average normal and average metastatic profile as probability distribution functions of reference. As a consequence, the *M-normal* and *M-metastases* are directly proportional to the *Jensen-Shannon divergences* with the normal (and respectively with the metastatic) gene expression profile. It is remarkable that, although we are using these end processes only (from Lapointe et al's, dataset of 5,153 probes×112 samples), most of the primary tumor samples appear as a transitional state between the normal and metastatic group. This is remarkable since the primary tumor samples were not used to define the *M-normal* and *M-metastases* measures and, in principle, the samples could have been located anywhere in the (*M-normal*, *M-metastases*)-plane. Computation of correlations of the probe expressions values can help us identify genes which are highly correlated with a divergence from the normal expression profile and, at the same time, converge towards the average metastatic profile.

The results can be observed in Figure 2. On the *x*-axis, the lymph node metastases have the largest values of *M-Normal* indicating a divergence from the normal profile. In addition, the *M-metastases* values of normal samples tend to be higher than most of the metastasis samples (with the exception of only one).

Figure 2 shows a gradual progression of the samples positions on this plane from a well-differentiated tissue type specific profile, first to a more heterogeneous primary tumor cluster, and finally to an even less differentiated metastatic profile.

The result presented in Figure 2 shows that the prostate cancer samples, which are not metastases and therefore could have been scattered anywhere on the plane, are clustered on a particular confined area between the two other groups. We understand that there are reasons to be sceptical about this result being not just a simple consequence of the gene selection process used by Lapointe et al. For example, *if we assume that* the 5,153 probes singled out by Lapointe et al. in their figure one of Ref. [44] (and that constitute our original data) have been selected with a supervised method that try to distinguish between normal and metastases, *then* the relative position of normal and metastases samples is perhaps something to be expected. However, even under that assumption, what is not expected is the position of all primary tumor prostate cancer samples, linking the normal cluster of samples with the metastases one. Note that the definition of both the *M-Normal* and *M-Metastases* measures do not use any information from the primary tumor prostate cancer samples, so the location of these samples between the normal cluster and the metastases, bridging them naturally is something to highlight. Together with Figure 1, it gives evidence that supports the working hypothesis that a gradual “progression” occurs, from the normal tissue specific profile to the metastasis one.

Indeed, following our line of argument, Figure 2 has even more relevance when we highlight the fact that the 5,153 probes *have not been selected with a supervised method*. The authors say that the only selection criteria was to single out the 5,153 cDNAs whose expression varied most across samples. In the supplementary notes of their paper the authors say: “*We included for subsequent analysis only well measured genes whose expression varied, as determined by (1) signal intensity over background >1.5-fold in both test and reference channels in at least 75% of samples, and (2) 3-fold ratio variation from the mean in at least two samples; 5,153 genes met these criteria.*” As a consequence, Figure 2 has been generated without class selection bias only using the genes that have the most varied expression pattern.

We now turn to another aspect of the statistical complexity and entropy analysis. We note that Figure 2 shows that the metastases samples have a clear reduction on *Normalized Shannon Entropy* in comparison with the values observed for the normal samples. At the same time, metastases samples, as expected, have higher *M-normal* complexity than the normal samples (Figure 2). It is then interesting to evaluate the value of the *Jensen-Shannon divergence* of these samples and to identify the genes that most correlate with the variations of *Jensen-Shannon divergence* to quantify one of the factors that is related to the statistical complexity changes.

**Figure 3:**
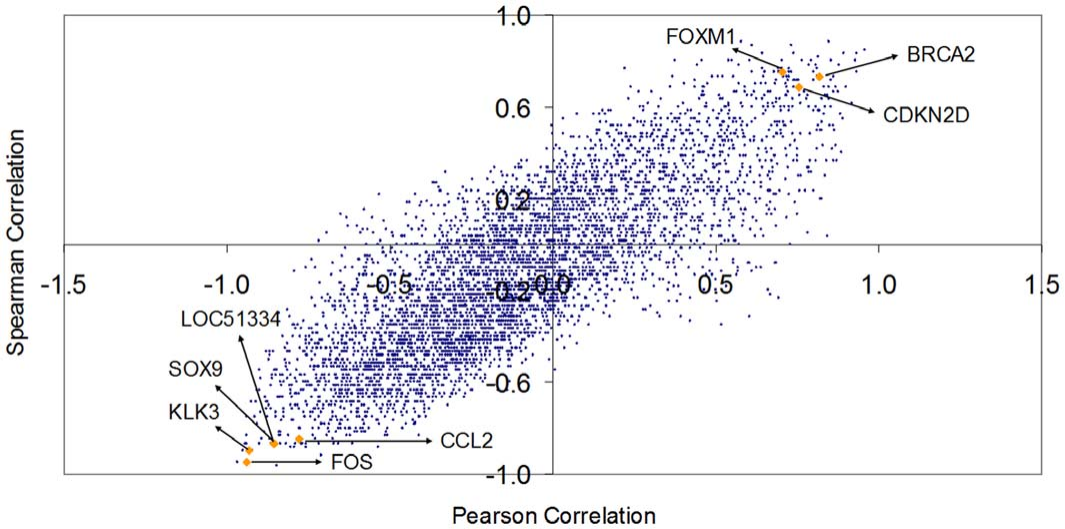
A scatter plot of each of the 5,123 probes of the dataset contributed by *Lapointe* et al. We have computed the Pearson and Spearman correlation of each probe expression (across samples) with the *Jensen-Shannon divergence* of each of the samples with the average metastasis profile (these values are called *JSM2-Pearson* and *JSM2-Spearman* in the accompanying Excel file provided). One of the clinically most relevant markers for prostate cancer (KLK3/PSA) together with FOS, CCL2/MCP-1, SOX9 and a probe for LOC51334 (mesenchymal stem cell protein DSC54) appear with highly negative Spearman and Pearson correlations values, indicating that they are negatively correlated with the *Jensen-Shannon divergence* from the average metastatic profile. BRCA2 (highly regarded as a tumor suppressor in cancer research), FOXM1 (a putative regulator of the mitotic program and the control of chromosomal stability [49]), and CDKN2D (a CDK4 inhibitor) in opposition with KLK3/PSA, seems to be positively correlated. As will be seen later in the analysis of the melanoma dataset, these positive correlations with the *Jensen-Shannon divergence* from the average metastatic profile indicate a possible dysregulation of these critical processes for which these genes have key roles.

We have computed the correlation of the gene expression profile corresponding to each of the 5,123 probes. For each of the 5,123 probes, we computed both the *Pearson correlation* (*x*-axis of Figure 3) and the *Spearman correlation* (*y*-axis of Figure 3) of each probe profile with the *Jensen-Shannon divergence* having as probability distribution of reference that of a metastasis profile (these values are called *JSM2-Pearson* and *JSM2-Spearman* in the accompanying Excel file provided). With this data, we have produced Figure 3, a scatter plot of the values associated to each probe. In this figure, there are two probes that are immediately recognizable by any cancer researcher, and in particular for those in prostate cancer: KLK3/PSA (Prostate Specific Antigen) and FOS.

The interpretation of these scatter plots is not immediate and needs an introductory explanation. Each dot corresponds to one probe of the array. For example, a dot that is very close to the origin of coordinates (0,0) indicates a probe such that its pattern of gene expression (across all samples) is not correlated with the *Jensen-Shannon divergence* to the average profile of a metastasis pattern. It is, in essence, a probe which is highly uninteresting in this regard. Probes that have a high correlation, across all samples, either positive or negative with the *Jensen-Shannon divergence* to the average profile of a metastasis pattern are highly informative. They “co-express” with this measure.

Although we provide in the supplementary material the information corresponding to all probes, we will discuss just a few of them. This will allow the reader to understand these plots and will put our results in the perspective with current research in prostate cancer. We particularly highlight the position of KLK3/PSA, FOS and CCL2. To our surprise, we have found which is perhaps the most famous biomarker in prostate cancer KLK3/PSA (Kallikrein-related peptidase 3), probe G_914588 (correlations of −0.9312 and −0.9000 respectively). FOS and KLK3/PSA are the second and the fourth most negatively correlated probes in this ranking of all the genes in the microarray. With opposite signs for correlations are CDKN2D, FOXM1, and BRCA2. The following is a discussion of a selection of probes (highlighted in Figure 3) in the context of prostate cancer.

#### *CDKN2D (Cyclin-dependent kinase inhibitor 2D, p19, inhibits CDK4)*

One of the genes that has strong positive correlations is CDKN2D, (Cyclin-dependent kinase inhibitor 2D, p19, inhibits CDK4) (*Pearson correlation* of 0.7543, *Spearman correlation* 0.6833), probe G_145503. A gene that shows a *positive* correlation with the *divergence* of a metastasis profile indicates a gene that has a putative reduced expression on these samples. CDKN2D is a known regulator of cell growth regulator and controls cell cycle G1 progression [46], [47]. Loss of CDKN2D in cancer cells is one event which is generally associated to a more malignant phenotype.

#### *FOXM1*

Another probe that presents positive correlations is FOXM1 (Forkhead box M1), with Pearson correlation of 0.7039 and Spearman correlation 0.7500), probe G_564803. It has been recently shown that the depletion of FOXM1 still allows cells to enter mitosis but they are unable to complete cell division. As a consequence this leads to mitotic catastrophe or endoreduplication [48]. FOXM1 is considered a key regulator of a transcriptional cluster which is that is essential for proper execution of the mitotic program and the control of chromosomal stability [49].

#### *BRCA2 - (Breast cancer 2, early onset)*

Another gene with positive correlations is BRCA2 (Breast cancer 2, early onset), probe G_193736, with *Pearson correlation* of 0.8161 and *Spearman correlation* 0.7333). While the loss of BRCA2 function and its consequences in prostate cancer is being reconsidered [50], [51], [52], [53], BRCA2 is generally regarded as a “tumor suppressor”, with an established role in maintaining genomic stability via its function in the homologous recombination pathway for double-strand DNA repair. This result is supporting its proposed function. Loss of BRCA2 function is thus a warning sign of the existence of error prone cell processes. In prostate cancer BRCA2 has been associated to promotion of invasion through upregulation of MMP9 [54]. BRCA2 loss of function due to mutations is linked to poor survival in prostate cancer [55] and rare germline mutations have been associated with early-onset of prostate cancer [56].

#### *CCL2/MCP-1 (chemokine (C-C motif) ligand 2)*

Bone is one of the most common sites of prostate cancer metastasis; close to 85% of men who die of prostate cancer have bone metastasis [57]. The successful metastatic process to bone follows from the activation of osteoclasts with bone resorption, which in turns leads to the release of different growth factors from the bone matrix [58]. CCL2 has been previously reported as expressed in human bone marrow endothelial cells; the CCL2 stimulation promotes prostate cancer cell migration and proliferation [57], [59] and it has been proposed as a paracrine and autocrine factor for invasion and growth of prostate cancer [60]. As a consequence of this central role in the tumor microenvironment, CCL2 is being the object of several studies and is included in the list of potential targets for novel therapies [60], [61], [62], [63], [64], [65], [66], [67], [68], [69].

#### *FOS (V-fos FBJ murine osteosarcoma viral oncogene homolog)*

A probe for FOS (G_811015; correlations of −0.9380 and −0.9500 computed with Pearson and Spearman) has a similar correlation than KLK3/PSA. The high rank of FOS was unexpected, but perhaps it is less of a surprise for some experienced researchers in prostate cancer as its role has been highlighted in the past [70], [71], [72]. Amplification of members of the MAPK pathway was associated with androgen independent prostate cancer, and co-expression of RAF1, ERBB2/HER2 and c-FOS would lead to this phenotype [73].

We will not discuss in depth the known relationships between FOS, Lamin A/C and prostate cancer. We leave this discussion for later, as Lamin A/C will also appear in our study of the other prostate cancer dataset studied in this paper. Lamin A/C appears as a member of a set of genes with reduced expression for higher grade primary prostate cancer samples (note that the current analysis that gave FOS as a biomarker is on lymph node metastatic samples like here). However, we would like to point out a connection that is currently hypothesized between Lamin A/C and FOS, the gene we have just discussed. Ivorra et al. have recently proposed that *“lamin A overexpression causes growth arrest, and ectopic c-Fos partially overcomes lamin A/C-induced cell cycle alterations. We propose lamin A/C-mediated c-Fos sequestration at the nuclear envelope as a novel mechanism of transcriptional and cell cycle control”* [*74*]. In addition: “*c-Fos accumulation within the extraction-resistant nuclear fraction (ERNF) and its interaction with lamin A are reduced and enhanced by gain-of and loss-of ERK1/2 activity, respectively.*” [75]. These novel interactions between LMNA and FOS, their putative role in prostate cancer metastasis and their seemingly different behaviours in prostate cancer lymph node metastases warrant further investigation.

#### *SOX9 (SRY (sex determining region Y)-box 9)*

This transcription factor has been recently identified as having an importat role during embryogenesis and in the early stages of prostate development [76], [77] and in testis determination [78], processes that link SOX9 upregulation to cancer development [79]. Basal epithelial cells do express SOX9 in a normal prostate. While there exists no detectable expression in lumina epithelial cells, SOX9 has already been reported as “*expressed in primary prostate cancer in vivo, at a higher frequency in recurrent prostate cancer and in prostate cancer cell lines (LNCaP, CWR22, PC3, and DU145)*” [80]. Wang et al., also in [80] add that: “*Significantly, down-regulation of SOX9 by siRNA in prostate cancer cells reduced endogenous AR protein levels, and cell growth indicating that SOX9 contributes to AR regulation and decreased cellular proliferation. These results indicate that SOX9 in prostate basal cells supports the development and maintenance of the luminal epithelium and that a subset of prostate cancer cells may escape basal cell requirements through SOX9 expression.*” An increased value of SOX9 expression in advanced prostate cancer has been associated to tumor progression and the epithelial-mesenchymal transition [81]. SOX9 expression has been associated with a putative subgroup of prostate cancer [82], associated to lymph-node metastasis (as seems to be the case in this dataset) and has a know role in chondrogenic differentiation processes [83].

#### *KLK3/PSA – (Kallikrein-related peptidase 3)/*Prostate Specific Antigen

To finalize our initial discussion on this dataset, we address KLK3. The high ranking of KLK3/PSA in our list is perhaps one of the most remarkable retrodictive outcomes of our approach. KLK3/PSA (also known as *Prostate Specific Antigen*) is a conspiquous member of our top rank list. It is perhaps the best blood biomarker for prostate cancer screening. Its relevance and popularity as a target of studies is so wide that it makes unfeasible any serious attempt to uncover its relevance in the prostate cancer literature. A search using PubMed using the keyword ‘KLK3’ (and the other alias names of this gene) reveals a total of 11,429 published papers. Of course, many of these publications relate to its role for early screening, but in this study we are uncovering its role as a tissue biomarker. Our results echoes a recent contribution by S. Miyano's and his collaborators [84] on a massive meta-analysis of microarray datasets. It is also in line with results from clinical studies that indicate that a 5-year PSA value is useful for predicting prostate cancer recurrence. Stock et al. recently concluded that “*patients with a PSA value <0.2 ng/mL are unlikely to develop subsequent biochemical relapse*”. Denham et al., studying data from radiation-treated patients on the TROG 96.01 clinical trial, found that on 270 patients there were two distinct “*PSA-signatures*”. These two different dynamical patterns (characterized as “single exponential” or “non-exponential”) stratified the population. Those patients in the second group (50% of the total) “*had lower PSA nadir (nPSA) levels (p<.0001), longer doubling times on relapse (p = .006) and significantly lower rates of local (hazard ratio [HR]: 0.47, 95% confidence interval [0.30–0.75], p = .0014) and distant failure (HR: 0.25[0.13–0.46], p<.0001), death due to PC (HR: 0.20[0.10–0.42], p<.0001) and death due to any cause (HR: 0.37 [0.23–0.60], p<.0001)*” [85]. Certainly the dynamics of PSA, now perhaps with FOS and SOX9 added to the set of biomarkers of interest, warrant further investigation for patient population stratification after initial treatment.

The biomarkers discussed in this section warrant further investigation in prediction of lymph-node metastasis and clinical management of prostate cancer [86], [87], [88], [89], [90], [91], [92], [93], [94], [95], [96], [97], [98], [99], [100], [101], [102], [103], [104], [105], [106], [107], [108], [109]. We refer the reader to the Supplementary Material to have a complete list of probes and their correlations with the Information Theory quantifiers.

### Melanoma – Haqq et al.*'s* dataset (File S2)

The following sections present the results that we obtained with a melanoma dataset. Our aim is to observe if variations of the *Normalized Shannon Entropy* and the statistical complexity measures, *MPR-complexity* and the modified forms *M-normal* and *M-metastases*, provide interesting results in a different disease and experimental setting.

In this case we have selected a gene expression dataset from Haqq et al. [110] containing information of 14,772 cDNAs in 37 samples (Figure two from the [110]). The 37 samples include 3 normal skin, 9 nevi, 6 primary melanoma and 19 melanoma metastases. This datasets has more phenotypical characteristics for the group of samples.

**Figure 4:**
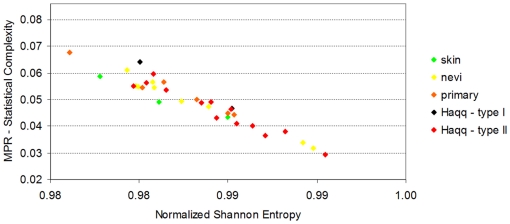
Scatter plot of the samples of the melanoma dataset contributed by Haqq et al. It presents the MPR-Statistical Complexity of each sample as a function of its Normalized Shannon Entropy. This dataset contains information of 14,737 probes and 37 samples. The samples include 3 normal skin, 9 nevi, 6 primary melanoma and 19 melanoma metastases (these samples are 5 of melanoma metastasis ype I and 14 of type II, as labelled by Haqq et al). Following Haqq et al's original classification, the two types of melanoma metastases they identified are presented with different color coding. The plot illustrates that in this case, the Normalized Shannon Entropy does not help to differentiate the normal to metastatic progression (as it happened in the case of prostate cancer). We will show in Figure 5 that the modified statistical complexities M-skin and M-metastasis allow visualizing a clearer transitional pattern.

After an initial process of data cleaning, we removed 35 probes which had an unsually high expression value on only a few samples, in some cases on a single one. The dataset we work with from original contributed by Haqq et al.consists of 14,737 probes. First, we computed the *Normalized Shannon Entropy* and the *MPR-Statistical Complexity* for each sample (refer to the ‘Materials and Methods’ section for a detailed presentation of these calculations). Figure 4 shows the values of these quantifiers for each sample.

We first observe an important difference between Figure 1 and Figure 4. In this melanoma dataset, neither the use of the *Normalized Shannon Entropy* nor the *MPR-complexity* helps to discriminate between normal skin, nevi, primary and metastastic melanomas. Nevertheless, we decided to present this figure for methodological reasons. We envision that some researchers will calculate the *Normalized Shannon Entropy* and *MPR-complexity* using all the probes. We note that in Figure one of Haqq et al's original paper, the whole probe set was previously filtered by selecting those which vary across samples, thus indicating that they may have information about disease subtypes (although the phenotypic types were not biasing the selection). In this case we want to illustrate both the *Normalized Shannon Entropy* and *MPR-complexity* calculated using all the probes does not give the expected benefits. We will now see the benefits of using the *M-complexities*.

As we did for prostate cancer (see Figure 2), we aim at identifying if the use of the modified forms of the statistical complexity (the *M-complexities*) could give some insight where the *Normalized Shannon Entropy* and *MPR-complexity* measures fail. To compute the *M-normal* measure, we need to define the average gene expression profile for a normal cell (which we call *P_ave_*). We thus resort to the three normal skin profiles and we produce the average based on these profiles (details for computing the average profiles are given in the ‘Materials and Methods’ section). We call *M-skin* the resulting measure that relies on this profile. Analogously, we need to compute a pattern for *M-metastasis*, and we proceed to calculate the *P_ave_* profile averaging over the 19 metastases samples. The result is encouraging, as samples plotted in the (*M-skin*, *M-metastasis*)-plane cluster in groups, showing an important *M-skin* complexity transition between normal skin cells and nevi. Most importantly, this method naturally shows that some of the metastatic samples have a large value of *M-skin* complexity, so we present the results of another experiment, aimed at clarifying this fact.

In their original publication, Haqq et al. classified the melanoma metastases in two groups due to their molecular profiles: five samples were classified as ‘Type I’ and fourteen as ‘Type 2’ based on a hierarchical clustering approach. Our result reinforced the view that the Type II melanomas metastasis is a pretty homogeneous group, we will present the results on the (*M-skin*, *M-metastasis I*)-plane. This means that now the *P_ave_* profile will not be obtained by averaging over the 19 metastases samples, but instead using only the 14 samples which have been labelled as ‘Type II’. As such, we aim at revealing if Type I samples are indeed different in this plane, and if other clusters are also present.

**Figure 5:**
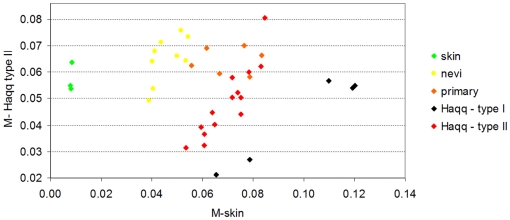
Scatter plot of the melanoma sample dataset of Haqq et al. This is the same set of samples of Figure 4 and we have used the same color coding. We are now using the modified statistical complexity measures *M-skin* and *M-metastasis II*. As expected, normal skin samples (in green) have a low value of the *M-skin* measure. Interestingly, most of the nevi samples (in yellow) have an intermediate value of the *M-skin* measure, and most of the primary and metastatic samples have even larger values of *M-skin*. This result, together with our observation and analysis of Figure 4, indicate that the *Jensen-Shannon divergence* of melanoma samples from the normal skin profile may be a relevant measure to quantitatively analyse progression even when the whole gene expression dataset is used. We observe that, although the *M-metastasis II* measure has used all the samples labelled as Type 2 (in Haqq et al.*'s* original contribution), their position in this plane shows two different clusters. This may indicate that a further heterogeneity may exist in this subgroup, a fact that warrants further study with a larger group of samples.

Figure 5 presents the results. The first fact worth commenting is the pronounced gap between normal skin samples and the nevi, primary, and metastatic melanoma samples as revealed by the *M-skin* measure. Note also that the *M-skin* is based on the average profile that of the normal samples, which indicates that no information about the profiles of metastasis are used, yet *M-skin* reveals that increasing values of this measure may be linked with a ‘progression’ from nevi to primary and metastasis melanoma profiles.

We now introduce another useful technique to identify genes which correlate with the transitions. The challenge is to find genes which are related with the progression towards metastases profiles, even when we recognize that there the group of metastasis samples is heterogeneous (containing at least two groups). Since the final outcome of Figure 4 and Figure 5 is that the *Normalized Shannon Entropy* does not help much in this experimental scenario, we will concentrate only on one of the multiplicative factors of the *M-complexities*, the *Jensen-Shannon divergence*. We compute two *P_ave_* profiles, one with the normal skin samples only, and the other with all the metastasis samples (regardless their type). We will call the two divergences *JSM0* and *JSM5* respectively. We then compute the Spearman correlation of the profile of all gene probes in the array across the 37 samples to both *JSM0* and *JSM5*. We have listed all probes according to the absolute value of the difference of these correlations, i.e. *Abs. Diff. (probe) = |JSM0(probe)−JSM5(probe)|* in decreasing order. The results are provided as Haqq-PLoSONE-SupFile.xls, in the sheet labelled ‘Results-correlation’.

**Figure 6:**
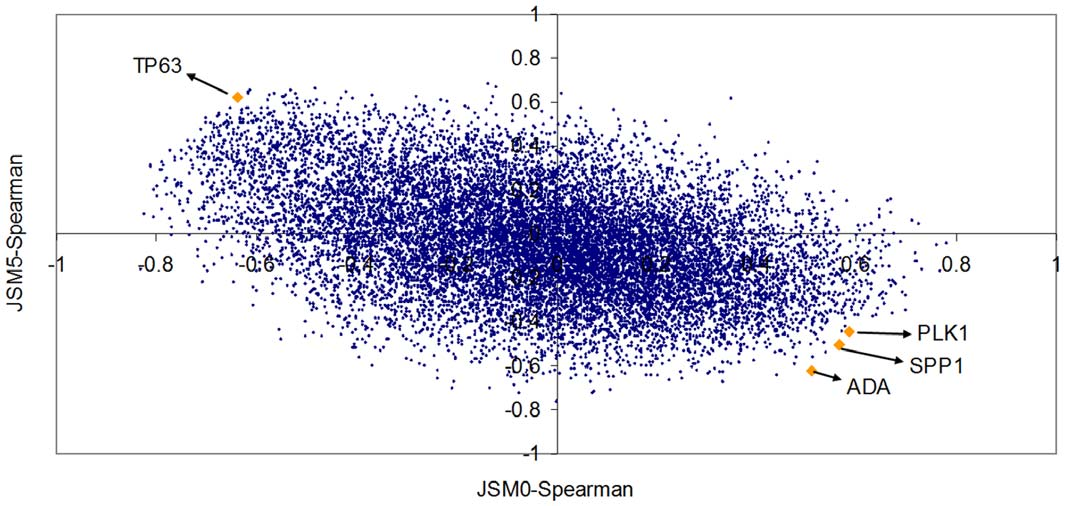
A scatter plot of the Spearman correlation of 14,737 probes in the Haqq et al. melanoma dataset. We have computed the *Jensen-Shannon divergence* of each sample with the normal skin average. We then computed the correlation of each individual probe expression with the *Jensen-Shannon divergence* of each sample. As this correlation is computed on all samples, the resulting value (x-axis) was denoted as *JSM0A-Spearman*. Analogously, we compute the *Jensen-Shannon divergence* of each sample with the average metastastic profile and we also compute the correlation of each probe with this measure (y-axis). The position of one probe corresponding to the TP63 gene (Tumor protein p63, keratinocyte transcription factor KET), AA455929, is highlighted. The expression of this probe has a relatively high negative correlation with the *Jensen-Shannon divergence* of the normal skin type (*JSM0-Spearman* = −0.63632) while at the same time is has a positive correlation with the *Jensen-Shannon divergence* of the metastasis profile (*JSM5* = 0.62138). The first probe that presents an opposite behaviour is one for ADA (Adenosine deaminase), AA683578. Probes for SPP1 (Secreted phosphoprotein 1 or Osteopontin) and PLK1 (Polo-like kinase 1 or Drosophila) are also highlighted. While PLK1 is currently less recognized as a biomarker in melanoma research, the importance of SPP1 in cutaneous pathology [315], [318], [320], [321] and in particular in melanoma [208], [209], [210], [211], [212], [214], [215], [216], [217], [218], [219], [222], [226], [264], [314], [315], [316], [317], [319], [322], [323], [324], [325], [326], [327], [328], [804], [805], [806], [807], [808], [809] is increasing. Using a 5-biomarker panel that included SPP1, Kashani-Sabet et al. used tissue microarrays on 693 melanocytic neoplasms to show that SPP1 expression collaborates significantly improving the detection of high percentage of melanomas arising in a nevus, Spitz nevi, dysplastic nevi and misdiagnosed lesions [253]. Like in the case of prostate cancer (Figure 3, in which KLK3/PSA - Prostate Specific Antigen was highlighted), our method allows the detection of important biomarkers with a high degree of concordance with current biological understanding of metastatic processes.

The rationale is to identify those probes which are highly correlated (both positively or negatively) with the *Jensen-Shannon divergence* of the normal tissue profile and that “*reverse signs*”. For instance, a probe for the TP63 gene (Tumor protein p63, keratinocyte transcription factor KET), AA455929, is ranked in the third position. Its correlation with the *Jensen-Shannon divergence* of the normal skin type is relatively high and negative (*JSM0* = −0.63632) while at the same time is has a positive correlation with the *Jensen-Shannon divergence* of the metastasis profile (*JSM5* = 0.62138). In the ranking, the first probe that presents the *opposite* behaviour is one for ADA (Adenosine deaminase), AA683578. Figure 6 helps to understand the relationship of these correlations with expression. Not only are these genes well correlated with the divergences, they also seem to be good markers of the progression from one tissue type profile to the metastasis profile.

**Figure 7:**
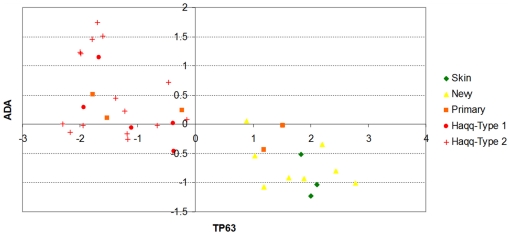
Scatter plot showing the expression of the probe corresponding to ADA (Adenosine deaminase), AA683578 (y-axis) and TP63 (Tumor protein p63), AA455929 (x-axis). All the samples that have TP63 expression are normal or nevi, with two primary melanomas still preserving TP63 expression but with higher ADA. The trend reverses for the rest of the primary melanoma samples and the metastatic ones, which all express ADA but not TP63.

We will now discuss three of these genes in the context of current biological knowledge on melanoma drivers and metastatic progression. We provide many references for one of them, SPP1 (Secreted phosphoprotein 1 or Osteopontin). The discussion on this gene will be left for later, when we will discuss specifc oncosystems related to cell proliferation, chemotaxis and responses to external simulus. Figure 7 shows the expression of ADA (Adenosine deaminase, AA683578) as a function of TP63 (keratinocyte transcription factor KET, AA455929). All normal skin samples, as well as nevi and a couple of primary melanomas have relatively low values of ADA but they express TP63. There is a change of roles in metastatic and some primary melanomas, which have reduced TP63 expression but increased values of expression of ADA. As we will later see, these events correlate with other major transcriptional modifications which involve dozens of genes and that we have been able to map thanks to functional genomics bioinformatics tools. The role of SPP1 will be discussed in that context after some references to TP63, ADA, and PLK1 which follow.

#### *TP63*

The product of this gene [111], [112] belongs to the same protein family of its more famous relative, TP53, a gene that is often mutated in human cancers [113] and highly regarded as a key “tumor suppressor”. TP63's product, p63, is a homologous protein to p53, which is considered to be phylogenetically newer [114] and also regarded as an important apoptotic and cell-cycle arrest protein. Mice that lack TP53 are born alive with a propensity for developing tumours; mice that lack TP63 do not appear to be tumour prone, although, new results are partially contradicting earlier findings [115]. It appears that the diverse roles of the isoforms of the p63 family reveal that there exists a crosstalk with the different isoforms of the p53 family that needs to be systematically investigated [116]. It has recently been shown that p63 is a key regulator of the development of stratified epithelial tissues [113] and that its deletion results in loss of stratified epithelial and of all keratinocytes [117]. Melanocytes also express two isoforms of p63 [118], but p63 expression is not reported in 57 out of 59 tumors in a tissue microarray study performed by Brinck et al. [119]. It is clear that the the role of loss of expression of TP63 in melanoma warrants further investigation.

#### *ADA - (Adenosine deaminase) and DPP4/CD26 (Dipeptidyl-peptidase 4, CD26, adenosine deaminase complexing protein 2)*

A link between TP63 and ADA has already been reported in the literature. ADA is a gene involved in cell division and proliferatation [120] and it has been suggested to have a regulatory role in dendritic cell innate immune responses [121].Translational modification is also a function of p63. Sbisa et al. have proved that ADA is a direct target of isoforms of p63, which is an important discovery as ADA has two TP53 binding sites, leading to a complex metabolic balance due to the different relationships between this trio and p21 yet to be completely elicitated [120], [122]. Several studies indicate elevation of adenosine deaminase levels in sera of breast [123], head and neck [124], colorectal [125], acute lymphoblastic leukaemia [126] and laryngeal cancers [127].

We observe a marked increase of expression of a probe for ADA with melanoma progression while at the same time we observe a loss of expression of a probe corresponding to DPP4/CD26 (Dipeptidyl-peptidase 4, CD26, adenosine deaminase complexing protein 2), a membrane-bound, proline-specific serine protease [128] that has been attributed tumor suppressor functions [129]. It has been previously reported that loss of DPP4 immunostaining helps to discriminate malignant melanomas from deep penetrating nevi, a variant of benign melanocytic nevus [130] and early reports of their absence in metastatic melanomas exist [131], [132]. As deep penetrating nevi can mimic the vertical growth phase of nodular malignant melanoma, and ADA could potentially be downregulating DPP4 [133], [134] we believe that the elicitation of the complementary role of these two biomarkers to distinguish these two entities is necessary and also warrants further clinical studies.

#### *PLK1 (Polo-like kinase 1 (Drosophila))*

Another probe for gene that ranks high as a positive marker of metastasis is PLK1, Polo-like kinase 1, Serine/Threonine protein kinase 13 (AA629262). PLK1 is a centrosomal kinase [135] which is regarded as being linked to centrosome maturation and spindle assembly [135]. PLK1 expression has also been singled out as a biomarker of a “*death-from-cancer*” signature, sharing with others the function of being an activator of mitotic spindle check point proteins. With other proteins it would has a stem cell-like expression profile phenotypically characterized by enabling metastasis with anoikis resistance and disregulated cell-cycle control [136]. PLK1 inhibition could be a common target for gastric adenocarcinoma [137], bladder cancer [138], colon cancer [139], [140], hepatocellular carcinoma [141], medullary thyroid carcinoma [142], esophageal cancer [143], pancreatic cancer [144] and in some types of non-Hodgkin lymphomas [145] and breast cancer [146].

PLK1's Spearman correlation with the values of the *Jensen-Shannon divergence* of samples with the normal skin profile is relatively high (0.5863). PLK1 also has a high value of (negative) Spearman correlation with the values of the *Jensen-Shannon divergence* of samples with the average metastatic profile (−0.44571). In 2002 Kneisel et al. have conducted a study to investigate the expression of PLK1 in very thin melanomas (smaller or equal to 0.75 mm). On 36 patients, within five-years of follow-up, 22 melanomas developed metastases while 14 did not. In the comparison, it was found that metastatic malignant melanomas with expressed PLK1 at markedly elevated levels (median, 60.00% vs. 37.98%; *p*-value<0.000053), concluding that PLK1 is a reliable biomarker for patients at high risk of metastases, even when the most important prognostic clinical factor (Breslow's maximum thickness of the primary malignant melanoma) indicates the contrary [147]. We consider this an important finding as PLK1 silencing is already part of an integrated oncolytic adenovirus approach currently being studied in mice models of orthotopic gastric carcinoma [148] and has promise due to the lack of a reported measurable immune response of siRNA-based therapeutics [149]. Another positive note is the less sensitivity to PLK1 depletion of cells with a functional p53 [150], [151], and can help to sensitize cells to chemotherapy (as observed in lung cancer [152]). This constraint of aneuploid cancer cells to PLK1 expression, particularly in cells with inactivated p53 [153], could be exploited by lentivirus-based RNA interference [154].

*Correlation analysis with Jensen-Shannon divergences reveals biomarkers for loss of cell adhesion, cell-cell communication, impairment of tight junction mechanisms and dysregulation of epithelial cell polarity.*

As discussed before, the probe for ADA (Adenosine deaminase) is the first that has a different trend. Since we put all metastasis samples together in the same group when we calculated the average probability profile (and we have a heterogeneous group) we have on our ranking 58 probes that appear before ADA (we refer to the Supplementary File Haqq-PLoSONE-SupFile.xls). An analysis using GATHER (http://gather.genome.duke.edu/) [155] to interpret the collective influence of the lack of expression of all these genes in the metastasis samples reveals an interesting new perspective. Using Gene Ontology, we found that six of the 44 genes identified by GATHER are related to epidermis development (CDSN, DSP, EVPL, GJB5, KRT13, KRT5), *p-value<0.0001*, *Bayes Factor 16*, and eight genes are related to cell adhesion (CDSN, CLDN1, DSG1, DST, LGALS7, LRIG3, PCDH21, PKP1), *p-value<0.0001*, *Bayes Factor 7*. ANK1 (Ankyrin 1, erythrocytic), AA464755 was also singled out as by our Gene Ontology analysis as related to the maintenance of epithelial cell polarity (*p-value = 0.002*, *Bayes Factor 3*). The use of another profiler of genome signatures (g:Profiler, [156]) also reinforces the view that many genes that have lost expression are related to ‘*Epidermis Development*’ (COL17A1, DSP, EVPL, GJB5, KRT13, KRT5, LCE1C, MAFG, TGM3) with *p-value = 7.78E-11*. Thirteen are associated with Gene Ontology function of cell communication (ANK1, CDSN, CLDN1, DSG1, DST, GCHFR, GJB5, GPR115, LGALS7, LRIG3, PCDH21, PKP1, PTGER3), albeit with a *p*-value of only 0.02. GCHFR is also involved in nitric oxide metabolism.

If we add to the list of 44 genes already recognized by GATHER the other 77 probes that after ADA in this ranking have also loss of expression (until we found PDXP (Pyridoxal (pyridoxine, vitamin B6) phosphatase), the evidence is stronger, now COL7A1, GJB5, KLK4, and KRT1 also is in this group (the Bayes factor of this association returned by Gather is now 21 for the GO term ‘*Epidermis development*’). ‘*Cell adhesion*’ has now 13 genes, CDSN, CLDN1, COL7A1, DSC2, DSG1, DST, JUP, LGALS7, LRIG3, PCDH21, PKP1, SLIT3 THBS3 (p-value<0.001, Bayes factor 10). These results are considered statistically very relevant as identifiers of a particular process which seems to be undermined by this collective loss of expression.

**Figure 8:**
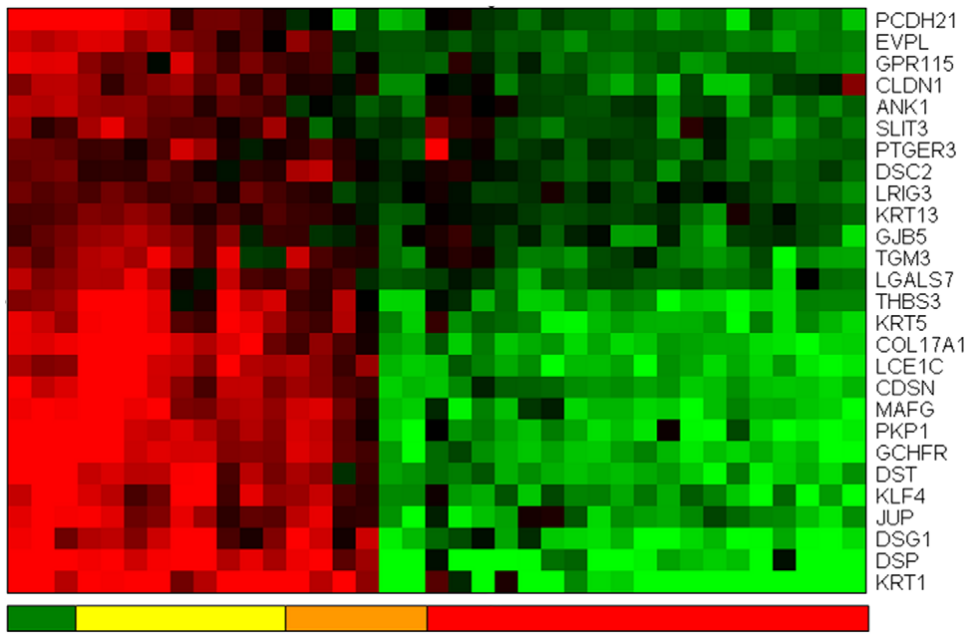
Heat map of the expression of 27 probes with genes annotated showing functions on *cell adhesion*, *cell-cell communication*, *tight junction mechanisms* and *epithelial cell polarity*. The average expression of the skin samples is shown in green. In yellow, the nevi samples, showing that some of them have a reduced average expression. The primary melanomas have a mixed behaviour (orange columns) with four of them having almost zero of negative average expression. The metastatic samples (columns in red) have all a negative average expression. Overall the figure indicates a progression, from the positive average expression of this gene panel for nevi and normal skin samples, towards negative expression values of the metastatic samples, “passing” through the mixed behaviour present in primary melanomas.

If we put all this information together, we clearly observe a pattern of downregulation of gene expression that is associated with an impairment of epidermis development and the maintainance of its structure (Figure 8 and Table 1). This is, perhaps, an instantiation of one of the “extended hallmarks of cancer” (that of “*tissue dedifferentiation*”). This process includes the loss of function of genes that are essential for the maitainance of tight junction and epithelial cell-cell communication. While loss of epithelial structure is related to these genes, we observe that those that increase expression are associated to other developmental processes, not necessarily concerted in this panel. Instead they show a pattern of increasing cell motility, chemotaxis and positive regulation of cell proliferation. We will first discuss the processes related to the loss of adhesion, which could be linked to an increased probability of metastatic potential of these cells.

**Table 1:** Gene names and probe accession number of the 27 probes with genes annotated with functions on *cell adhesion*, *cell-cell communication*, *tight junction mechanisms* and *epithelial cell polarity* shown in the heat map in Figure 8.

**THBS3**	NM_007112	Hs.169875	Thrombospondin 3
**TGM3**	AK290324	Hs.2022	Transglutaminase 3 (E polypeptide, protein-glutamine-gamma-glutamyltransferase)
**SLIT3**	BC098388	Hs.604116	Slit homolog 3 (Drosophila)
**PTGER3**	NM_198715	Hs.445000	Prostaglandin E receptor 3 (subtype EP3)
**PKP1**	NM_000299	Hs.497350	Plakophilin 1 (ectodermal dysplasia/skin fragility syndrome)
**PCDH21**	NM_033100	Hs.137556	Protocadherin 21
**MAFG**	NM_002359	Hs.252229	V-maf musculoaponeurotic fibrosarcoma oncogene homolog G (avian)
**LRIG3**	AY358288	Hs.253736	Leucine-rich repeats and immunoglobulin-like domains 3
**KRT**	5M21389	Hs.433845	Keratin 5 (epidermolysis bullosa simplex, Dowling-Meara/Kobner/Weber-Cockayne types)
**LGALS7**	BM913998	Hs.558355	Lectin, galactoside-binding, soluble, (galectin 7)
**LCE1C**	NM_178351	Hs.516429	Late cornified envelope 1C
**KRT13**	CR591347	Hs.654550	Keratin 13
**JUP**	BX648177	Hs.514174	Junction plakoglobin
**GPR115**	NM_153838	Hs.710050	G protein-coupled receptor 115
**GJB5**	AK129509	Hs.198249	Gap junction protein, beta 5, 31.1kDa
**GCHFR**	BQ054887	Hs.631717	GTP cyclohydrolase I feedback regulator
**EVPL**	NM_001988	Hs.500635	Envoplakin
**DST**	NM_183380	Hs.631992	Dystonin
**DSP**	NM_004415	Hs.519873	Desmoplakin
**DSG1**	NM_001942	Hs.2633	Desmoglein 1
**DSC2**	BC063291	Hs.95612	Desmocollin 2
**COL17A1**	NM_000494	Hs.117938	Collagen, type XVII, alpha 1
**CLDN1**	NM_021101	Hs.439060	Claudin 1
**CDSN**	NM_001264	Hs.556031	Corneodesmosin
**ANK1**	NM_000037	Hs.654438	Ankyrin 1, erythrocytic

*The loss of expression of Plakophilin 1, Junction plakoglobin, Desmoplakin and Desmoglein 1 indicate deficiencies in desmosome processes.*

In general, this panel is composed of a number of genes that are losing expression during progression and that have Gene Ontology annotations related to tight junctions, gap junctions, adherens junctions and desmosomes, and an impaired set of processes that link, via intercellular channels and bridges, the cells of the epidermis. Mutations in these genes are linked to a number of skin genetic diseases [157], [158], [159], [160], [161], [162], [163], [164], [165], [166], [167], [168], [169], [170]

The desmosome are cell-cell adhesive junctions which provide a mechanical coupling between cells. These junctions are found in several epithelial tissues and the decreased assembly of the desmosome has been shown to be a common feature of many epithelial cancers [171], [172]. Plakoglobin helps to connect transmembrane elements to the cytoskeleton [173]. Plakophilin 1 [174] (PKP1, one of the genes in our panel above) is a desmosomal plaque component [175] that stabilizes desmosomal proteins at the plasma membrane [176], [177] and, with desmoplakin [178], recruits filaments to sites of cell-cell contacts [179]. As a consequence, it has been proposed that the lack of PKP1 increases keratinocyte migration [180] and loss of PKP1 expression in head and neck squamous cell carcinoma and in esophageal squamous cell carcinoma may contribute to an invasive phenotypic behaviour [171], perhaps as a consequence of the impaired recruitment of desmoplakin.

The desmoglein-specific cytoplasmic region (DSCR) is the site of caspase cleavage during apopotosis and is a conserved region of yet undefined function and unknown structure, but it specifies the function of the desmoglein family of cell adhesion molecules (of which DSG 1 is a member). It has been recently shown that the DSCR has a weak interaction with PKP1, Plakophilin 1 (ectodermal dysplasia/skin fragility syndrome) and the cytoplasmic domain of Desmocollin 1 [181]. Plakoglobin is cleaved by Caspase 3 during apoptosis [182]. In addition, Kami et al. in Ref [181] also report and conclude that: “*desmoglein 1 membrane proximal region also interacts with all four DSCR ligands, strongly with plakoglobin and plakophilin and more weakly with desmoplakin and desmocollin 1. Thus, the DSCR is an intrinsically disordered functional domain with an inducible structure that, along with the membrane proximal region, forms a flexible scaffold for cytoplasmic assembly at the desmosome*”.

**Figure 9:**
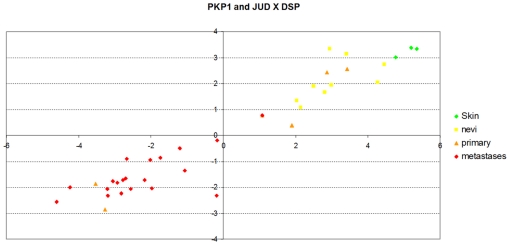
Shows the average expression of PKP1 and JUP. The joint expression of the probe for PKP1 (Plakophilin 1 - ectodermal dysplasia/skin fragility syndrome - NM_000299) and the probe for JUP (Junction plakoglobin - BX648177), as added values on the *x*-axis, against the expression of the probe for DSP (Desmoplakin - NM_004415 Hs.519873) on the *y*-axis. There is a clear common downregulation trend of these biomarkers from the normal skin (Skin) to the nevi (MN) and to the primay melanoma and metastic melanoma samples (PM and MM respectively).

As previously discussed, all these genes progress towards a loss of expression, and they are highly correlated. Figure 9 shows the average expression of PKP1/Plakophilin 1 (ectodermal dysplasia/skin fragility syndrome), (NM_000299) and JUP, Junction plakoglobin, (BX648177) on the x-axis against that of DSP, Desmoplakin (NM_004415 Hs.519873) on the y-axis. Again, we see a clear pattern of progressive reduction of expression from normal skin and nevi (green and yellow, respectively), primary melanomas (in orange) and melanoma metastases (red).

*Joint loss of expression of Claudin 1 and members of the Aquaporin family are also linked to a transition to a more malignant phenotype*

**Figure 10:**
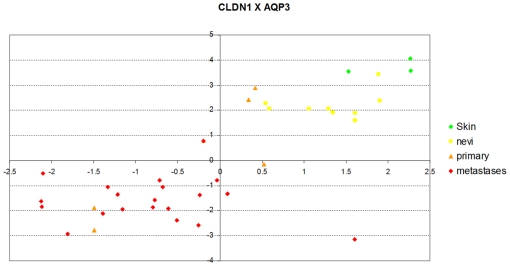
Expression of a probe for CLDN1 (Claudin 1) (*y*-axis) as a function of a probe for Aquaporin 3 (*x*-axis). Other members of the aquaporin family of proteins have a similar behaviour. AQP3, together with CLDN1 are key components of the tight junction complexes of the epidermis and their joint loss of expression seem to be related to a transition to a more malignant phenotype. We use the same color coding as Figure 9.

We note however, the Gene Ontology annotation is not the only way that we can make sense of this information. A detailed analysis of that list of 58 genes reveals other proteins involved in tight junction, like Aquaporin 3 (AQP3). Probes for AQP3 and Claudin 1 (CLDN1) have reduced expression with the progression of the disease as shown in Figure 10.

**AQP3 (Gill blood group)** is a member of the aquaporin family of proteins, and currently is recognized as an ‘aquaglyceroporin’ [183] of great importance to maintain skin hydration of mammals epidermis [184]. Three proteins of this family (AQP1, AQP3, and AQP9) have probes that seem correlated with melanoma progression, all losing their expression in the process of going from normal skin to metastatic melanoma. AQP3 water channels have been pointed out as an essential pathway for volume-regulatory water transport in human epithelial cells [185]. AQP3 is also selective for the passage of glycerol and urea and it has been suggested that osmotic stress up-regulates AQP3 gene expression in cultured keratinocytes [186]. AQP3 was found to be the predominant aquaporin in human skin which increased expression and altered cellular distribution of AQP3 in eczema thus contributing to water loss [187]. The putative involvement of aquaporins in the progression of melanoma, uncovered by our method in our results, warrants further investigation as it has been recently shown that another member of this family (AQP8) also facilitates hydrogen peroxide diffusion across membranes [188]. It is suspected that AQP3 has other functions with a suggestion that it is involved in ultraviolet radiation induced skin dehydration [189]. There is no probe for AQP8 in Haqq et al.*'s* dataset that we could scrutinize from its trend with progression but we note that a novel strategy for drug development for melanoma (i.e. Elesclomol) works by inducing apoptosis via a mechanism of elevation of reactive oxygen species (of course, including hydrogen peroxide in cancer cells) thus exploiting the “*Achilles hell of cancer metabolism*” [190].

**Claudin 1, CLDN1** [191], a gene which is reported to be “*normally expressed in all the living layers of the epidermis*” [192], in concert with AQP3, is a key component of the tight junction complexes of the epidermis. Low CLDN1 gene expression was correlated with shorter overall survival in lung adenocarcinoma. Overexpression of CLDN1 was correlated with suppression of cancer cell migration, invasion and metastasis [193]. Hoevel et al. report that re-expression of CLDN1, in breast tumor spheroids, induces apoptosis and they conclude: “*These findings support a potential role of the tight junction protein CLDN1 in restricting nutrient and growth factor supplies in breast cancer cells, and they indicate that the loss of the cell membrane localization of the tight junction protein CLDN1 in carcinomas may be a crucial step during tumor progression*” [194]. Tokes et al.also report that malignant invasive breast tumors are negative for CLDN1 [195]. As in breast cancer [196], in which reduced expression correlated with recurrence status, the low expression of CLDN1 and other tight junction proteins seems to contribute to cellular detachment.

*The complementary set of correlations with the Jensen-Shannon divergences unveils biomarkers for cell proliferation, chemotaxis, and responses to external simulus.*

If the use of Gene Ontology has produced very peculiar results, helping us to link the loss of expression of 44 genes with a significant change in epithelial structure and development. A natural question arises: “*Which is the significance of another set, now arbitrarily chosen to be also of the same cardinality (i.e 44 genes) with the complementary behavioural pattern?*” We have now listed all the probes according to *Diff. (probe) = JSM0(probe)−JSM5(probe)* in decreasing order. The results are provided as Haqq-PLoSONE-SupFile.xls (‘Results-correlation’ sheet). This now gives ADA as the first ranked gene. Again using GATHER [155] on the first 44 genes recognized by the software, and again using Gene Ontology, we observe as most important common function that of *cell motility* (CCL3, CXCL10, FPRL1, SEMA6A, SPP1), *p-value = 0.0002*, *Bayes Factor 5*, and *chemotaxis* (CCL3, CKLFSF7, CXCL10, FPRL1, SPP1), *p-value<0.0001*, *Bayes Factor 7*. The genes CXCL10, SPP1, and WARS, together with another gene that has been annotated as related to *positive regulation of mitosis* (SCH1), have also been annotated as *regulators of cell proliferation* (*p-value = 0.007*, *Bayes Factor 2*). Using the g:Profiler software [156], we obtain a complementary information. Sixteen genes (including SPP1, SEMA6A, LEF1 [197], CD230, ALS2CR2, DKK1, CYFIP2, SHC1, ANKRD7, IFI6, CITED1, and MID1) have been associated to the Gene Ontology term of ‘*developmental process*’.

#### *SPP1 - Secreted phosphoprotein 1 (osteopontin)*

SPP1 is one of the most conspicuous melanoma biomarkers [198], [199], [200], [201], [202], [203], [204], [205], [206], [207], [208], [209], [210], [211], [212], [213], [214], [215], [216], [217], [218], [219], [220], [221], [222] (see also the references cited in Figure 6 and note its eminent position in this scatter plot). In 1990, Craig et al. reported that SPP1 may work as an autocrine adhesion factor for tumor cells (see also [204], [223], [224]). They observed that “*SPP1 mRNA, which is barely detectable in normal mouse epidermis, was expressed at moderate-to-high levels in 2 of 3 epidermal papillomas and at consistently high levels in 7 of 7 squamous-cell carcinomas induced by an initiation-promotion regimen*” [225]. The evidence is being constantly expanded on the role of SPP1 as a molecular prognostic biomarker in melanoma [226]. Activation of SPP1 may be an important event that allows the transformed melanocytes to invade the dermis as proposed by Geissinger et al. in 2002 [208]. This causes SPP1 to avoid the apoptotic stimulus, one of the “hallmarks of cancer”, which invasive cells will be receiving from this new tissue.

If we extend the literature-based search so that we now include the first 200 gene probes recognized by GATHER then we have 27 gene probes associated with the Gene Ontology in terms of “*cell proliferation*” (*p-value = 0.0002*, *Bayes Factor 5*), and ‘*regulation of cell proliferation*’, *p-value = 0.003*, *Bayes factor 3*). However, other partners of PLK1 appear and their function in ‘*mitotic cell cycle*’ (*p-value = 0.0003*, *Bayes Factor 5*) is increasingly present (in particular, the M phase of the mitotic cell cycle). The details of the Gene Ontology terms which are significant and the genes associated to them are listed in Table 2.

**Table 2:** Significant Gene Ontology terms and their associated genes.

Gene Ontology annotation	Genes	p-value	Bayes factor
GO:0008283 [4]: cell proliferation	27 (AURKB BCCIP BST2 BUB1 CCT4 CDC7 CDCA5 CENPF CHEK1 CXCL1 CXCL10 DNAJC6 FLT1 FTH1 IFI16 KIF23 LIG3 MCMDC1 PLK1 PSEN2 PTTG1 SHC1 SLAMF1 SPP1 STK6 TFDP1 WARS)	0.0002	5
GO:0000278 [6]: mitotic cell cycle	10 (BCCIP BUB1 CDC7 CENPF CHEK1 KIF23 PLK1 PTTG1 SHC1 STK6)	0.0002	5
GO:0000280 [7]: nuclear division	9 (BUB1 CENPF CHEK1 KIF23 LIG3 PLK1 PTTG1 SHC1 STK6)	0. 0003	4
GO:0000279 [6]: M phase	9 (BUB1 CENPF CHEK1 KIF23 LIG3 PLK1 PTTG1 SHC1 STK6)	0.0004	4
GO:0007067 [8]: mitosis	7 m(BUB1 CENPF KIF23 PLK1 PTTG1 SHC1 STK6)	0.003	3
GO:0042127 [5]: regulation of cell proliferation	10 (CDC7 CHEK1 CXCL1 CXCL10 FLT1 FTH1 SHC1 SLAMF1 SPP1 WARS)	0.003	3
GO:0000087 [7]: M phase of mitotic cell cycle	7 (BUB1 CENPF KIF23 PLK1 PTTG1 SHC1 STK6)	0.003	3
GO:0006928 [4]: cell motility	8 (ARPC1B ARPC2 CCL3 CXCL10 FPRL1 NRP2 SEMA6A SPP1)	0.004	2

The analysis using g:Profiler largely coincides with the analysis using GATHER, however, it retrieves 12 genes associated with the M phase of mitotic cell cycle, namely: AURKA and AURKB [227], [228], [229], BUB1 [230], [231], CDCA5A/Sororin/p35 [232], CDC7 [233], [234], CHEK1 [235], KIF23/MKLP-1 [227], [236], [237], MAP9/ASAP [238], [239], NCAPD3, NCAPG2 [240], NEK6 [241], [242], [243], [244], PLK1 [147], [245], [246], PTTG1/Securin [247], SHC1/p66 [248], [249], [250] (discussed in the context of SHC4 signalling), and TFDP1/DP-1 [251]. These are a significant finding by g:Profiler (*p-value* = 4.03E-07).

**Figure 11:**
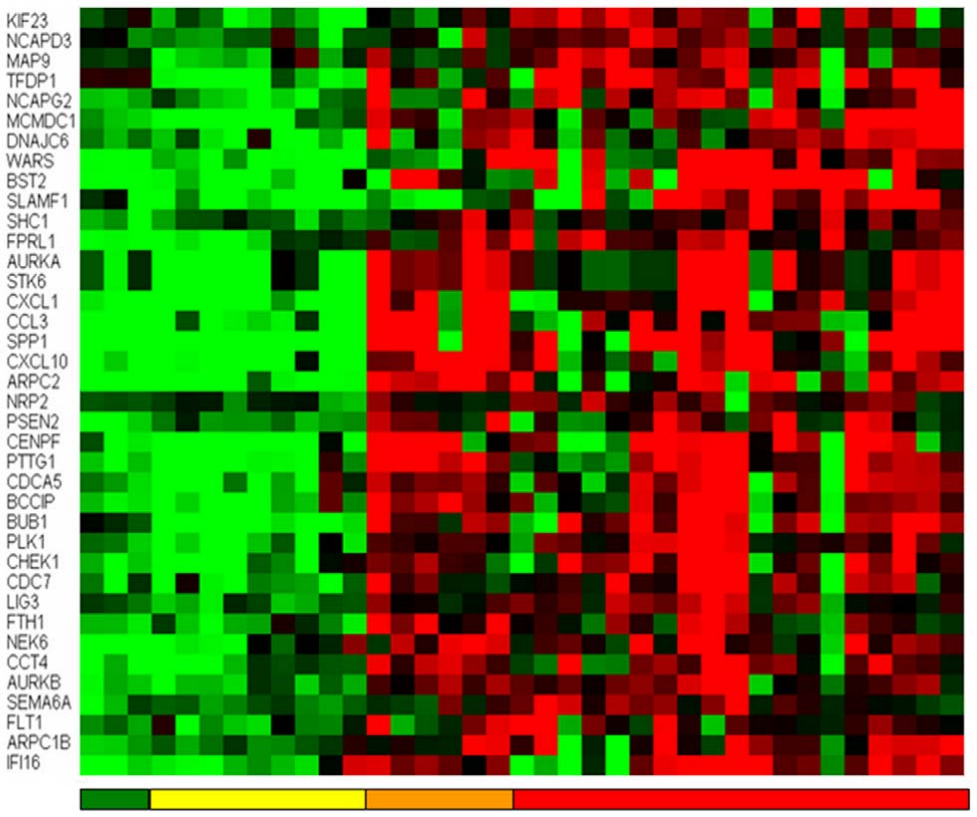
Heat map of the expression of 38 gene probes annotated with functions on *cell proliferation*, in particular *cell motility*, *mitotic cell cycle*, *nuclear division*, and specifically, *M phase of mitotic cell cycle*. We have used the same convention we employed in Figure 8: in green, the normal skin samples; in yellow, the nevi samples; the primary melanoma samples (in orange) show increased expression for most of these biomarkers. This may indicate that the upregulation of genes involved in these processes is an earlier event (it occurs as a common feature in all the primary melanoma samples) while modifications to *cell adhesion*, *cell-cell communication*, *tight junction mechanisms* and *epithelial cell polarity* occur later (primary melanomas in Figure 4 show a transition). Finally, the metastatic samples (in red) show some heterogeneity, but overall provide an increased expression. The average expression of this panel could be a good indicator of the transition from nevi to a malignant phenotype, while the panel of Figure 8 can complement the information indicatingthe onset of tissue dedifferentiation processes.

We have listed above some of the genes gene associated to the M phase of mitotic cell cycle and associated references which are either to current research in melanoma and/or its biological function. We now list other genes which have been associated with the term ‘cell proliferation’ by GATHER. These genes are: ARPC1B [252], ARPC2 (which, together with SPP1, is also in the novel 5-biomarker panel of Kashani-Sabet et al. [253]), BCCIP (BRCA2 and CDKN1A-interacting protein)/P21-and CDK-associated protein 1) [254], BST2/Bone marrow stromal antigen 2/Tetherin [255], CCL3/MIP-1alpha [256], [257], [258], CCT4, CDCA5/Sororin [259], [260], [261], [262], [263], CENPF/Mitosin [264], CXCL1/chemokine (C-X-C motif) ligand 1 (melanoma growth stimulating activity, alpha) [265], [266], [267], [268], [269], [270], [271], [272], [273], [274], [275], [276], [277], [278], [279], [280], [281], [282], [283], [284], [285] (in uveal melanoma see [286]), CXCL10 [256], FLT1/VEGFR1 [287], [288], [289], [290], [291], [292], [293], [294], [295], [296], [297], [298], [299], FTH1/Ferritin Heavy Chain [300], [301], [302] (which may indicate a necessary condition for the mainainance of iron sequestration and suppression of reactive oxygen species accumulation [303]), FPRL1, LIG3/DNA Ligase 3 [304] (which, together with XPA and ERCC5 is associated to DNA repair in ionizing radition studies [305]), MCMDC1, PSEN2, NRP2/Neuropilin 2/Vascular endothelial cell growth factor 165 receptor 2 [306], [307], [308], SEMA6A (a member of the Semaphorin family, of increasing importance in cancer research [309], [310], [311] and in particular due to its observed upregulation in undifferentiated embryonic stem cells [312]), SLAMF1/CD150 (a marker associated with hematopoietic stem cells [313]), SPP1/Osteopontin (which, together with ARPC2, is also in the novel 5-biomarker panel of Kashani-Sabet et al. [253]) [206], [207], [208], [209], [210], [211], [212] [206], [207], [208], [209], [210], [211], [212], [214], [215], [216], [217], [218], [219], [220], [221], [222], [226], [314], [315], [316], [317], [318], [319], [320], [321], [322], [323], [324], [325], [326], [327], [328], [329], STK6 [230], [330], and WARS/Tryptophanyl-tRNA synthetise [331]. Figure 11 shows a heat map of discussed gene probes annotated with functions on cell proliferation.

The references provided next to each gene help to related these upregulated genes in the context of current research in melanoma or with the M phase of mitotic cell cycle, showing a high degree of correlation between our results and with published literature.

### Prostate Cancer - True et al.*'s* dataset (File S3)

Another microarray dataset we have selected to evaluate for the relevance of transitions of *Normalized Shannon Entropy* and *Statistical Complexity* was contributed by True et al. [332] in 2006.

The original goal of True et al. was to identify a molecular correlate for Gleason patterns 3 and, if possible, the clinically most worrisome patterns 4 and 5. They partially succeeded by linking the expression of only 86 genes with Gleason pattern 3 [332] using a standard statistical analysis. In this study, we eliminated sample 02-209C since data was acquired using a different platform and would not be useful for our analysis. The remaining thirty one (31) samples were assayed with the GPL3834 (FHCRC Human Prostate PEDB cDNA Array v4) platform using 15,488 probes. We also eliminated all the probes with missing values, remaining 13,188 probes.

**Figure 12:**
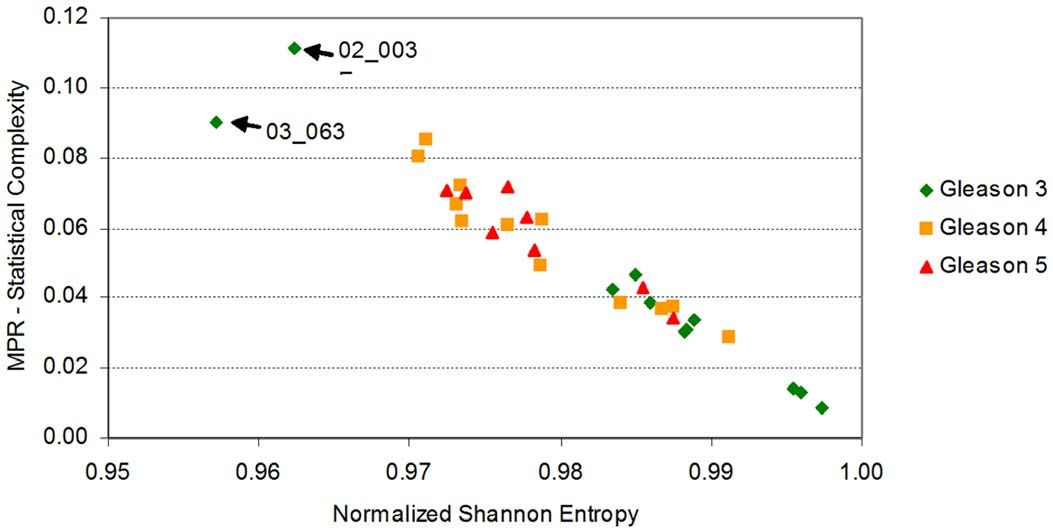
Scatter plot of the samples in the prostate cancer dataset contributed by True et al., presenting the *MPR-Statistical Complexity* of each sample as a function of its *Normalized Shannon Entropy*. The dataset contains the expression of 13,188 probes and 31 samples. The samples include 11 samples labelled ‘Gleason 3’ (in green), 12 ‘Gleason 4’ samples, and 8 ‘Gleason 5’ (in red). Two samples seem to be outliers to a generic trend, which is somewhat expected. We do expect samples with a ‘Gleason 3’ label to have higher values of *Normalized Shannon Entropy*. This is indeed the case, no sample with a ‘Gleason 3’ label has a value of *Normalized Shannon Entropy* lower than 0.985, while 14 samples corresponding to samples which are either ‘Gleason 4’ or ‘Gleason 5’ have values smaller than that threshold. In agreement with some of the caveats discussed by True et al., there exist a group of samples that, irrespective of their label, have similar values of *Normalized Shannon Entropy* (near 0.992). Samples 02_003E and 03_063 seem to be outliers to this trend, and in the case of 03_063 the sample is not even close to a hypothetical linear fit which seems to be the norm for all the samples. Figure 13 will provide further evidence that may indicate that these two samples are outliers or not to the overall trend.

We have first plotted the samples on the (*Normalized Shannon Entropy*, *MPR-Statistical Complexity*) plane (Figure 12). It was interesting to observe that there exists a high correlation between the two measures. Samples that are entirely composed of Gleason pattern 3 tend to have a greater value of *Normalized Shannon Entropy* than 0.985. We can also identify a cluster of samples that present Gleason patterns which are either 4 or 5. Note that there seems to be two outliers (02_003E and 03_063) to the generic trend of the other 29 samples. The two outliers are samples that correspond to samples labelled as having Gleason 3 patterns and both have unusually low values of *Normalized Shannon Entropy* that are well below the values of the rest of the group.

**Figure 13:**
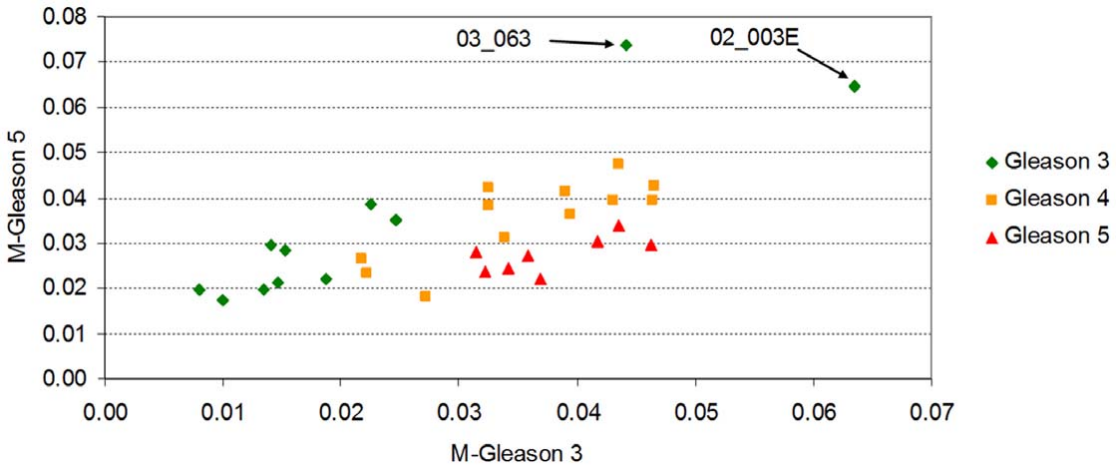
Scatter plot of the samples in the prostate cancer dataset contributed by True et al. We have used the same color coding convention we have used in Figure 12. We plot the values of two modified statistical complexities, which we will call *M-Gleason 3* and *M-Gleason 5*. Instead of using the equiprobable distribution as our probability distribution of reference (for the computation of the *Jensen-Shannon Divergence* of the gene expression profile to this distribution), as required for the *MPR-Statistical Complexity* calculation, we used a different one. For the *M-Gleason 3*, the probability distribution of the reference is obtained averaging all the probability distributions of the samples that have been labelled as Gleason 3 (analogously, we calculated *M-Gleason 5*). This is analogous to our approach in melanoma (Figure 5) in which we used normal and metastatic samples as reference sets for a modified statistical complexity. We observe that, even in this case, 02_003E and 03_063 continue to appear as outliers. In addition to the evidence, we have observed that the deletion of these two samples did not significantly alter the identification of biomarkers.

This raised a suspicion about the true nature of this phenomenon. If the labelling is correct, this may indicate a subsampled group of prostate cancer that has Gleason 3 pattern characteristics but very low entropy. Alternatively, it may indicate an experimental bias for reasons we can not explain with the available clinical information. In order to clarify the situation, and see if we can declare these two samples as outliers of the other group, we performed another experiment. We have now computed two modified complexities, which we will call *M-Gleason 3* and *M-Gleason 5* (Figure 13). The names are probably self-explanatory, but a brief reminder follows. To calculate the *MPR-Complexity*, by definition, we have used the equiprobable distribution as our probability distribution of reference (for the computation of the *Jensen-Shannon Divergence* of the gene expression profile to this distribution). In the case of the *M-Gleason 3*, the probability distribution of the reference is obtained averaging all the probability distributions of the samples that have been labelled as Gleason 3 (analogously, we calculated *M-Gleason 5*). Samples that have Gleason pattern 3 and 5 appear as separate clusters in the (*M-Gleason 3*, *M-Gleason 5*) plane with the two putative outliers of the general trend far apart (even if they have been used to calculate the average probability distribution function of the Gleason 3 pattern). Even samples with Gleason 4 pattern are located closer to samples of Gleason patterns 3, and 5, indicating that, perhaps, there exists a subsampled subtype of prostate cancer or there might be another experimental bias or factor that at present we can not resolve with the information we have for these samples. Consequently, we have decided to eliminate both samples (02-003E and PNA_03-063A) from further calculations. With these considerations, we now have a dataset with 13,188 probes and 29 samples as our dataset for further analysis.

**Figure 14:**
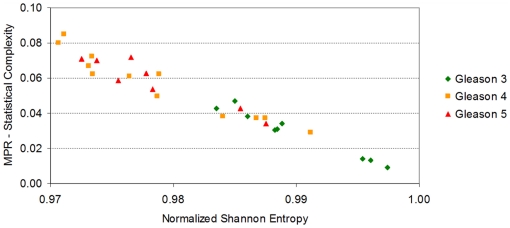
A region of interest of **Figure 12** containing the 29 samples to be used in the analysis. Due to the characteristics of this microarray dataset and the experiment setting, the *Normalized Shannon Entropy* correlates well with the established clinical notions of malignancy (high Gleason patterns). Most Gleason pattern 5 samples (in red) have lower values of *Normalized Shannon Entropy* than Gleason pattern 3 samples.

Figure 14 shows the distribution of the samples using the *Normalized Shannon Entropy* and the *MPR-complexity*. By definition, the positions of the 29 samples in the plane do not change (this figure is basically “zooming in” one region of Figure 12 that contains these samples). We note again, however, that the 29 samples seem to be separating in three different clusters. Whether we can argue about the existence or not of these gaps in *Normalized Shannon Entropy*, it is clear that there seems to be a progression as we have seen with Lapointe et al*'s* dataset. There is a group of three samples with Gleason pattern 3 that seem to have the the largest *Normalized Shannon Entropy* values. There is also a cluster that only contains samples of either Gleason pattern 4 and 5, all with *Normalized Shannon Entropy* values smaller than 0.985.

**Figure 15:**
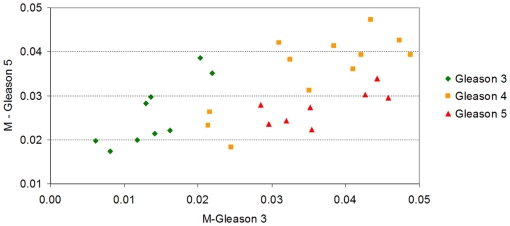
A plot showing that restricting our analysis to 29 samples does not have a major negative impact or changes in the computation of modified statistical complexities.

There is also very little variation (see Figure 15) of the positions of the 29 samples on the (*M-Gleason 3*, *M-Gleason 5*)-plane, indicating a degree of robustness that the computation of these modified complexities have, even in the presence of some outliers.

### Correlations of the genes' expressions profiles across samples with the transitions of Entropy

**Figure 16:**
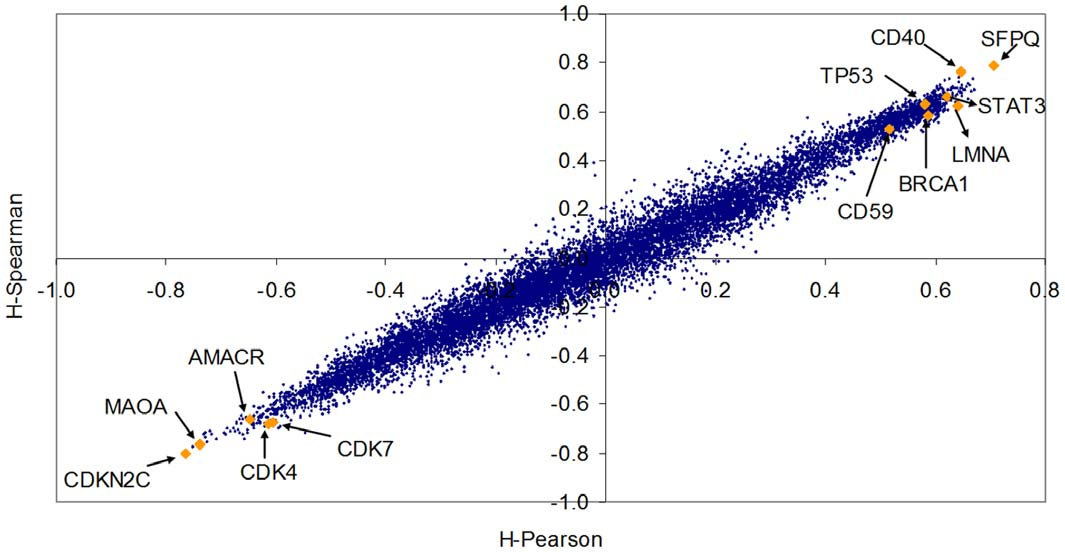
A scatter plot of Spearman versus Pearson correlation values of the probe expression of 13,188 probes in True et al.*'*s prostate cancer dataset with the *Normalized Shannon Entropy* values of the samples. The identification of probes that best correlate, either positively or negatively, with the values of the *Normalized Shannon Entropy* of the samples highlights some of the most important biomarkers in prostate cancer, like CDKN2C, MAOA, CDK4, CDK7, AMACR, TP53 and BRCA1 (with an upregualtion trend from their normal expression values). The list includes others that present a downregulation from their normal values, like LMNA, CD40, and SFPQ. These genes are discussed in detail in the context of current prostate cancer research in the main text. This result has revealed some of the most relevant biomarkers of prostate cancer progression (AMACR, MAOA, CDK4, TP53, BRCA1, STAT3), and some unexpected new complementary biomarkers (i.e. SFPQ, CD40, STAT3, LMNA, CD59 etc).

After observing that Figure 14 shows a correlation of Gleason pattern score with *Normalized Shannon Entropy*, we asked ourselves: ‘*which are the genes that most positively and negatively correlate with the transitions of Normalized Shannon Entropy?*’ We have plotted Spearman versus Pearson correlation values of probe expressions to attempt to find those that best correlate, either positively or negatively, with the *Normalized Shannon Entropy* values of the samples. The results have revealed some of the most relevant biomarkers of progression, and some unexpected newcomers. Figure 16 shows the Pearson and Spearman correlations of all the 13,188 probes in the dataset with the *Normalized Shannon Entropy* values of the samples. We have highlighted some particular genes that are discussed below.

#### *CDKN2C (cyclin-dependent kinase inhibitor 2C (p18, inhibits CDK4)*

When we compute the correlations of the probes expressions with the *Normalized Shannon Entropy* values of the samples, the gene that has the most negative correlations is CDKN2C (cyclin-dependent kinase inhibitor 2C - p18, inhibits CDK4 - NM_078626), which has been previously associated with the transition from prostatic intraepithelial neoplasia (PIN) to prostate cancer [68] (Spearman correlations with the *Normalized Shannon Entropy* range between −0.8010 and −0.7276 for all the probes for NM_078626 in this array). It has been recently argued that CDKN2C and PTEN partner in tumor suppression by constraining a positive regulatory loop between cell growth and cell cycle control pathways. Bai et al. reported that a “*double mutant mice develop a wider spectrum of tumors, including prostate cancer in the anterior and dorsolateral lobes, with nearly complete penetrance and at an accelerated rate*” [333]. Using the cancer cell lines LNCaP, PC3, PC3M, PC3M-Pro4, and PC3M-LN4 and three immortalized prostate epithelial cell lines Wang et al. report hypermethylation of CDKN2C [334].

#### *MAOA, monoamine oxidase A*

Four probes for MAOA (Monoamine oxidase type A), two for NM_000240 and two for BC008064, follow closely with CDKN2C (Spearman correlations with *Normalized Shannon Entropy* ranging between −0.7650 and −0.7202 echoing the interest of True et al. and other researchers on MAOA [332], [335], [336], [337]). Zhao et al. have recently reported that “*MAO-A is also expressed in the basal epithelial cells of normal prostate glands. Using cultured primary prostatic epithelial cells as a model, we showed that MAO-A prevents basal epithelial cells from differentiating into secretory cells. Under differentiation-promoting conditions, clorgyline, an irreversible MAO-A inhibitor, induced secretory cell-like morphology and repressed expression of cytokeratin 14, a basal cell marker*”. They also observed mRNA and protein expression of AR, the androgen receptor [338]. Peehl et al. now report correlation of MAOA expression with the dedifferentiation process, with preoperative PSA levels and the percent of Gleason 4 and 5 cancers [338].

#### *AMACR, Cyclin G2, CDK4 and CDK7*

Other probes that also have high negative correlations with the *Shannon Normalized Entropy* correspond to CCNG2 (Cyclin G2) CR598707, CDK4 (Cyclin-dependent kinase 4), CDK7 (Cyclin-dependent kinase 7, TFIIH basal transcription factor complex kinase subunit) [339], and AMACR (Alpha-methylacyl-CoA racemase), an “*obscure metabolic enzyme (that has taken) centre stage*” [340] as judged by the extraordinary convergence to this biomarker in prostate. We believe that our result is an important finding. AMACR was not judged of importance according to the methodology used in [332] and it was barely cited in that manuscript. Here we present results, from an unifying biological and informational principle, which allows (using Ref. [332]'s own data) the identification of the most central current biomarker with a truly compelling body of support in independent studies [316], [340], [341], [342], [343], [344], [345], [346], [347], [348], [349], [350], [351], [352], [353], [354], [355], [356], [357], [358], [359], [360], [361], [362], [363], [364], [365], [366], [367], [368], [369], [370], [371], [372], [373], [374], [375], [376], [377], [378], [379], [380], [381], [382], [383], [384], [385], [386], [387], [388], [389], [390], [391], [392], [393], [394], [395], [396], [397], [398], [399], [400], [401], [402], [403], [404], [405], [406], [407], [408], [409], [410], [411], [412], [413], [414], [415], [416], [417], [418], [419], [420], [421], [422], [423], [424], [425], [426], [427], [428], [429], [430], [431], [432], [433], [434], [435], [436], [437], [438], [439], [440], [441], [442], [443], [444], [445], [446], [447], [448], [449], [450], [451], [452], [453], [454] that currently exists in prostate cancer.

#### *TP53 and BRCA1*

There exist several studies linking two “tumor suppressors” BRCA1 and TP53, its expression, status and mutations, to prostate cancer progression [51], [55], [455], [456], [457], [458], [459], [460], [461], [462], [463], [464], [465], [466], [467], [468], [469], [470], [471], [472], [473], [474], [475], [476], [477], [478], [479], [480], [481], [482], [483], [484], [485]. BRCA1 is one coregulator of AR, the androgen receptor [486], [487], [488], [489] and inhibits ESR1 (Estrogen receptor alpha) activity [490], [491]. Knockdown of BRCA1 results in the accumulation of multinucleated cells, indicating that BRCA1 regulates gene expression of an orderly progression during mitosis [492], preserving chromosomal stability [490]. BRCA1 showed decreased expression in a study involving immortalized prostate epithelial cells before and after their conversion to tumorigenicity [493]. Lack of BRCA1 function may impair activation of STAT3 [494]. Inactivation of TP53 by somatic mutations is also associated to the panel of disruptions which are common for this “tumor suppressor” [113]. One possible mechanism for gene silencing is CpG island methylation. Rabiau et al.show in [495] that BRCA1, RASSF1, GSTP1 and EPHB2 promoter methylation is common in prostate biopsy samples. Mannicia et al. suggest that the mitochondrial localization of BRCA1 proteins may be a significant factor in regulating the mitochondrial DNA damage [5].

#### *SFPQ - (Polypyrimidine tract-binding protein-associated splicing factor)*

The most positively correlated gene with the loss of *Normalized Shannon Entropy* is SFPQ/PSF (Polypyrimidine tract-binding protein-associated splicing factor) (Spearman correlation of 0.7902), a multifaceted nuclear factor [496], [497] which is also a putative regulator of growth factor-stimulated gene expression [498]. This is extremely interesting as it has been recently shown that the AR/PSF complex interacts with human PSA gene and that PSF inhibits AR transcriptional activity [499]. The loss of expression of SFPQ and other proteins that together regulate androgen receptor-mediated gene transcription [500] (see also [501], [502]) may indicate they have a role not only as a biomarker of the progression and well as transitions of the disease to androgen independence. In a study of human labor, Dong et al., also showed that SFPQ acts as a Progesterone Receptor corepressor, thus putatively contributing to the functional withdrawal of progesterone [503]. We will return to this particular gene later on the ‘Discussion’ section as new evidence of its role in nuclear organization has been documented.

#### *CD40 - (TNFRSF5, B-cell surface antigen CD40)*

The loss of *Normalized Shannon Entropy* gives us several markers that indicate a de-differentiation from a epithelial basal phenotype and an increasing loss of control of cell cycle regulation (due to uncoordinated upregulation of CDK4, CDK7, CCNG2 with their functional partners). This poses the question: *What can we observe while looking at the genes that most positively correlate with the loss of Normalized Shannon Entropy?* We observe, second on the ranking of all samples, a probe for CD40 (TNFRSF5, B-cell surface antigen CD40), BX381481 with a Spearman correlation of 0.7616. Loss of CD40 expression has been previously reported in prostate cancer and it is the object of a study that attempts to establish dendritic cell gene therapies [504], [505], [506], [507], [508], [509], [510], [511], [512], [513], [514], [515], [516], [517], [518], [519], [520], [521], [522]. We will continue discussing CD40 in the following subsection in concert with other genes.

### Correlations of the genes' expressions profiles across samples with the MPR-Statistical Complexity

Another natural question can be asked: *Which is the extra information that we can obtain the by analysing the correlations with the MPR-Statistical Complexity in this case?* As we have discussed before, and can be appreciated from Figure 14, there is a strong correlation between the *MPR-Statistical Complexity* and the value of the *Normalized Shannon Entropy*. It appears in prostate cancer, as in this gene expression dataset, the *reduction* of *Entropy* is not the major factor responsible for the *increase* in *MPR-Statistical Complexity*. Again, it is perhaps better to now look at one of the multiplicative factors of the statistical complexity measure, the *Jensen-Shannon divergence* to the equiprobability distribution, as this is increasing the *MPR-complexity*.

#### *CD40*

We present more evidence of the case of CD40 as a biomarker, since a probe for CD40 (BX381481) ranks 6^th^ (the Spearman correlation of the probe expression with the *Jensen-Shannon divergence* from the equiprobability distribution is −0.5764). CD40 is a member of the TNF receptor superfamily. Notably, in 56 out of 57 archival prostate cancer samples Palmer et al. have reported no CD40 expression [518]. However, CD40 expression was present in normal prostatic acini, so they proposed that “*invasive prostate cancer is a CD40-negative tumour*” (see the previous results of Moghaddami *et. al.* [514]). Matching our observations, they proposed that CD40 provides “*insight into progression of cancer from normal epithelium*”; our proposed methodology is revealing this fact as well. Depletion of CD40 in the tumour microenvironment may be central in avoiding the action of the immune system [506], as prostate cancer induces a progressive suppression of the dendritic cell system [520]. It is perhaps a central piece which should be put together in the context of other pieces of information coming from immunotherapy [508], [512], [513], [516] and pharmacological studies [507] that warrant serious investigation towards the design of new and improved clinical studies [508], [517].

#### *CD59 molecule, complement regulatory protein*

Four probes for protectin [335], [523], [524], CD59, with Spearman correlations with the *Jensen-Shannon divergence* from the equiprobable distribution, ranging from −0.61823 to −0.5089, rank between the 1^st^ and 39^th^ position (when we rank genes according to this correlation in ascending order). CD59 is an interesting gene as “*a comprehensive investigation of CD59 expression in prostate cancer has not been conducted yet*” [524]. Like LMNA (which is ranked third and will be discussed later) the rank of CD59/protectin means that these genes progressively loose expression of these probes. CD59 is expressed in the prostatic epithelium [525] and in prostasomes [526]; secretory granules which are produced, stored and released by the glandular epithelial cells of the prostate [527]. Babiker et al. concluded in [335] that prostasomes (via expression CD59) contribute to the protection of malignant cells from complement attack. We now investigate if the ratio of delta-catenin to CD59 can is a more robust biomarker for non-invasive prostate cancer detection, particularly after the results presented in [528]. We also note that CD59 may be also relevant to reveal the heterogeneous nature of prostate cancer. Its correlation was good, but is not lower than −0.62, which in our experience, indicates that we may be dealing with at least two types tumors in this dataset. Indeed, Xu et al. obtained CD59 mRNA levels were determined by real-time PCR in matched (tumor/normal) microdissected tissues from 26 cases and they found that: “*High rates of CD59 expression were noted in 36% of prostate cancer cases and were significantly associated with tumor pT stage (P = 0.043), Gleason grade (P = 0.013) and earlier biochemical (PSA) relapse in Kaplan-Meier analysis (P = 0.0013). On RNA level, we found an upregulation in 19.2% (five cases), although the general rate of CD59 transcript was significantly lower in tumor tissue (P = 0.03)*” [524]. *They concluded that: “CD59 protein is strongly expressed in 36% of adenocarcinomas of the prostate and and is associated with disease progression and adverse patient prognosis”* [524]. Jarvis et al. have previously hypothesized that CD59 expression, in some cancer cells, may help to regulate the immunological response, protecting them from the cytolytic activity of complement [523] (see also [529], [530]).

#### *LMNA (Lamin A/C)*

The third probe in the ranking corresponds to a LMNA (Lamin A/C), AY528714. Mutations on LMNA have been linked at 10 different human diseases [531], [532]. LMNA, due to its functions, could be involved in important cell fate decisions as lamins are involved in the organization of the functional state (and position) of interphase chromosome [531]. Lamins are “scaffolders” for the function of nuclear processes such as chromatin organization, DNA replication, cellular integrity and transcription [532]. As a consequence Lamins are involved in several clinical syndromes [533], [534], [535]. Among the recent functions attributed to LMNA is as an intrinsic modulator of ageing within adult stem cells via a mechanism where LMNA act as signalling receptors in the nucleus. These observations correspond to Pekovic and Hutchinson who observed that dysfunction of LMNA leads to inappropriate activation of self-renewal pathways and initiation of stress-induced senescense [536]. In lmna-deficient mouse embryonic fibroblasts (lmna(−/−) MEFs), the loss of lmna“*dramatically affects the micromechanical properties of the cytoplasm*”, since “*Both the elasticity (stretchiness) and the viscosity (propensity of a material to flow) of the cytoplasm in Lmna(−/−) MEFs are significantly reduced*” [537]. Using ballistic intracellular nanorheology to evaluate the micromechanical properties of the cytoplasm of these cells, Lee et al. conclude: “*Together these results show that both the mechanical properties of the cytoskeleton and cytoskeleton-based processes, including cell motility, coupled MTOC and nucleus dynamics, and cell polarization, depend critically on the integrity of the nuclear lamina, which suggest the existence of a functional mechanical connection between the nucleus and the cytoskeleton. These results also suggest that cell polarization during cell migration requires tight mechanical coupling between MTOC and nucleus, which is mediated by lamin A/C*” [537] (see also [538], [539]). In addition to these very interesting findings, a functional association of LMNA and the retinoblastoma protein (pRB) exists. Nitta et al. have shown that pRB needs to be stabilized by LMNA for INK4A-mediated cell cycle arrest and that somatic mutations in LMNA may also have a role in tumor progression [540]. In mammalian cells, LMNA a) colocalizes with c-FOS at the nuclear envelope, b) suppresses AP-1 through a direct interaction with c-FOS and, in LMNA-null cells perinuclear localization of c-FOS is absent (but it is restored when it is overexpressed, c) LMNA-null cells have enhanced proliferation [74]. These results obtained by Ivorra et al. are giving the indication that of yet another mechanism of cell cycle and transcriptional control mediated by LMNA [74] (see also [541]). LMNA has also been proposed as an inhibitor of adipocyte differentiation [542]. Hutchingson et al. have proposed the alias of “guardian of the soma” for lamins A and C as they seem to have “*essential functions in protecting cells from physical damage, as well as in maintaining the function of transcription factors required for the differentiation of adult stem cells*” [543].

### NF-kappaB regulated genes reveal links to focal adhesion and ECM-receptor interaction and immune response disregulation

From our results, we can not completely establish if the downregulation of CD40 and CD59 are enough to pinpoint an impaired or abnormal immune response. If we continue the inspection of the list, the first 20 probes give us more supporting evidence. The 20 probes correspond to 13 different genes. Five of these 13 genes have Genome Ontology information annotated as “defense response”, the above mentioned CD59 and CD40 as well as IL4R (interleukin 4 receptor, CR616481), XBP1 (X-box binding protein 1, AK093842) and HLA-A (major histocompatibility complex class I HLA-A29.1, BU075230). Takahashi et al. [544] report an inverse correlation between XBP1 expression and histological differentiation in a series of prostate cancers without hormonal therapy, the expression of XBP1 was localized in epithelial and adenocarcinoma cells of the prostate and the majority of refractory cancer cases exhibited weak XBP1 expression), MST1/STK4 (along with MST2/STK3) act as inhibitors of endogenous AKT1, a mediator of cell growth and survival [545].

We can not yet know what reason is behind their joint downregulation, but another interesting common denominator is that 12 out of 13 genes share a regulatory motif for NF-kappaB (according to TRANSFAC, V$NFKB_Q6_01). A putative role for NF-kappaB in prostate cancer has been reported based on the observation of the centrality of NFKB on two up- and down-regulated networks compairing prostate tumors and healthy tissue [546] and in a larger study by McDonnel et al. [547] (255 core prostate cancer tissue microarrays from 47 prostatectomy specimens). Several other researchers are currently investigating different roles of the NFKB family in prostate cancer [548], [549], [550], [551], [552], [553], [554], [555] and it could be a promising target for intervention [555], [556], [557], [558], [559], [560], [561], [562], [563], [564], [565], [566], [567], [568], [569], [570], [571]. If we include other genes following the ranking order, the first 38 genes in the ranking include 33 that have the regulatory motif V$NFKB_Q6_01 (GATHER reports for this list a p-value of 0.0006). Even when we double the list to the probes that correspond to the first 76 different genes recognized by GATHER, 58 of them have the regulatory motif V$NFKB_Q6_01, with *p-value = 0.003* (ATP6AP2, BCAT1, BTG2 [572], [573], [574], [575], [576], [577], [578], C14orf123, C18orf45, CCL2, CD302, CD40 (already discussed), CD59 (already discussed), CHI3L1, COL16A1, COMMD6, CRABP2, CSRP1, CTBP2, CTGF (Connective tissue growth factor, [579], [580], [581], [582]), DES, DMN, DNAJB1, EGF, EMP1, FHL2 [583], [584], [585], [586], [587], [588], GRIPAP1, GSTM1 [589], [590], HBEGF, IL4R, ITGA3, ITGA7, JUNB [591], [592], KIAA0152, KIAA1191, KIAA1324, KLF6, LAMB2, LMNA (already discussed), NFATC1, NFKB2, NUDC [593], P4HB, PDK2, PIM1, PISD, PXN, RAP1B, RNF40, SARA1, SEC61A1, SGTA [594], SLC12A2, SRD5A2, STAT6 [595], [596], TACSTD2, TBX1, TMED3, VPS39, WDFY3, XBP1 [544], ZAK). This result indicates that our results support the importance of NFkappa-B and the huge amount of research effort to understand the role of the NFkappa-B activity and its potential as a target for intervention in prostate cancer (File S4).

The group of 58 biomarkers contains one of particular interest, STAT6. This gene is considered a survival factor in prostate cancer and a key regulator of the genetic transcriptional program responsible for progression [595]. STAT6 has been recently linked to HPN as one of the most robust pair of biomarkers for prostate cancer using an integrative approach that linked several microarray datasets [596].

### Focal and cell adhesion modifications can be inferred by monitoring losses of a group of genes composed by EFG, Integrins, LAMB2, Paxillin and RAP1B

Analysis using GATHER of this group reveals that six of these 58 genes are in KEGG pathway *path:hsa04510*, *Focal adhesion* (EGF, ITGA3, ITGA7, LAMB2, PXN, RAP1B, p-value<0.0007) and from these there are three in *pathway:hsa045122*, *ECM-receptor interaction* (ITGA3, ITGA7, LAMB2, p-value<0.005) while four of these six are also in *path:hsa04810: Regulation of actin cytoskeleton*, (EGF, ITGA3, ITGA7, PXN, *p*-value<0.01).

#### *LAMB2*

Alterations of the gene profile of LAMB2 and CDKN2C/p18(Ink4c), a CDK4 inhibitor, have been reported on the transition from prostatic intraepithelial neoplasia (PIN) to prostate cancer [597] (see also [333]).

#### *ITGA7 (integrin, alpha 7) and ITGA3 (integrin, alpha 3)*

The contribution of the loss of these integrins and the subsequent derived impairment on cell adhesion has been reported in several tumours. Ren et al. in [598] report that “*Focal or no integrin alpha 7 eexpression in human prostate cancer and soft tissue leiomyosarcoma was associated with a reduction of metastasis-free survival (for example, for prostate cancer with focal or no expression, 5-year metastasis-free survival was 32%, 95% CI = 24.4% to 40.3%, and for prostate cancer with at least weak expression, it was 85%, 95% CI = 79% to 91%; p-value<.001)*”.

## Discussion

*“Any method involving the notion of entropy, the very existence of which depends on the second law of thermodynamics, will doubtless seem to many far-fetched, and may repel beginners as obscure and difficult of comprehension.”*

Willard Gibbs, *Graphical Methods in the Thermodynamics of Fluids,* (1873)

### Transcriptional vs. Karyotypic Entropy

The changes of the *Normalized Shannon Entropy* and *Statistical Complexity* of the gene expression profile of a cancer cell are associated with the gradual deterioration of genome transcriptional information content due to the modification of its structural and functional integrity during disease progression. Our results clearly suggest that we can track the cancer cell's progression by following observable changes in the *Shannon Entropy* and, in particular, by employing the *Jensen-Shannon Divergence* of the gene expression profile of a sample to the normal expression profile. We have also shown if an average expression profile of some state of interest can be properly defined (i.e. distant metastasis) then the *Jensen-Shannon Divergence* can help us to identify which probes best correlate with these measures resulting in useful biomarkers.

Before any thermodynamical consideration could be discussed, we note that there is a clear and objective *informational* perspective that our study delivers. In this study we have chosen to position ourselves as the ‘receivers’ of a ‘transcriptional message’. In this experimental perspective the tumor tissue is the ‘sender’ (the source of information) and the *high-throughput technology* (gene expression microarrays in this case) can be regarded as the transmission medium (providing noise and distortion). As we explain in the ‘Materials and Methods’ section, the *Shannon Entropy* of a gene expression profile is the *average expected surprisal* of that profile understood as a message. The *Normalized Shannon Entropy* makes this surprisal an *intensive* measure and the correlation of the gene expression patterns across samples with this measure can deliver useful biomarkers to track the progression of transcriptional change. After normalization, we have a measure that does not depend of the number of probes of the *high-throughput technology*, although, it obviously does depend on the type of probes used.

We believe that the readers may have already noticed an apparent paradox. While some researchers understand cancer progression as a mechanism that *increases* entropy, we actually observe a reduction of *Normalized Shannon Entropy* in this work. This means that our *normalized average expected surprisal*, as receivers of the transcriptional message, is smaller. We must then discuss the *physical* meaning of thermodynamic entropy, its current use in systems biology and cancer research genetics and the informational measure we use in this paper to clarify these notions in this context.

In biomedical research there exists a certain consensus among cancer researchers that *genetic instability* or “*mutability*” is a major critical force of cancer progression, but it is not the only one to consider. It is clear that the mutational damage of key genes (like TP53, TERT, BRCA1, RB1, etc.), and the collective damage inflicted on key DNA repair mechanisms (like Nucleotide-excision repair and Base-excision repair) collaborate for an increasing acceleration of the number of genomic changes. Sub-microscopic alterations of the genome accumulate in cancer progression in an irreversible way and “*are compounded by the widespread scrambling of the chromosome structure, and thus the karyotype, found in cells from the great majority of solid tumours*” [599]. In Weinberg's own words [599]: “*we learned that this chromosomal chaos also contributes this progression forward*”.

This “*chromosomal chaos*” [600] or “*cancer as a chromosomal disease*” perspective is viewed by some researchers not as just a side consequence of mutational damage, but as the main core theme to understand a number of unexplained issues in cancer progression. “*In sum, cancer is caused by chromosomal disorganization, which increases karyotypic entropy*” [601]. Regarding the cancer types studied in this paper, one particular “measure of disorder of a system”, *aneuploidy*, has been observed in poorly-differentiated prostate cancer cells and it is often associated with a more agreessive phenotype [602], [603], increased PSA levels [604], [605], and correlate with Gleason score [606], [607], [608]. *Gene fusions* and *chromosomal rearrangements* are other source of increase in the “disorder” of the genome organization and they are increasingly being recognized as a major player in prostate cancer progression [609]. The increase in “*karyotypic complexity*” and “*extended aneuploidy and heteroploidy*” may be already enough to develop a malignant melanoma phenotype, as the report of Gagos et al. indicate [610]. The observed finding of aneuploidy in melanoma (also including uveal melanoma) is also increasingly important due to a number of different independent observations [247], [611], [612], [613], [614], [615], [616], [617], [618]. It is in this context that the word ‘*entropy*’ has been used.

The magnitude of the “chromosomal chaos” is also evident from comparative genomic hybridization (CGH) studies which show significant variations in the copy number of individual chromosomal segments. ‘*Chaos*’ is really a very appropriate word to describe what we observe from CGH data. The genomic changes are not distributed uniformly at random. ‘Chaos’ has been described by some researchers as “*a kind of order without any periodicity*”. Some common changes seem to consistently appear in several independently arising tumours of the same type, and sometimes the researchers suggest common links [619]. Our work has addressed, in part, this question: “*Can we quantify the chaos observed in the genome from the increasingly available transcriptional data and relate it to tumour progression?*” If no commonalities were observed, we would not have found interesting biomarkers that seem that strongly correlate with the divergences from normal tissue types. We know from our results that these commonalities do occur.

We need to go back to basics to explain these evolving concepts and resolve this apparent paradox. The phrase “*karyotypic entropy*” has been used in the past to define what is actually a *divergence* from the normal chromosome structure and it genomic organization. This denomination has also been employed by several authors, notably [601], but it has also been used in at least two other publications [620], [621]. These works have in common the use of this term to refer to a “*disorder*”, fuelled by the undergraduate textbooks indoctrination of associating increase of entropy in natural spontaneous processes with the increase of “observed disorder” in the system. We propose that the use of a natural measure of divergence, the *Jensen-Shannon divergence*, could not only be a more formal, but also more appropriate modelling approach. As such, we propose to introduce the term ‘*karyotypic divergence*’ or ‘*karyotypic Jensen-Shannon divergence*’ to replace this concept and to avoid a subjective approach.

*Why is it the case that we observe the* Normalized Shannon Entropy *of the transcriptional profile decreasing with cancer progression when intuitively our average expected surprisal (Shannon Entropy) should increase with progression?*

Arieh Ben-Naim in his recent book “*A farewell to Entropy: Statistical Thermodynamics based on Information*” [622] comments:“*It is interesting to note that Landsberg (1978) not only contended that disorder is an ill-defined concept, but actually made the assertion that ‘it is reasonable to expect* ‘disorder’ *to be an intensive variable’*”. Ben-Naim also states: “*In my view, it does not make any difference if you refer to* information *or to* disorder, *as subjective or objective. What matters is that order and disorder are not well-defined scientific concepts. On the other hand, information is a well-defined scientific quantity, as much as a point or a line are scientific in geometry, or mass or charge of a particle are scientific in physics.*” However, in a manuscript entitled “*Can Entropy and ‘order’ increase together ?*” Landberg defines (in an attempt to decouple the notions of order and entropy), for a thermodynamical system that can be on *N* states the ‘disorder’ *D(N)* to be the *Normalized Entropy* (which is a function of *N*) divided by Boltzmann's constant [623]. ‘*Disorder*’ then is an intensive magnitude bounded by 0 and 1, and ‘order’ is defined as *1-D(N)*.

While Landberg's decoupling argument between order and entropy [623] may still be controversial in Physics, the question is pertinent for our apparent paradox (the question that motivates this subsection). Borrowing from the title of his paper we could now state the central question as “*Can Shannon Entropy increase while the Normalized Shannon Entropy decrease?*” The solution of this apparent paradox is a trick of escapologism, perhaps also paralleled by what a cancer cell may be experiencing (or “reacting” in response to increased sources of stresses), and it is worth discussing in this context. Let *H[X]* be *Shannon Entropy* for an ensamble *X* with *N* different values. We will now assume, and here is the trick, that *N* is not a constant, but a function of time *N(t)*. Let *D(X(N(t)))* be the *Normalized Shannon Entropy*. By definition *D(X(N(t))) = H(X(N(t)))/* 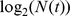. Then, just by taking the time derivatives it can be shown that the time variation of *D(X(N(t)))* can be negative, although the time rate of *H[X]* can be positive.
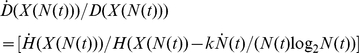
where *k* is a constant. The escape to our paradox is “achieved” via making explicit the time variability of *N(t)*. Landberg explicitly mentions that biological systems are examples where growth processes increase *N(t)*, and perhaps the increased diversity in the transcriptome of a cancer cell during progression is one of such examples.

This discussion somehow resolves the apparent disassociations due to language barriers that may exist between the different disciplines (physics, information theory, molecular biology and oncology). A biologist may regard a cancer cell as an entity that, during progression, may “spread” its transcriptomic profile, including the generation of a large number of novel molecular species (due to adquired characteristics during its “*devolution*” from the normal type). In our informational perspective, this would be analogous to a situation in which the sender of a message, after some time, decides to increase the size of the alphabet of transmitted symbols. Clearly, it is intuitive to think that the receiver would be in a situation of increased *Shannon Entropy*. However, if the receiver is not aware of the new symbols (or is not able to detect them) and some of the symbols of the previous alphabet are no longer used, the receiver would now perceive a reduction of *Normalized Shannon Entropy*, observing an increasing order.

We now borrow an illustrative example from Landberg [623], but we add a twist to this argument for the purpose of illustrating this discussion. Suppose we have a sender transmitting only two possible symbols *(N = 2)*, and we will assume that we have the same probability, let's denote this as (1/2, 1/2). Then the *average expected surprisal (Shannon Entropy)*, is *H(X) = 1*, and the *Normalized Shannon Entropy* is also equal to one. Assume now that now our sender starts to transmit using another symbol, so that we now have theoretical probabilities of (0.5, 0.25, 0.25). Then *N = 3*, and the *average expected surprisal* increases to *H(X′) = 1.5* the *Normalized Shannon Entropy* is now *1.5/* 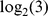 = 0.946… (a reduction). This ‘third symbol’ could actually represent a new “molecular species” or a protein isoform that would not be normally expressed in that tissue type [624], or even something entirely new, product of a mutational/deletional event. If our hypothetical high-throughput technology can only be detecting the first two symbols, and following the conventions we established in the ‘Materials and Methods’ section, we would be “observing” frequencies of (2/3,1/3) since the other events would not be detected with our equipment. As a consequence, the both the 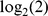 = 1, *Shannon Entropy* and the *Normalized Shannon Entropy* are both reduced to 0.918293. Obviously, we can not count what we can not observe. As a consequence, a degenerating transcriptional profile that produces novel molecular species, and at the same time reduces those which we can not measure with a particular technology, would look increasingly more ordered.

### Exporting entropy, Maxwell Demons and Aquaporins

We envision that physicists may find here a fertile ground to explore new ideas and attempt novel mathematical formalisms for cancer progression from the realm of *finite-state thermodynamics* [625] and in particular *endorevesible processes* [626] and *endoreversible thermodynamics* [627]. Some molecular alterations would then be part of the set of revesible processes that could occur in a cancer cell, while other processes like aneuploidy or gene fusions could be truly “*irreversible genetic switches*” associated with cancer progression [628]. If we assume that the process is slow (i.e. the times required for significant variations of the transcriptome's profile is large in comparison with the cell's processes time scales), and follwing the results of Spirkl and Reis [626], it may be possible that we have a constant entropy production rate exists during cancer progression leading to Hauptmann's “*entropic devolution*” [629]. Hauptmann sees a malignant tumour as “*a dissipative structure arising within the thermodynamical open system of the human body*” that starts when “*a localized surplus of energy exists and there is no possibility to export entropy. An energetic overload in most malignant cells is indicated by their abnormally high phosphorylation state.*” His perspective, preceeded in part by Dimitrov [630], Klimek [631], [632] and Marinescu and Viculetz [633] might then fit well an endoreversible thermodynamic formalism. Hauptmann says in [629] “*I believe that cancer is a special kind of adaptation to energetic overload, characterized by multiplication and mutation of genomic DNA (generation of new biomolecules which enhance the probability of survival under harmful conditions), and by chiral alterations (reduction of entropy by entrapping energy) leading to abnormal configurated biomolecules. In this regard the genetic alterations are probably secondary changes. Cancer serves to dissipate energy in a type of developmental process but one in which the results are harmful to the whole organism: an entropic devolution.*”

This thermodynamical perspective is now worth exploring and we will discuss it in this context. Assuming that a cancer cell is in a state of “*energy overload*”, without “*the possibility of exporting entropy*”, could it lead to some type of “*genetic alterations*”? Which key mechanisms might be impaired? What consequences is this “system” delivering? Could this be another hallmark for *oncosystems* indentification?

In 1871, in this book called “*Theory of Heat*”, Maxwell speculated the idea of “*a being, who can see the individual molecules*” and who has enough reactive intelligence to open and close a unique small hole existing between two communicating vessels (called ‘A’ and ‘B’). An ideal gas filled both vessels, so that starting at uniform temperature the intelligent being could observe the molecules and close and open the hole accordingly to a mission: “*to allow only the swifter molecules to pass from A to B, and only the slower ones pass from B to A.*” The being, “*without expenditure of work raise the temperature of B and lower that of A in contradiction to the second law of thermodynamics.*” The ability of the “being” to use observable information about the system to lower the thermodynamical entropy has motivated many articles in physics and fuelled the imagination of many since it was originally introduced by Mawell, and named as “demon” by Thomson three years later [622]. An excellent collection of articles until 1990 [634], [635], [636], [637], [638], [639], [640], [641], [642], [643], [644] was edited by Leff and Rex [645]. The Maxwell “*demon*”, far from being “exorcised” from Physics, still inspires interesting new perspectives [634], [635], [636], [637], [638], [639], [640], [641], [642], [643], [644], [646], [647].

In a letter to Peter Guthrie Tait, Maxwell writes about the “demons”: “*Is the production of an inequality of temperature their only occupation? No, for less intelligent demons can produce a difference in pressure as well as temperature by merely allowing all particles going in one direction while stopping all those going the other way. This reduces the demon to a valve. As such value him. Call him no more a demon but a valve like that of the hydraulic ram, suppose.*” (from [645], p. 6). Maxwell gives again here a sign of his brilliant mind, “degrading” the demon to a valve, but also offering an inspiring perspective to oncosystems research. Which types of mechanisms exist in biological systems, and particularly in individual cells, to control these differential values in key parameters? Could changes of key physical parameters for metabolic processes of the cytoplasm and cell's organelles like *temperature*, *volume*, *pH* or *electrochemical potentials* be also implicated in cancer progression?

The influence of temperature may be giving an interesting working hypothesis for further research. What are the consequences if cancer cells are a different type of open system which also operates at a different temperature than a normal cell? Butler et al. have studied p53 and they argue that at temperatures above 37 degrees centigrades wild-type p53 spontaneously loses DNA binding activity. While folding kinetics do not show important changes in a range from 5 to 35 degrees C, the unfolding rates accelerate 10,000-fold. This leads to a somewhat unexpected mechanism of p53 inactivation. It could be the case that a fraction of p53 molecules become trapped in misfolded conformations with each folding-unfolding cycle due to the increased frequency of cycling. The occurrence of misfolded p53 proteins can lead to aggregation and subsequent ubiquitination in the cell, leading to p53 inactivation [648], [649]. If a key “guardian of the genome integrity” [650], [651] and its remarkable conformational flexibility [652] is challenged by an increase of temperature [653], its role in genotoxic damage and adaptive response (like that of the skin to UVB damage [654]) may be impaired. The same may occur for other members of the DNA damage response. An increment in temperature has already been linked to skin carcinogenesis. Boukamp et al. report in that [655] “*exposure of immortal human HaCaT skin keratinocytes (possessing UV-type p53 mutations) to 40 degrees C reproducibly resulted in tumorigenic conversion and tumorigenicity was stably maintained after recultivation of the tumors.*”

On the other hand, natural gradients on physical biochemical properties can also be challenged in a cancer cell. This in turn derives in metabolic processes running under abnormal parametric circumstances. It is well-known that compartimentalization, in biological systems, naturally require the existence of mechanisms that would keep some key state variables relatively constant, or within bounds, for normal operation of the metabolic processes. One example is very illustrative and a case in point. Instead of demons, holes, or valves, the cell requires pores in its membranes to allow osmotic regulatory processes, yet it should preclude the conduction of protons. This is a nanotechnological design problem not faced by Maxwell, but certainly solved by biological systems without the need of an “intelligent being” as Mawell cleverly pointed to Tait in his letter.

This discussion brings us to one of the gene families we have already discussed in this paper, the aquaporins [184], [656], [657], [658], [659], [660], [661]. They are considered the primary water channels of cell membranes [662], [663], [664], [665]. The specific functions of each member of this family are now being slowly mapped by several research labs around the world [666]. Their clinical role in cancer [667], [668], [669], [670], [671], [672], [673], [674], [675],obesity [676], malaria [677], [678] and other diseases is emerging [657], [679], [680], [681], [682], [683], [684], [685], [686], [687], [688], [689]. In [690], our group observed the dowregulation of AQP3 in all melanoma cell lines studied of the NCI-60 dataset of Ross et al.; this dowregulation was also observed for the CNS and Renal cell lines. AQP3 was relatively upregulated for Leukaemia and Colon cell-lines (we refer the reader to the Supplementary Material of [690] for details). Inhibition of AQP3 in prostate cancer cells was already proposed as a mechanism that increases the sensitivity to cryotherapy treatment [691].

The aquaporins are not “an intelligent being” in any real sense, yet they are so formidable selective that they could easily parallel Maxwell demon's efficiency in creating the right conditions for the cell. Wu et al. give us some clues on the role of point mutations in the AQP1 and how their effective electrostatic proton barrier can be impaired [692]. The elicitation of the detailed mechanistic explanation of this extraordinary selectivity is under intense investigation with a number of techniques, including sophisticated molecular dyanamics simulations, for an overview of this field see [665], [693], [694], [695], [696], [697], [698], [699], [700], [701], [702], [703], [704], [705], [706], [707], [708]. One less known feature of aquaporins is that they may not only channel water, but also carbon dioxide and ammonia [709], [710], [711], glycerol [712] and urea and other small solutes [713] and, very relevant for cancer research, *hydrogen peroxide* [188]. At least two of members of this family have been observed in the inner mitochondrial membrane in different tissues. This in turn may indicate mitochondrial roles for aquapotins in osmotic swelling induced by apoptotic stimuli [714].

**Figure 17:**
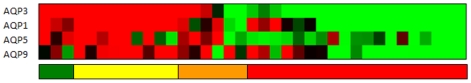
Heat map showing the expression of four of the six probes corresponding to aquaporins (AQP1, AQP3, AQP5, and AQP9) in Haqq et al.'s melanoma dataset. Primary melanaoma samples (annotated in green) and benign nevi (in yellow) show higher expression values. Primar melanoma (in orange) show a mixed behaviour and metastaic melanoma samples (in red) show in comparision that their expression is remarkably lower. We highlight the similarity of this finding with Figure 8, in which we have shown the same behaviour for a group of genes functionally annotated as being involved in *cell adhesion*, *cell-cell communication*, *tight junction mechanisms* and *epithelial cell polarity*. Metastatic melanoma samples, in comparison, show remarkably reduced values of the joint expression of these four probes, indicating the possibility of an impaired function of these highly selective mechanisms.

Could it be possible that we can track cancer progression by looking at some of these “*Maxwell demons*”? We have seen in Figure 10, that AQP3 has a reduced expression with increased progression in our melanoma dataset. Cao et al., reported that ultraviolet radiation induced AQP3 down-regulation in human karatinocytes; thus AQP3 has become a strong and plausible link between UV radiation, skin dehydration [186], [715] and photoaging [189]. This may indicate an impared function on skin hydration [184], [185], [716], [717], [718], [719]. The expression of AQP3, as well as AQP1, AQP5, and AQP9 seem to be correlated with melanoma progression, indicating a common pattern of downregulation from the higher values in normal skin and benign nevi (see Figure 17).

**Figure 18:**
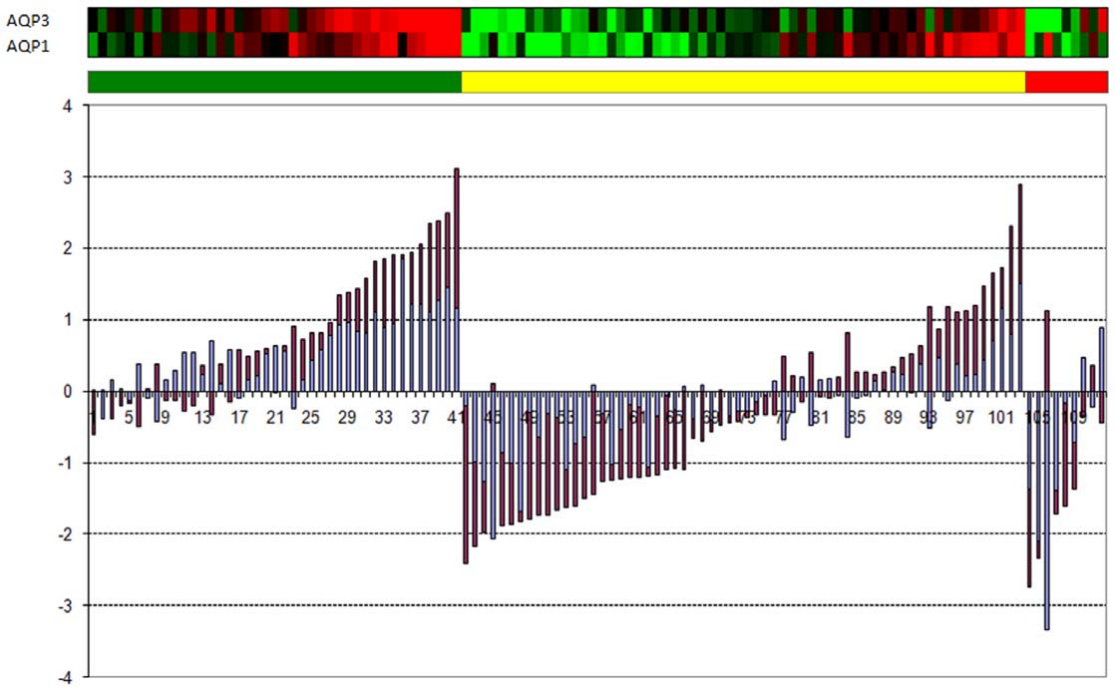
Heat map and stacked values showing the expression of the probe that correspond to AQP1 and AQP3 in Lapointe et al's prostate cancer dataset (Samples ordered by their total average value). Most of the control samples have a positive joint expression value (in green). A reduction is observed in primary prostate tumor samples (in yellow), with more than one half of the samples now having negative values. On the rightmost part of the figure, most of the lymph node metastasis samples (in red) have a strong negative total joint expression of these two biomarkers.

Does a similar pattern of aquaporin downregulation exist in prostate cancer? Wang et al. have looked at the expression and localization of AQP3 in human prostate using cell lines as well as patient samples. They have observed AQP3 mRNA “*in both normal and cancerous epithelia of human prostate tissues, but not in the mesenchyme. In the normal epithelia of the prostate, localization was limited to cell membranes, particularly the basolateral membranes. However, the expression of AQP3 protein in the cancer epithelia was not observed on the cell membranes.*” This finding seems to implicate the subcellular localization of AQP3 as a possible indicator of a transition to a more malignant phenotype. Lapointe's dataset allows us to see the downregulation of AQP3 and AQP1. A large subgroup of primary prostate tumors has reduced levels of AQP3 and AQP1 as most of the lymph node metastasis samples [Figure 18].

### Retrodictions, Postdictions, Predictions, Telomeres, non-coding RNAs and paraspeckles

One critique that we are aware we could receive is that the current manuscript presents a novel methodology and an underlying unifying theory based on *retrodictions* or *postdictions*. Indeed we have shown that the use of the *Normalized Shannon Entropy* and the *Information Theory* quantifiers (the *M-complexities* and the *Jensen-Shannon divergence*) allow to monitor cancer progression and to identify the best biomarkers that correlate with the transcriptomic changes. Our approach works in a *retrodiction* way in that it looks at data already obtained by other studies, but gives a unifying framework to track cancer progression. For instance, on True et al*'s* dataset, our unifying hallmark of cancer gives not only MAOA, which was already identified in the original publication, but also AMACR, CD40, CDK4, etc. are very important biomarkers for prostate cancer. Analogously, the identification of KLK3/PSA in Lapointe's dataset is another important retrodiction which shows the power of the method.

In some sense our approach also works in a *postdiction* way, as it helps to evaluate the speculation that cancer cells have “*an entropic devolution*”. Our results show that the variations of *Normalized Shannon Entropy* and *Jensen-Shannon divergences* indeed give measurable changes, and that these changes are related to important biomarkers in the two types of cancer studied in this work.

In addition, we remark that we are literally making *hundreds*, or even *thousands of predictions*. The results in the ‘Supplementary Material’ provide this information for the detailed scrutiny of our peers. We believe that other probes with gene expression patterns in high correlation with the probes discussed in this paper, and perhaps less studied by immunohistochemistry and other methods in the two cancer types studied here, are worth exploring as a group of biomarkers. These predictions can be tested with further studies on staging and patient stratification.

A very recent study by Ballal et al. have linked BRCA1 to telomere length and maintenance and its loss from the telomere in response to DNA damage [720] (see also [721]). We have previously mentioned that BRCA1 is a conspiquous biomarker arising from the analysis of True et al.'s dataset using our methods. We found this to correlate with a preivous study that showed that BRCA1 has a reduced expression in immortalized prostate epithelial cells before and after their conversion to tumorigenicity [493]. We also mentioned that the knockdown of BRCA1 leads to anaccumulation of multinucleated cells [492], preserving chromosomal stability [490]. Ballal et al. telomeric ChIP assays to detect BRCA1 at the telomere and reported time-dependent loss of BRCA1 from the telomere following DNA damage. Due to the role of telomeres in maintaining chromosomal stability [722] and the inverse correlation of telomere length and divergent karyotypes in prostate cancer cell lines [723], [724] (as well as the recognized role of telomere dysfunction in the induction of apoptosis or senescence in vivo [725], [726], [727], [728], [729], [730], increase of mutation rates [731], DNA fragmentation [732], and their relation with DNA damage signalling [733]), we checked for other probes of genes involved in telomeric function.

**Figure 19:**
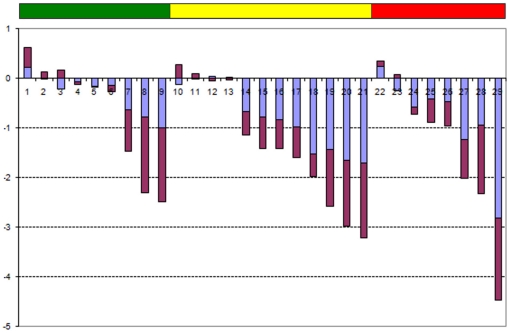
The stacked average gene expression of probes corresponding to BRCA1 and TERF2 (telomeric repeat binding factor 2) in True et al'*s* prostate cancer dataset. The first group of samples (1 to 9 in green) correspond to Gleason 3 pattern, indicating that most of the samples in this group have no significantly reduced expression of this pair of genes. The second group of columns (10 to 21 in yellow) correspond to Gleason 4 patterns and the last 8 columns (22 to 29 in red) correspond to Gleason 5 samples. A very recent study by Ballal et al. have linked BRCA1, to telomere length and maintenance and its loss from the telomere in response to DNA damage [720] (see also [721]). There is an increasing trend of dowregulation, so it would be interesting to evaluate if indeed this pair of proteins could be an early marker of dowregulation useful to evaluate samples with Gleason pattern 2, or if may constitute a biomarker useful to distinguish a prostate cancer subtype.

From those which we were able to identify in True et al's dataset, we have found a strong high correlation of the expression of BRCA1 with TERF2/TRF2 (telomeric repeat binding factor 2) [734] and a negative correlation with the expression pattern of TERF2IP (telomeric repeat binding factor 2, interacting protein) [Figure 19].

Finally, one particular type of probes has also caught our attention, and we would like to refer to them before concluding this section.

With the denomination of ‘non-coding RNA’ we identify those RNA molecules which are functional but that are not translated into proteins. Many microarray chips contain probes that are annotated as ‘non-protein coding’, indicating that there might be some valuable expression data that we can also mine for information. We note that our method, although employing transcriptomic data, does not limit its application to protein-coding information, and that the combined use of protein-coding and non-coding protein probe expression would allow a more comprehensive view of the transcriptional state of the cell.

**Figure 20:**
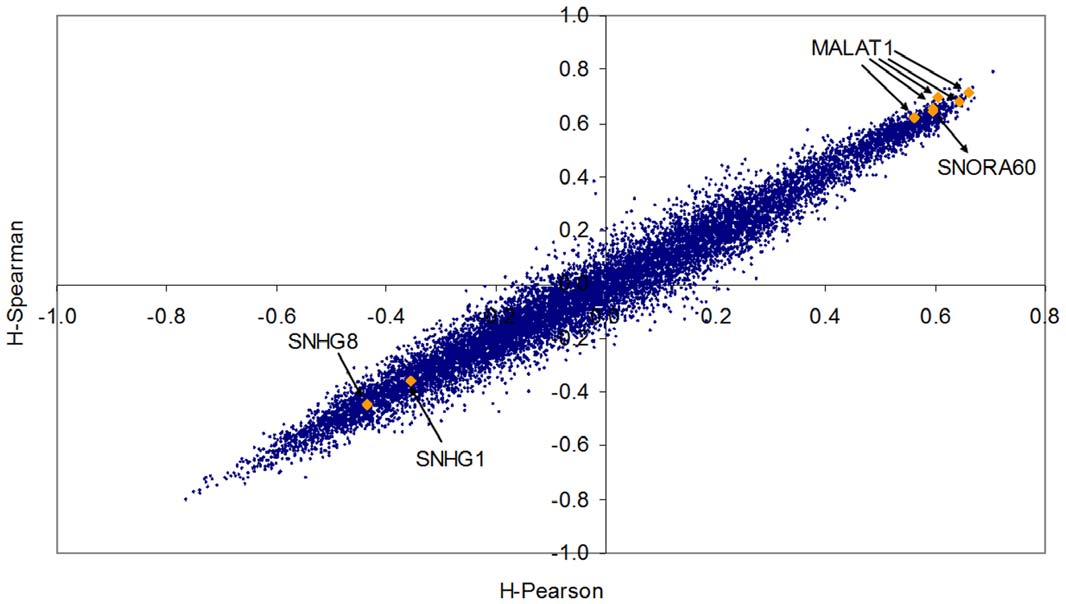
Non-coding RNAs and prostate cancer. We present again a scatter plot of Spearman versus Pearson correlation values of the probe expression of 13,188 probes in True et al*'*s prostate cancer dataset with the *Normalized Shannon Entropy* values of the samples. All blue dots correspond to one of the probes, but the only difference with Figure 16 is that we have now highlighted the position of s ome probes which have been annotated as corresponding to “non-coding RNAs”. In particular, we highlight those of MALAT1 (Metastasis associated lung adenocarcinoma transcript 1, (non-protein coding)), SNORA60 (small nucleolar RNA, H/ACA box 60); both increasingly downregulated, SNHG1 (small nucleolar RNA host gene 1 (non-protein coding)) and SNHG8 (small nucleolar RNA host gene 8 (non-protein coding)). The probes for MALAT1/MALAT-1 [742], [746], [747], [748], [749], [750], [751], [752], [753], [754], [755], [756], [757], [758] have a very conspiquous position, which we could judge *a priori* to be equivalent in relevance to those of the previously discussed roles of SFPQ, CD40, BRCA1, and TP53 (see Figure 16). MALAT1 has been recently pointed as a biomarker in primary human lobular breast cancer as a result of an analysis of over 132,000 Roche 454 high-confidence deep sequencing reads. Within the thousands of novel non-coding transcripts of the breast cancer transcriptome, Guffanti *al.*, identified more than three hundred reads corresponding to MALAT1 [749]. This non-coding RNA, first identified in 2003 in non-small cell lung cancer, was shown to be highly expressed (relative to GAPDH) in lung, pancreas and prostate, but not in other tissues including muscle, skin, stomach, bone marrow, saliva, thyroid and adrenal glands, uterus and fetal liver (see figure four of Ref. [758]). Our results indicate that the reduction of expression of some non-coding RNAs, in particular of MALAT-1, and SNORA60 with respect to their normal expression in prostate, as well as the upregulation of SNHG8 and SNHG1 should be monitored as useful biomarkers to track disease staging and progression to a more malignant phenotype. Interestingly enough, a study published in 2006 by Nadminty et al. has shown that KLK3/PSA modulates several genes, reporting a 16.5 fold downregulation of MALAT1 [810]. While these results have been obtained using the human osteosarcoma cell line SaOS-2, our results indicate that MALAT1 expression in the normal prostate and in cancer cells could also be considered as a relevant biomarkers to be tested in the future.

Among non-protein coding, microRNAs [735] are gaining acceptance as key players in several cancers [736], [737], [738] (including prostate cancer [739], [740]), but the so-called “long non-coding RNAs” [741] are also gaining a place in the scenario of cancer biomarkers (see [742], and [743], [744], [745]). We thus turned our attention to these probes that have been annotated as “non-protein coding” and we highlight some of them that have very high correlation values with the *Normalized Shannon Entropy* in True et al*'*s prostate cancer dataset. In particular, the probes for MALAT1/MALAT-1 [742], [746], [747], [748], [749], [750], [751], [752], [753], [754], [755], [756], [757], [758] have a very conspiquous position (See Figure 20). They located very closely to other protein coding biomarkers that have also lost expression and have been discussed in this work like SFPQ, CD40, BRCA1, and TP53 (see **Figure 16**). MALAT1 has been recently pointed as a biomarker in primary human lobular breast cancer as a result of an analysis of over 132,000 Roche 454 high-confidence deep sequencing reads [749]. An international team, searching on thousands of novel non-coding transcripts of the breast cancer transcriptome, has been able to identify more than three hundred reads corresponding to MALAT1 [749]. This is a non-coding RNA which was identified in 2003 in non-small cell lung cancer, was shown to be highly expressed (relative to GAPDH) in lung, pancreas and prostate, but not in other tissues including muscle, skin, stomach, bone marrow, saliva, thyroid and adrenal glands, uterus and fetal liver [758]. MALAT-1, also known as NEAT2, is considered to be “extraordinarily conseved for a noncoding RNA, more so than even XIST” [754]. Our results indicate that the reduction of expression of some non-coding RNAs, in particular of MALAT-1, and SNORA60 with respect to their normal expression in prostate, as well as the upregulation of SNHG8 and SNHG1 should be monitored as useful biomarkers to track disease progression.

We will now address another non-coding RNA called NEAT1 which, like NEAT2, is also conserved in the mammalian lingeage. Before we move onto NEAT1, we will first recall a previous result. We have noted before the conspiquous position of SFPQ/PSF (Polypyrimidine tract-binding protein-associated splicing factor) in Figure 16. The expression of a probe for SPQF has the highest correlation with the values of the *Normalized Shannon Entropy*. We highlighted before that SFPQ/PSF is a putative regulator of growth factor-stimulated gene expression [498]. The loss of SFPQ expression during the progression of prostate cancer may be an important key to understand this disease or one of its subtypes. We have also mentioned that the AR/PSF complex interacts with the PSA gene (perhaps the most well-established prostate cancer biomarker) and that SFPQ/PSF inhibits AR transcriptional activity [499]. Kuwahara et al. showed that SFPQ together with NONO (Non-POU-domain-containing, octamer binding protein) and PSPC1 (Paraspeckle protein 1 alpha isoform, formerly known as PSP1) are expressed in mouse Sertoli cells of the testis and form complexes that function as coregulators of androgen receptor-mediated transcription [500]. While new research results [759] link SFPQ and NONO/P54NRB with the RAD51 family of proteins (largely regarded as another key protector of chromosome integrity as being involved in homologous recombination DNA repair), it is perhaps SFPQ and NONO's co-localization in *paraspeckles* that make this group also remarkable [760].

Paraspeckles [760], [761], [762], [763], [764], [765], [766], [767], [768], [769], [770], [771], [772], [773], [774], [775], [776], [777] are a novel nuclear compartment, of approximately 0.2–1 µm in size, discovered in 2002, by Fox et al. in Dundee Scotland, following the identification of the protein PSPC1 (AF448795) in the nucleolar proteomics project at Lamond's lab which is described well by Fox et al. [777]. Three years later, Fox, Bond and Lamond showed that NONO and PSPC1 form a heterodimer that localizes to paraspeckles in an RNA-dependent manner [773]. Paraspeckles are dynamic structures, observed in numbers that vary between 10 and 20, that seem to control gene expression via retention of RNA in the nucleus [772]. A long noncoding RNA called NEAT1/MEN epsilon/beta [754], [760], [762], [764], [778], that colocalizes with paraspeckles, seems to be integral to their structure. Depletion of NEAT1 erradicates paraspeckles and a biochemical analysis by Clemson et al indicates that the NEAT1 binds with paraspeckle proteins SFPQ/PSF, P54NRB/NONO and PSPC1. NEAT1 is also known as TncRNA (trophoblast-derived noncoding RNA) [754], [779], [780], [781], [782], [783], [784], [785], [786] and probes for TncRNA exist on this dataset, We have observed in True et al.'s dataset that there exists a high correlation between the *Normalized Shannon Entropy* with the expression of SFPQ/PSF, P54NRB/NONO, and TncRNA. Overall, this implies that the disruption of the function of the paraspeckles is correlated with the increasing signs of deterioration of normal transcriptomic state of the cells. While a causal relationship still needs to be proved, we admire the mathematical elegance of the *Normalized Shannon Entropy* of the samples, a global measure of the average expected surprisal of the transcriptome, which in turn has lead us to consider the dysfunction of the smallest nuclear body as a putative biomarker of disease progression. The role of SFPQ/PSF in the control of tumorigenesis is under investigation [787] and the information coming from these studies would need to be integrated with their role, together with P54NRB/NONO and TncRNA, in paraspeckles if we want to achieve a better understanding of these mechanisms.

### Conclusions

In this contribution we have shown that for the melanoma and prostate cancer datasets studied, the quantitative changes of Information Theory measures, *Normalized Shannon Entropy*, *Jensen–Shannon divergence* and the novel *Statistical Complexity* quantifiers defined here are in high correlation with gene expression changes of well-established biomarkers associated to cancer progression. In addition, variations of the basic technique (i.e. a modified form of statistical complexity) which allows us to better understand the phenotypic changes observed in these samples which are associated with the progression and the transitions of the gene expression profiles. For instance, in a properly defined *Statistical Complexity* vs. *Entropy* plane, on a melanoma dataset first studied in Ref. [110], samples appear in well differentiated “clusters”. These clusters correlate well with the phonotypic characteristics of *normal skin*, *nevi*, *primary* and *metastatic melanoma*. In this “Complexity vs. Entropy” plane, primary melanomas samples appear “bridging” benign nevi and metastatic melanoma samples. Our results may also suggest that the evolution of metastatic melanoma leads to at least two different subtypes.

The *Normalized Shannon Entropy* of a transcriptional sample profile is calculated associating the measured expression values of a gene with the relatively probability of being expressed. We have observed that, in general, the transcriptomes of tumour progressing cells tend to have lower values of *Normalized Shannon Entropy* than normal ones. Given a population of normal cells of a given tissue type it is then possible to compute useful measure of divergence of cancer cell profiles from the normal expression average profile, in terms of Information Theory quantifiers, the Shannon Eveness normalized entropy and generalized statistical complexity [788], [789], [790].

In addition, our observation of the correlation of the statistical complexity of tumours with its natural progression allows an unprecedented way of finding biomarkers that links with the gradual deterioration of the genome integrity. The proposed methodology uncovered, for the first time, evidence of the putative role of impared centrosome cohesion in melanoma progression.

Statistical complexity has then been able to pinpoint otherwise unrecognized biomarkers in concert with existing ones, reinforcing the view that “*chromosomal chaos*” and “*cancer as a chromosomal disease*” can be a useful guiding principle to understand the molecular biology of cancer and uncover the timeline of its progression. This is a powerful method to uncover “oncosystems” instead of “oncogenes”. “Oncosystems” are a highly differentially disregulated set of genes that, if linked with the molecular “hallmarks of cancer” described in the introduction, and existing databases with putative common functional genomic annotations, can help to understand the biological progression pathways that drive the disease.

On one of the prostate cancer dataset studied (obtained from a previous published study, [44]), we observe a gradual pattern of reduction of *Normalized Shannon Entropy* from three well characterized tissue types: *normal prostate, primary prostate tumours* and *lymph node metastases*. On a different dataset on prostate cancer (from Ref [332]), we observe that a group of samples having Gleason patterns 4 and 5 (two patterns which are typically associated to an aggressive phenotype) have lower *Normalized Shannon Entropy* values than a subset of Gleason pattern 3 (a pattern which is normally associated to a less aggressive phenotype but which nevertheless is still of clinical concern). However, a group of samples having Gleason patterns 3, 4, and 5 is revealed; this mixed cluster has a mid-range entropy. This is an interesting fact which correlates with the limitations observed in Ref. [332]. We note the authors' comment: “*We were unable to identify a cohort of genes that could distinguish between pattern 4 and 5 cancers with sufficiently high accuracy to be useful, suggesting a high degree of similarity between these cancer histologies or substantial molecular heterogeneity in one or both of these groups.*” Our results provide a conciliatory middle ground that explains the perceived clinical usefulness of Gleason pattern classification, widely used around the world, while at the same time reveals the reason for the difficulties of obtaining a good transcriptional signature for the other two patterns [791].

We have seen, through a detailed discussion of several biomarkers in three different datasets, that the variation of the gene expression distributional profile can be characterized via Information Theory quantifiers. Our study also showed that current established biomarkers of the two diseases studied seem to correlate with those that best co-variate with these quantifiers. For instance, AMACR, in our second prostate cancer dataset studied, naturally appears as one of the most correlated genes (in both the Pearson and the Spearman sense) with the pattern of variation of Entropy of the samples. Together with MAOA, which is the highlighted gene in True et al.'s [332] original publication, AMACR is now being recognized as one of the best biomarkers in primary prostate cancer with approximately 180 publications dedicated to it in the past five years. We have also shown that many gene probes that best correlate with the divergence of the normal tissue profile have been identified as useful biomarkers (via other accepted validation methods). This said, the use of other sources of information, like pathway or gene ontology databases has lead as to the identification of other cell processes that may be altered.

We have presented a unifying hallmark of cancer, the cancer cell's transcriptome changes its *Normalized Shannon Entropy* (as measured by high-througput technologies), while it increments its physical Entropy (via creation of states we might not measure with our devices). This hallmark allows, via the use of the *Jensen-Shannon divergence*, to identify the arrow of time of the process, and helps to map the phenotypical and molecular hallmarks of cancer as major converging trends of the transcriptome. The methodology has produced remarkable postdictions and retrodictions that show that it can predictively guide biomarker discovery.

## Materials and Methods

We refer the reader to the original publications for details of methods for data collection, but we highlight here some aspects that are important to understand the data generation process for the purpose of our analysis.

### Lapointe et al.'s dataset (File S1)

Samples were obtrained from radical prostatectomy surgical procedures. Samples are labelled as “tumors” if they contain at least 90% of cancerous epithelial cells, and they were considered as “non-tumor” if they contain no tumor epithelium and are from the noncancerous region of the prostate. The later samples were labelled “normals” although the authors alert that some may contain dysplasia. In this dataset, Lapointe et al. have performed a gene expression profiling by using cDNA microarrays containing 26,260 different human genes (UniGene clusters). Using 50 µg of total RNA from prostate samples Cy5-labeled cDNA was prepared and Cy3-labeled cDNA used 1.5 µg of mRNA common reference, pooled from 11 human cell lines (see Ref. [792]). The fluorescence ratios were subsequently normalized by mean centering genes for each array, a relatively standard procedure. In addition, to minimize potential print run specific bias, Lapointe et al. report that ratios were then mean centered for each gene across all arrays according to Ref. [793]. We have only used the genes that the authors report in their first figure, 5,153 genes that have been well measured and have significan variation in some of the samples. For the other details of their matrials and methods we refer the readers to the Supporting Notes and the Materials and Methods section of their original publication [44].

### Haqq et al.'s dataset (File S2)

Samples were obtained from nevus volunteers and melanoma patients and only those samples that have more than 90% of tumor cells were profiled. The 20,862 cDNAs used (Research Genetics, Huntsville, AL) represent 19,740 independent loci. (Unigene build 166).median of ratio values from the experiment were subjected to linear normalization in nomad (which can be accessed at http://derisilab.ucsf.edu), log-transformed (base 2), and filtered for genes where data were present in 80% of experiments, and where the absolute value of at least one measurement was >1.

### True et al's dataset (File S3)

In this dataset, samples have information of 15,488 spots per array, with a total of 7,700 unique cDNAs represented. The samples were obtained from frozen tissue blocks from 29 radical prostatectomies accessioned and selected to represent Gleason grades 3, 4, and 5. The samples are “treatment naïve”, meaning that they were also selected such that their gene expression profile is also and the absence of any bias that the treatment before prostatectomy. The frozen sections (8 µm) were cut from optimal cutting temperature medium blocks and immediately fixed in cold 95% ethanol. Around 5,000 epithelial cells from both histologically benign glands and cancer glands were separately laser-capture microdissected (LCM). The authors of the study have also been very careful to include *only one* Gleason pattern in each laser-captured cancer sample, following a process in which the patterns were assessed independently by two investigators.The matched benign epithelium was captured for each cancer sample for a total of 121 samples.

An important characteristic of this dataset is the normalization procedure. For each spot and in each channel (Cy3 and Cy5), True et al. substracted the median background intensity from the median foreground intensity, and subsequently the log ratios of cancer expression to benign expression were computed. These ratios were obtained by first dividing the background-subtracted intensities (Prostate Cancer/Benign) and then taking the logarithm base 2. In the case that the median background intensity was greater than the median foreground intensity, the spot was considered missing. We refer to the original publication for the other aspects of imputation, spot quality and filtering, but, like in Lapointe et al*'s* study, they also filter to keep informative (expression ratios of benign versus cancer should at least be 1.5-fold or greater in at least half of one of the Gleason groups as one of the selection criteria).

### Normalized Shannon Entropy, Jensen-Shannon Divergence and Statistical Complexity

#### *Shannon Entropy*

In many circumstances, experimental measurements are associated with the accumulation of individual results which, ultimately, qualitatively and quantitatively characterized our experimental observations. The presence (or absence) of a particular result of an individual experimental measure is called an *event*. An event which can take one of several possible values is called a *random variable*. Analogously, a *random event* is an event that can either fail to happen, or happens, as a result of an experiment. An event is *certain* if it can not fail to happen and it is said to be *impossible* if it can never happen.

Following Andreyev [794], we will define the *probability p(x)* of an event *x*, as the *theoretical frequency* of the event *x* about which the *actual frequency occurrence of the event shows a tendency to fluctuate* as the experiment is repeated many times. The *Shannon information content of an event x* (or the *surprisal of an event x*, [795]), is defined as
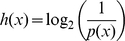
Following McKay [796], an *ensamble X* is a triple 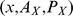, where *x* is the value of a random variable, which takes on one of a set of possible values, 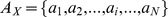, having probabilities 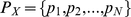, with 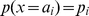, 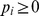 and 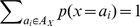.

The *Shannon Entropy of an ensemble X* (also known as the *uncertainty of X*), denoted as *H[X]*, is defined to be the *average Shannon information content*. It is the *average expected surprisal* for an infinitely long series of experiments. We use the theoretical frequencies to compute this average, and then we have
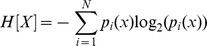
Suppose that we have a fair dice, the theoretical frequency of an event ‘*the dice shows a three*’ is 1/6, (if the dice is assumed fair, the theoretical frequency is the same for any number from 1 to 6). In that case a hypothetical experimentalist guessing will have an average expected surprise of *H[X]* = 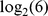. We note the two natural bounds that the entropy can have. The Shannon Entropy of an ensemble *X* is always greater or equal to zero. It can only be zero if 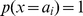 for only one of the *N* elements of 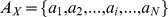. On the other hand, the *Shannon Entropy* is maximized in the case that 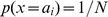. This is the so-called “*equiprobable distribution*”, a uniform probability distribution over the finite set.

#### *Transcriptional Shannon Entropy*

Let 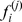 the expression value of probe *i* (*i* = 1,…, *N*) on sample *j* (*j* = 1, …, *M*). For each sample *j* we first normalize the expression values. We interpret them as the *theoretical frequency* of a single hybridization event. We then define a probability distribution function (PDF) over a finite set as:
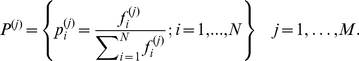
The *uniform (equiprobably) distribution* is defined as
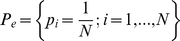
and the *average probability distribution* over all *M* samples as
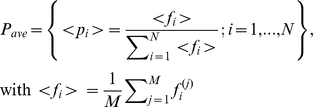
Let 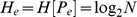, then in this paper we always use the *Normalized Shannon Entropy*, defined as:
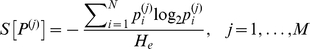



### The Jensen-Shannon divergence and the Statistical complexity measures

Given a probability distribution function over a discrete finite set, is then straightforward to calculate its *Normalized Shannon Entropy* if we have the theoretical frequencies. Several measures of “complexity” of a probability distribution function have been proposed. In this work we have used *Statistical Complexity* measures.

All the complexity measures used in this work are the product of a *Normalized Shannon Entropy* of the probability distribution function, and a divergence measure to a reference probability distribution function. We follow earlier proposals by López-Ruiz, Mancini and Calbet who first introduced a statistical complexity measure based on such a product in [797]. The *LMC-Statistical Complexity* is the product of the *Normalized Shannon Entropy*, *H[P]*, times the *disequilibrium*, *Q[P]*; the latter given by the Euclidean distance from *P* to *P*_e_, the uniform probability distribution over the ensemble. In this paper we used a later modification which we refer as the *MPR-Statistical Complexity* [43] which replaces the Euclidean distance between *P* to *P*_e_ by the *Jensen-Shannon divergence* [788], [798]. The *Jensen-Shannon divergence* is linked in physics to the thermodynamic length [799], [800], [801], [802].

We define the *MPR-Statistical complexity* [790] as:
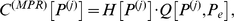
where 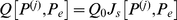, *Q_0_* is a normalization factor, and 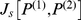 is the *Jensen-Shannon's divergence* between two probability density functions *P^(1)^* and *P^(2)^*, which in turn is defined as
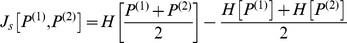
In this work, in many cases we compute the *Jensen-Shannon divergences* of a probability with a probability of reference which is not the uniform probability distribution over the ensemble. In general, it is the average over a subset of probability distribution functions which are consider to be either the “initial” of “final” states of interest. Let 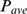 be such an average, then the *M-Statistical Complexity of a probability distribution function* 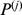, given a 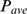 of reference, is given by
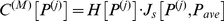



#### *An illutrative example*

In order to discuss a relatively simple example that can intuitively provide a grasp of the basic mathematical principles of *Information Theory* we present a hypothetical “gene expression” dataset involving four samples each with the expression of five unique probes corresponding to five genes (not necessarily different) as follows in Table 3.

**Table 3:** An example dataset to illustrate the principles of *Shannon Entropy* and the *Information Theory* quantifiers used in this work.

	Gene 1	Gene 2	Gene 3	Gene 4	Gene 5
Sample 1	4	3	2	1	0.1
Sample 2	0.1	1	2	3	4
Sample 3	5	2	5	1	3
Sample 4	2	2	2	2	2

The matrix is a hypothetical gene expression dataset containing four samples each consisting of probes for five genes.

**Figure 21:**
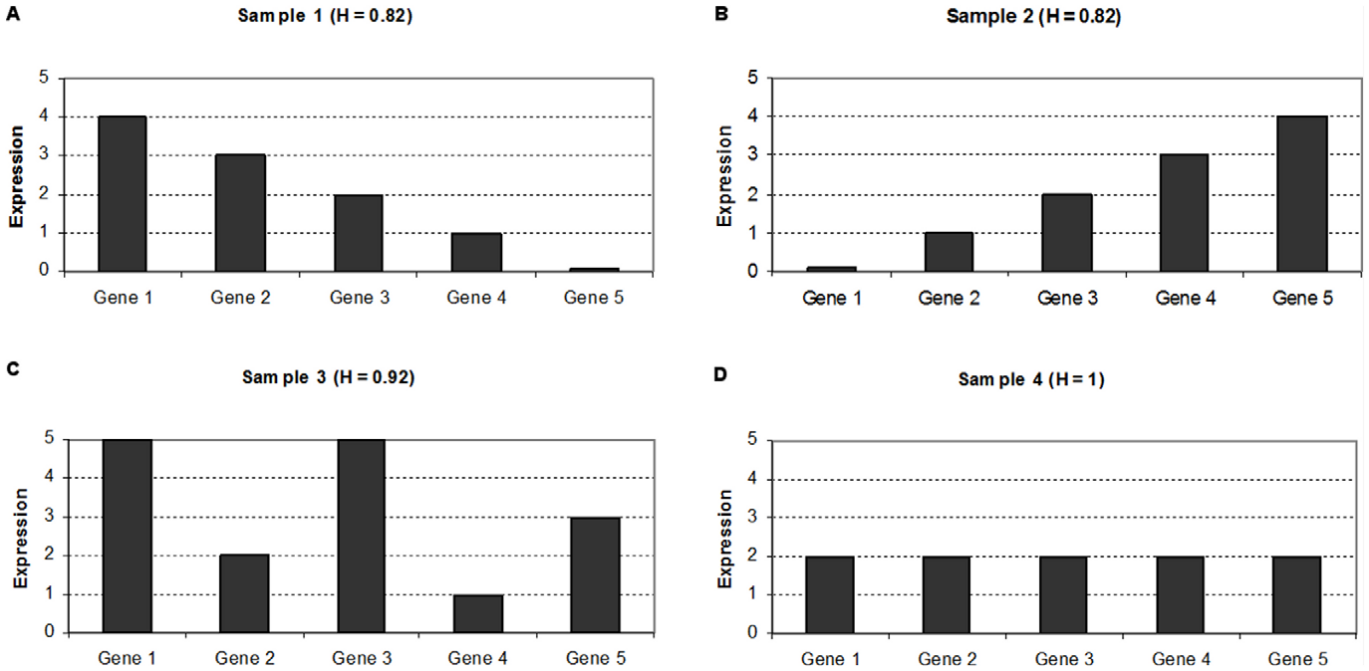
*Normalized Shannon Entropy* values *(H)* of the samples from **Table 3**. Sample 4 has the largest attainable value since the expression of all probes is the same. Samples 1 and 2, which have the same set of expression values, although in different probes, have the same value of *Normalized Shannon Entropy*. As a consequence, there is a need for another quantifier of gene expression to address the permutational indistinguishability of these two expression profiles. The Jensen-Shannon divergence provides a natural alternative (see Table 4).

One of the quantifiers that we use in this contribution describes a measure of order for a sample: the *Normalized Shannon Entropy* also known as *Shannon Evenness Index* [803]. This section focuses on this quantifiers use and importance (refer to the ‘Materials and Methods’ section to see how this measure is calculated). In Sample 4 all probes have the same expression therefore it has the highest achievable value of *Normalized Shannon Entropy (H = 1)*. The *Normalized Shannon Entropy* values for samples 1 and 2 are the same *(H = 0.82)*. Sample 3, which tends to be less peaked and has the two most significantly expressed genes with the same value, has a higher value of *Normalized Shannon Entropy (H = 0.92)* (see Figure 21).

This simple example shows that the *Normalized Shannon Entropy* variations of the gene expression profile convey information about global transcriptomic changes; however, this measure *alone* is not enough to characterize the deviations from normal tissue profiles. For example, assume that Sample 1 is the normal profile of a particular tissue type. Assume that Sample 3 is the profile of a cancer cell that originated from that tissue type, the variation of *Normalized Shannon Entropy* can be related to this malignant change. However, as Sample 2 illustrates, *Normalized Shannon Entropy* is not enough to let us to measure the variation from a profile and at least another *Information Theory* quantifier is needed. We resort to *Statistical Complexity quantifiers*, which in turn use the *Jensen-Shannon divergence* [798] to provide this complementary dimension [800] (refer to the ‘Materials and Methods’ section for a mathematical definition of the *Jensen-Shannon divergence*).

Figure 21 shows how the *Jensen-Shannon divergence* helps us to evaluate the variation between profiles. Samples 1 and 2, as perhaps intuitively expected, have the largest divergence between them, their *Jensen Shannon divergence* is *0.286636 (JS(1,2) = JS(2,1) = 0.286636)*. The two “closest” pair of profiles correspond to Samples 3, and 4, *(JS(3,4) = JS(4,3) = 0.035851)*. See Table 4.

**Table 4:** *Jensen-Shannon divergence* values using the example introduced in Table 3.

Samples	1	2	3	4
1	0	0.286636	0.077849	0.82685
2	0.286636	0	0.157463	0.082685
3	0.077849	0.157463	0	0.035851
4	0.82685	0.082685	0.035851	0

While samples 1 and 2 have the same *Normalized Shannon Entropy*, they have very different gene expression profiles and this is reflected in their mutual *Jensen-Shannon divergence* which is 0.286636. The sample with the smallest divergence to the equiprobability distribution sample 4 is sample 3.

**Figure 22:**
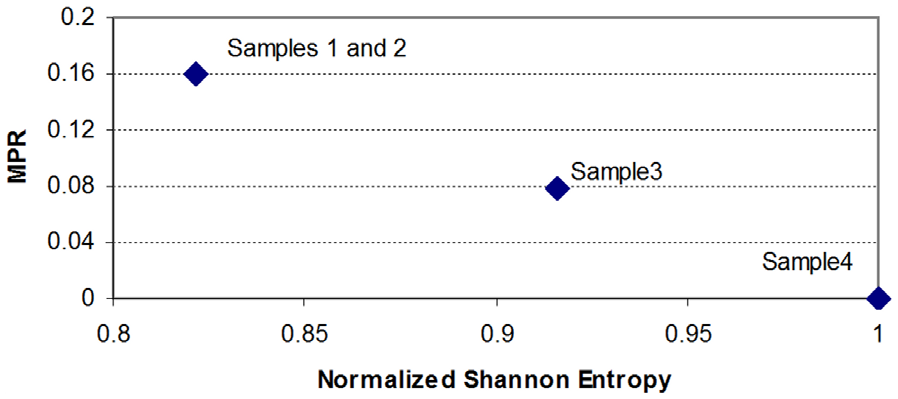
*MPR-Statistical Complexity* as a function of the *Normalized Shannon Entropy* for the example dataset from **Table 3**. The *MPR-Statistical complexity* is proportional to the *Normalized Shannon Entropy* (labelled ‘MPR’, *y*-axis) of a sample and the *Jensen-Shannon divergence* of the sample and a hypothetical sample with an equiprobability distribution of gene expression.

Let 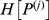 be the *Normalized Shannon Entropy* of a transcriptional sample profile, then the *MPR-Statistical Complexity* 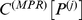 is defined as being proportional to the product of the *Normalized Shannon Entropy* times the *Jensen-Shannon divergence* of the profile with the equiprobable distribution (in the example above the equiprobable distribution is that of Sample 4). Then we have
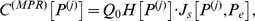
Where 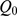 is a normalization factor. Once again, we refer to the ‘Materials and Methods’ sections for the accompanying formal mathematical presentation. As a consequence, we can plot the *MPR-Statistical Complexity* of the samples of our example as a function of the *Normalized Shannon Entropy* as can be seen in Figure 22.

#### *Annotated genes*

A full list of gene references in this paper along with their descriptions from iHOP (http://www.ihop-net.org/UniPub/iHOP/) can be found in supplementary material reference File S5.

## Supporting Information

File S1 Haqq Data Set Supporting File
(3.92 MB XLS)

File S2 Lapointe Data Supporting file
(1.51 MB XLS)

File S3 True Data Supporting File
(7.23 MB XLS)

File S4 List of references for research into NFKappa-B as a target for the intervention in prostate cancer
(0.10 MB DOC)

File S5 A full list of gene references in this paper along with their descriptions from iHOP (http://www.ihop-net.org/UniPub/iHOP/).
(0.23 MB DOC)
