# Supplementary material for: Cancer Biomarker Discovery: The Entropic Hallmark
Source: PLoS One. 2010 Aug 18;5(8):e12262. doi: 10.1371/journal.pone.0012262 (PMC2923618; doi:10.1371/journal.pone.0012262)
Supplement: File S4 — List of references for research into NFKappa-B as a target for the intervention in prostate cancer (0.10 MB DOC) [file pone.0012262.s004.doc]

**List of references for research into NFKappa-B as a target for the intervention in prostate cancer**

Uzzo RG, Crispen PL, Golovine K, Makhov P, Horwitz EM, et al. (2006) Diverse effects of zinc on NF-kappaB and AP-1 transcription factors: implications for prostate cancer progression. Carcinogenesis 27: 1980-1990.

Singh RP, Agarwal R (2006) Prostate cancer chemoprevention by silibinin: bench to bedside. Mol Carcinog 45: 436-442.

Raffoul JJ, Wang Y, Kucuk O, Forman JD, Sarkar FH, et al. (2006) Genistein inhibits radiation-induced activation of NF-kappaB in prostate cancer cells promoting apoptosis and G2/M cell cycle arrest. BMC Cancer 6: 107.

Kwon O, Kim KA, Kim SO, Ha R, Oh WK, et al. (2006) NF-kappaB inhibition increases chemosensitivity to trichostatin A-induced cell death of Ki-Ras-transformed human prostate epithelial cells. Carcinogenesis 27: 2258-2268.

Huerta-Yepez S, Vega M, Garban H, Bonavida B (2006) Involvement of the TNF-alpha autocrine-paracrine loop, via NF-kappaB and YY1, in the regulation of tumor cell resistance to Fas-induced apoptosis. Clin Immunol 120: 297-309.

Ho SM, Leung YK, Chung I (2006) Estrogens and antiestrogens as etiological factors and therapeutics for prostate cancer. Ann N Y Acad Sci 1089: 177-193.

Gazak R, Walterova D, Kren V (2007) Silybin and silymarin--new and emerging applications in medicine. Curr Med Chem 14: 315-338.

Deeb D, Jiang H, Gao X, Al-Holou S, Danyluk AL, et al. (2007) Curcumin [1,7-bis(4-hydroxy-3-methoxyphenyl)-1-6-heptadine-3,5-dione; C21H20O6] sensitizes human prostate cancer cells to tumor necrosis factor-related apoptosis-inducing ligand/Apo2L-induced apoptosis by suppressing nuclear factor-kappaB via inhibition of the prosurvival Akt signaling pathway. J Pharmacol Exp Ther 321: 616-625.

Deeb D, Gao X, Dulchavsky SA, Gautam SC (2007) CDDO-me induces apoptosis and inhibits Akt, mTOR and NF-kappaB signaling proteins in prostate cancer cells. Anticancer Res 27: 3035-3044.

Crispen PL, Uzzo RG, Golovine K, Makhov P, Pollack A, et al. (2007) Vitamin E succinate inhibits NF-kappaB and prevents the development of a metastatic phenotype in prostate cancer cells: implications for chemoprevention. Prostate 67: 582-590.

Colgate EC, Miranda CL, Stevens JF, Bray TM, Ho E (2007) Xanthohumol, a prenylflavonoid derived from hops induces apoptosis and inhibits NF-kappaB activation in prostate epithelial cells. Cancer Lett 246: 201-209.

Christensen MJ, Nartey ET, Hada AL, Legg RL, Barzee BR (2007) High selenium reduces NF-kappaB-regulated gene expression in uninduced human prostate cancer cells. Nutr Cancer 58: 197-204.

Chen W, Wu W, Zhao J, Yu C, Liu W, et al. (2007) Molecular cloning and preliminary analysis of the human alpha-methylacyl-CoA racemase promoter. Mol Biol Rep.

Baritaki S, Katsman A, Chatterjee D, Yeung KC, Spandidos DA, et al. (2007) Regulation of tumor cell sensitivity to TRAIL-induced apoptosis by the metastatic suppressor Raf kinase inhibitor protein via Yin Yang 1 inhibition and death receptor 5 up-regulation. J Immunol 179: 5441-5453.

Araki S, Omori Y, Lyn D, Singh RK, Meinbach DM, et al. (2007) Interleukin-8 is a molecular determinant of androgen independence and progression in prostate cancer. Cancer Res 67: 6854-6862.

Adachi M, Sakamoto H, Kawamura R, Wang W, Imai K, et al. (2007) Nonsteroidal anti-inflammatory drugs and oxidative stress in cancer cells. Histol Histopathol 22: 437-442.

Yemelyanov A, Gasparian A, Lindholm P, Dang L, Pierce JW, et al. (2006) Effects of IKK inhibitor PS1145 on NF-kappaB function, proliferation, apoptosis and invasion activity in prostate carcinoma cells. Oncogene 25: 387-398.

Yatkin E, Bernoulli J, Santti R (2006) Activation of NF-kappaB in association with prostate carcinogenesis in noble rats. Ann N Y Acad Sci 1089: 282-285.

Winnicka K, Bielawski K, Bielawska A (2006) Cardiac glycosides in cancer research and cancer therapy. Acta Pol Pharm 63: 109-115.

Warren G, Grimes K, Xu Y, Kudrimoti M, St Clair W (2006) Selectively enhanced radiation sensitivity in prostate cancer cells associated with proteasome inhibition. Oncol Rep 15: 1287-1291.

Takeyama K, Mitsuzawa H, Shimizu T, Konishi M, Nishitani C, et al. (2006) Prostate cell lines secrete IL-8 in response to Mycoplasma hominis through Toll-like receptor 2-mediated mechanism. Prostate 66: 386-391.

Syeda F, Grosjean J, Houliston RA, Keogh RJ, Carter TD, et al. (2006) Cyclooxygenase-2 induction and prostacyclin release by protease-activated receptors in endothelial cells require cooperation between mitogen-activated protein kinase and NF-kappaB pathways. J Biol Chem 281: 11792-11804.

Song HJ, Sneddon AA, Heys SD, Wahle KW (2006) Induction of apoptosis and inhibition of NF-kappaB activation in human prostate cancer cells by the cis-9, trans-11 but not the trans-10, cis-12 isomer of conjugated linoleic acid. Prostate 66: 839-846.

Sarkar FH, Adsule S, Padhye S, Kulkarni S, Li Y (2006) The role of genistein and synthetic derivatives of isoflavone in cancer prevention and therapy. Mini Rev Med Chem 6: 401-407.

Sanlioglu AD, Koksal IT, Karacay B, Baykara M, Luleci G, et al. (2006) Adenovirus-mediated IKKbetaKA expression sensitizes prostate carcinoma cells to TRAIL-induced apoptosis. Cancer Gene Ther 13: 21-31.

Reddy GP, Barrack ER, Dou QP, Menon M, Pelley R, et al. (2006) Regulatory processes affecting androgen receptor expression, stability, and function: potential targets to treat hormone-refractory prostate cancer. J Cell Biochem 98: 1408-1423.

Nishimura K, Takayama H, Nakayama M, Nonomura N, Okuyama A (2006) [Molecular-targeted therapy for hormone-refractory prostate cancer]. Hinyokika Kiyo 52: 487-490.

Nadiminty N, Lou W, Lee SO, Mehraein-Ghomi F, Kirk JS, et al. (2006) Prostate-specific antigen modulates genes involved in bone remodeling and induces osteoblast differentiation of human osteosarcoma cell line SaOS-2. Clin Cancer Res 12: 1420-1430.

Mori A, Lehmann S, O'Kelly J, Kumagai T, Desmond JC, et al. (2006) Capsaicin, a component of red peppers, inhibits the growth of androgen-independent, p53 mutant prostate cancer cells. Cancer Res 66: 3222-3229.

Misra UK, Deedwania R, Pizzo SV (2006) Activation and cross-talk between Akt, NF-kappaB, and unfolded protein response signaling in 1-LN prostate cancer cells consequent to ligation of cell surface-associated GRP78. J Biol Chem 281: 13694-13707.

Ma Y, Lakshmikanthan V, Lewis RW, Kumar MV (2006) Sensitization of TRAIL-resistant cells by inhibition of heat shock protein 90 with low-dose geldanamycin. Mol Cancer Ther 5: 170-178.

Li Z, Chen Y, Cao D, Wang Y, Chen G, et al. (2006) Glucocorticoid up-regulates transforming growth factor-beta (TGF-beta) type II receptor and enhances TGF-beta signaling in human prostate cancer PC-3 cells. Endocrinology 147: 5259-5267.

Lessard L, Karakiewicz PI, Bellon-Gagnon P, Alam-Fahmy M, Ismail HA, et al. (2006) Nuclear localization of nuclear factor-kappaB p65 in primary prostate tumors is highly predictive of pelvic lymph node metastases. Clin Cancer Res 12: 5741-5745.

Labbozzetta M, Notarbartolo M, Poma P, Giannitrapani L, Cervello M, et al. (2006) Significance of autologous interleukin-6 production in the HA22T/VGH cell model of hepatocellular carcinoma. Ann N Y Acad Sci 1089: 268-275.

Kim JH, Xu C, Keum YS, Reddy B, Conney A, et al. (2006) Inhibition of EGFR signaling in human prostate cancer PC-3 cells by combination treatment with beta-phenylethyl isothiocyanate and curcumin. Carcinogenesis 27: 475-482.

Kao JJ (2006) The NF-kappaB inhibitor pyrrolidine dithiocarbamate blocks IL-1beta induced hyaluronan synthase 1 (HAS1) mRNA transcription, pointing at NF-kappaB dependence of the gene HAS1. Exp Gerontol 41: 641-647.

Josson S, Xu Y, Fang F, Dhar SK, St Clair DK, et al. (2006) RelB regulates manganese superoxide dismutase gene and resistance to ionizing radiation of prostate cancer cells. Oncogene 25: 1554-1559.

Jiang Z, Clemens PR (2006) Cellular caspase-8-like inhibitory protein (cFLIP) prevents inhibition of muscle cell differentiation induced by cancer cells. FASEB J 20: 2570-2572.

Jiang BH, Liu LZ, Schafer R, Flynn DC, Barnett JB (2006) A novel role for 3, 4-dichloropropionanilide (DCPA) in the inhibition of prostate cancer cell migration, proliferation, and hypoxia-inducible factor 1alpha expression. BMC Cancer 6: 204.

Hwang YS, Hodge JC, Sivapurapu N, Lindholm PF (2006) Lysophosphatidic acid stimulates PC-3 prostate cancer cell Matrigel invasion through activation of RhoA and NF-kappaB activity. Mol Carcinog 45: 518-529.

Hughes-Fulford M, Li CF, Boonyaratanakornkit J, Sayyah S (2006) Arachidonic acid activates phosphatidylinositol 3-kinase signaling and induces gene expression in prostate cancer. Cancer Res 66: 1427-1433.

Huangfu WC, Omori E, Akira S, Matsumoto K, Ninomiya-Tsuji J (2006) Osmotic stress activates the TAK1-JNK pathway while blocking TAK1-mediated NF-kappaB activation: TAO2 regulates TAK1 pathways. J Biol Chem 281: 28802-28810.

Hermani A, De Servi B, Medunjanin S, Tessier PA, Mayer D (2006) S100A8 and S100A9 activate MAP kinase and NF-kappaB signaling pathways and trigger translocation of RAGE in human prostate cancer cells. Exp Cell Res 312: 184-197.

Hassan F, Islam S, Tumurkhuu G, Naiki Y, Koide N, et al. (2006) Intracellular expression of toll-like receptor 4 in neuroblastoma cells and their unresponsiveness to lipopolysaccharide. BMC Cancer 6: 281.

Harkonen PL, Vaananen HK (2006) Monocyte-macrophage system as a target for estrogen and selective estrogen receptor modulators. Ann N Y Acad Sci 1089: 218-227.

Guo H, Yu JH, Chen K, Ye ZQ, Liu GC (2006) [Curcumin-induced apoptosis in androgen-dependent prostate cancer cell line LNCaP in vitro]. Zhonghua Nan Ke Xue 12: 141-144.

Guo H, Yu JH, Chen K, Ye ZQ (2006) [Curcumin-induced the expression of inhibitor kappaBalpha protein in human prostate cancer cells]. Zhonghua Wai Ke Za Zhi 44: 1256-1259.

Girvan AC, Teng Y, Casson LK, Thomas SD, Juliger S, et al. (2006) AGRO100 inhibits activation of nuclear factor-kappaB (NF-kappaB) by forming a complex with NF-kappaB essential modulator (NEMO) and nucleolin. Mol Cancer Ther 5: 1790-1799.

Dougall WC, Chaisson M (2006) The RANK/RANKL/OPG triad in cancer-induced bone diseases. Cancer Metastasis Rev 25: 541-549.

Lu Y, Xiao G, Galson DL, Nishio Y, Mizokami A, et al. (2007) PTHrP-induced MCP-1 production by human bone marrow endothelial cells and osteoblasts promotes osteoclast differentiation and prostate cancer cell proliferation and invasion in vitro. Int J Cancer 121: 724-733.

Yemelyanov A, Czwornog J, Chebotaev D, Karseladze A, Kulevitch E, et al. (2007) Tumor suppressor activity of glucocorticoid receptor in the prostate. Oncogene 26: 1885-1896.

Lessard L, Saad F, Le Page C, Diallo JS, Peant B, et al. (2007) NF-kappaB2 processing and p52 nuclear accumulation after androgenic stimulation of LNCaP prostate cancer cells. Cell Signal 19: 1093-1100

Son DJ, Park MH, Chae SJ, Moon SO, Lee JW, et al. (2007) Inhibitory effect of snake venom toxin from Vipera lebetina turanica on hormone-refractory human prostate cancer cell growth: induction of apoptosis through inactivation of nuclear factor kappaB. Mol Cancer Ther 6: 675-683.

Zhi H, Yang XJ, Kuhnmuench J, Berg T, Thill R, et al. (2009) SmgGDS is up-regulated in prostate carcinoma and promotes tumour phenotypes in prostate cancer cells. J Pathol 217: 389-397.

Vykhovanets EV, Shukla S, Maclennan GT, Vykhovanets OV, Bodner DR, et al. (2009) Il-1beta-induced post-transition effect of NF-kappaB provides time-dependent wave of signals for initial phase of intrapostatic inflammation. Prostate.

Singh-Gupta V, Zhang H, Banerjee S, Kong D, Raffoul JJ, et al. (2009) Radiation-induced HIF-1alpha cell survival pathway is inhibited by soy isoflavones in prostate cancer cells. Int J Cancer 124: 1675-1684.

Wang Y, Kreisberg JI, Ghosh PM (2007) Cross-talk between the androgen receptor and the phosphatidylinositol 3-kinase/Akt pathway in prostate cancer. Curr Cancer Drug Targets 7: 591-604.

Takeyama K, Mitsuzawa H, Nishitani C, Shimizu T, Sano H, et al. (2007) The 6-fluoro-8-methoxy quinolone gatifloxacin down-regulates interleukin-8 production in prostate cell line PC-3. Antimicrob Agents Chemother 51: 162-168.

Sun Y, St Clair DK, Fang F, Warren GW, Rangnekar VM, et al. (2007) The radiosensitization effect of parthenolide in prostate cancer cells is mediated by nuclear factor-kappaB inhibition and enhanced by the presence of PTEN. Mol Cancer Ther 6: 2477-2486.

Sun S, Tang Y, Lou X, Zhu L, Yang K, et al. (2007) UXT is a novel and essential cofactor in the NF-kappaB transcriptional enhanceosome. J Cell Biol 178: 231-244.

Sun HL, Liu YN, Huang YT, Pan SL, Huang DY, et al. (2007) YC-1 inhibits HIF-1 expression in prostate cancer cells: contribution of Akt/NF-kappaB signaling to HIF-1alpha accumulation during hypoxia. Oncogene 26: 3941-3951.

Son DJ, Lee JW, Lee YH, Song HS, Lee CK, et al. (2007) Therapeutic application of anti-arthritis, pain-releasing, and anti-cancer effects of bee venom and its constituent compounds. Pharmacol Ther 115: 246-270.

Sarkar FH, Adsule S, Li Y, Padhye S (2007) Back to the future: COX-2 inhibitors for chemoprevention and cancer therapy. Mini Rev Med Chem 7: 599-608.

660. Raffoul JJ, Banerjee S, Singh-Gupta V, Knoll ZE, Fite A, et al. (2007) Down-regulation of apurinic/apyrimidinic endonuclease 1/redox factor-1 expression by soy isoflavones enhances prostate cancer radiotherapy in vitro and in vivo. Cancer Res 67: 2141-2149.

Paule B, Terry S, Kheuang L, Soyeux P, Vacherot F, et al. (2007) The NF-kappaB/IL-6 pathway in metastatic androgen-independent prostate cancer: new therapeutic approaches? World J Urol 25: 477-489.

Ornstein DL, Zacharski LR (2007) Iron stimulates urokinase plasminogen activator expression and activates NF-kappa B in human prostate cancer cells. Nutr Cancer 58: 115-126.

Ogawa R, Kagiya G, Kodaki T, Fukuda S, Yamamoto K (2007) Construction of strong mammalian promoters by random cis-acting element elongation. Biotechniques 42: 628-633.

664. Nadiminty N, Chun JY, Hu Y, Dutt S, Lin X, et al. (2007) LIGHT, a member of the TNF superfamily, activates Stat3 mediated by NIK pathway. Biochem Biophys Res Commun 359: 379-384.

Momose I, Iijima M, Kawada M, Ikeda D (2007) A new proteasome inhibitor, TP-110, induces apoptosis in human prostate cancer PC-3 cells. Biosci Biotechnol Biochem 71: 1036-1043.

Maxwell PJ, Gallagher R, Seaton A, Wilson C, Scullin P, et al. (2007) HIF-1 and NF-kappaB-mediated upregulation of CXCR1 and CXCR2 expression promotes cell survival in hypoxic prostate cancer cells. Oncogene 26: 7333-7345.

Luo JL, Tan W, Ricono JM, Korchynskyi O, Zhang M, et al. (2007) Nuclear cytokine-activated IKKalpha controls prostate cancer metastasis by repressing Maspin. Nature 446: 690-694.

Lu PH, Chueh SC, Kung FL, Pan SL, Shen YC, et al. (2007) Ilimaquinone, a marine sponge metabolite, displays anticancer activity via GADD153-mediated pathway. Eur J Pharmacol 556: 45-54.

Li X, Chen D, Yin S, Meng Y, Yang H, et al. (2007) Maspin augments proteasome inhibitor-induced apoptosis in prostate cancer cells. J Cell Physiol 212: 298-306.

Kuefer R, Genze F, Zugmaier W, Hautmann RE, Rinnab L, et al. (2007) Antagonistic effects of sodium butyrate and N-(4-hydroxyphenyl)-retinamide on prostate cancer. Neoplasia 9: 246-253.

Kong D, Li Y, Wang Z, Banerjee S, Sarkar FH (2007) Inhibition of angiogenesis and invasion by 3,3'-diindolylmethane is mediated by the nuclear factor-kappaB downstream target genes MMP-9 and uPA that regulated bioavailability of vascular endothelial growth factor in prostate cancer. Cancer Res 67: 3310-3319.

Kikuno N, Shiina H, Urakami S, Kawamoto K, Hirata H, et al. (2007) Knockdown of astrocyte-elevated gene-1 inhibits prostate cancer progression through upregulation of FOXO3a activity. Oncogene 26: 7647-7655.

Kaur M, Agarwal R (2007) Transcription factors: molecular targets for prostate cancer intervention by phytochemicals. Curr Cancer Drug Targets 7: 355-367.

Jagla M, Feve M, Kessler P, Lapouge G, Erdmann E, et al. (2007) A splicing variant of the androgen receptor detected in a metastatic prostate cancer exhibits exclusively cytoplasmic actions. Endocrinology 148: 4334-4343.

He ML, Li A, Xu CS, Wang SL, Zhang MJ, et al. (2007) Mechanisms of antiprostate cancer by gum mastic: NF-kappaB signal as target. Acta Pharmacol Sin 28: 446-452.

Fradet V, Lessard L, Begin LR, Karakiewicz P, Masson AM, et al. (2004) Nuclear factor-kappaB nuclear localization is predictive of biochemical recurrence in patients with positive margin prostate cancer. Clin Cancer Res 10: 8460-8464.

Ling MT, Wang X, Ouyang XS, Xu K, Tsao SW, et al. (2003) Id-1 expression promotes cell survival through activation of NF-kappaB signalling pathway in prostate cancer cells. Oncogene 22: 4498-4508.

Kikuchi E, Horiguchi Y, Nakashima J, Kuroda K, Oya M, et al. (2003) Suppression of hormone-refractory prostate cancer by a novel nuclear factor kappaB inhibitor in nude mice. Cancer Res 63: 107-110.

Suh J, Payvandi F, Edelstein LC, Amenta PS, Zong WX, et al. (2002) Mechanisms of constitutive NF-kappaB activation in human prostate cancer cells. Prostate 52: 183-200.

Herrmann JL, Beham AW, Sarkiss M, Chiao PJ, Rands MT, et al. (1997) Bcl-2 suppresses apoptosis resulting from disruption of the NF-kappa B survival pathway. Exp Cell Res 237: 101-109.

Birnie R, Bryce SD, Roome C, Dussupt V, Droop A, et al. (2008) Gene expression profiling of human prostate cancer stem cells reveals a pro-inflammatory phenotype and the importance of extracellular matrix interactions. Genome Biol 9: R83.

Vykhovanets EV, Shukla S, MacLennan GT, Resnick MI, Carlsen H, et al. (2008) Molecular imaging of NF-kappaB in prostate tissue after systemic administration of IL-1 beta. Prostate 68: 34-41.

Nunez C, Cansino JR, Bethencourt F, Perez-Utrilla M, Fraile B, et al. (2008) TNF/IL-1/NIK/NF-kappa B transduction pathway: a comparative study in normal and pathological human prostate (benign hyperplasia and carcinoma). Histopathology 53: 166-176.

Vu HY, Juvekar A, Ghosh C, Ramaswami S, Le DH, et al. (2008) Proteasome inhibitors induce apoptosis of prostate cancer cells by inducing nuclear translocation of IkappaBalpha. Arch Biochem Biophys 475: 156-163.

Saad F, Markus R, Goessl C (2008) Targeting the receptor activator of nuclear factor-kappaB (RANK) ligand in prostate cancer bone metastases. BJU Int 101: 1071-1075.

Rettig MB, Heber D, An J, Seeram NP, Rao JY, et al. (2008) Pomegranate extract inhibits androgen-independent prostate cancer growth through a nuclear factor-kappaB-dependent mechanism. Mol Cancer Ther 7: 2662-2671.

Jin RJ, Lho Y, Connelly L, Wang Y, Yu X, et al. (2008) The nuclear factor-kappaB pathway controls the progression of prostate cancer to androgen-independent growth. Cancer Res 68: 6762-6769.

Cai Y, Lee YF, Li G, Liu S, Bao BY, et al. (2008) A new prostate cancer therapeutic approach: combination of androgen ablation with COX-2 inhibitor. Int J Cancer 123: 195-201.

Son DJ, Park MH, Chae SJ, Moon SO, Lee JW, et al. (2007) Inhibitory effect of snake venom toxin from Vipera lebetina turanica on hormone-refractory human prostate cancer cell growth: induction of apoptosis through inactivation of nuclear factor kappaB. Mol Cancer Ther 6: 675-683.

Singh RP, Agarwal R (2006) Prostate cancer chemoprevention by silibinin: bench to bedside. Mol Carcinog 45: 436-442.

Raffoul JJ, Wang Y, Kucuk O, Forman JD, Sarkar FH, et al. (2006) Genistein inhibits radiation-induced activation of NF-kappaB in prostate cancer cells promoting apoptosis and G2/M cell cycle arrest. BMC Cancer 6: 107.

Kwon O, Kim KA, Kim SO, Ha R, Oh WK, et al. (2006) NF-kappaB inhibition increases chemosensitivity to trichostatin A-induced cell death of Ki-Ras-transformed human prostate epithelial cells. Carcinogenesis 27: 2258-2268.

Huerta-Yepez S, Vega M, Garban H, Bonavida B (2006) Involvement of the TNF-alpha autocrine-paracrine loop, via NF-kappaB and YY1, in the regulation of tumor cell resistance to Fas-induced apoptosis. Clin Immunol 120: 297-309.

Ho SM, Leung YK, Chung I (2006) Estrogens and antiestrogens as etiological factors and therapeutics for prostate cancer. Ann N Y Acad Sci 1089: 177-193.

Armstrong K, Robson CN, Leung HY (2006) NF-kappaB activation upregulates fibroblast growth factor 8 expression in prostate cancer cells. Prostate 66: 1223-1234.

Agarwal R, Agarwal C, Ichikawa H, Singh RP, Aggarwal BB (2006) Anticancer potential of silymarin: from bench to bed side. Anticancer Res 26: 4457-4498.

Zerbini LF, Wang Y, Correa RG, Cho JY, Libermann TA (2005) Blockage of NF-kappaB induces serine 15 phosphorylation of mutant p53 by JNK kinase in prostate cancer cells. Cell Cycle 4: 1247-1253.

Reddy GP, Barrack ER, Dou QP, Menon M, Pelley R, et al. (2006) Regulatory processes affecting androgen receptor expression, stability, and function: potential targets to treat hormone-refractory prostate cancer. J Cell Biochem 98: 1408-1423.

Nishimura K, Takayama H, Nakayama M, Nonomura N, Okuyama A (2006) [Molecular-targeted therapy for hormone-refractory prostate cancer]. Hinyokika Kiyo 52: 487-490.

Nadiminty N, Lou W, Lee SO, Mehraein-Ghomi F, Kirk JS, et al. (2006) Prostate-specific antigen modulates genes involved in bone remodeling and induces osteoblast differentiation of human osteosarcoma cell line SaOS-2. Clin Cancer Res 12: 1420-1430.

Mori A, Lehmann S, O'Kelly J, Kumagai T, Desmond JC, et al. (2006) Capsaicin, a component of red peppers, inhibits the growth of androgen-independent, p53 mutant prostate cancer cells. Cancer Res 66: 3222-3229.

Misra UK, Deedwania R, Pizzo SV (2006) Activation and cross-talk between Akt, NF-kappaB, and unfolded protein response signaling in 1-LN prostate cancer cells consequent to ligation of cell surface-associated GRP78. J Biol Chem 281: 13694-13707.

Ma Y, Lakshmikanthan V, Lewis RW, Kumar MV (2006) Sensitization of TRAIL-resistant cells by inhibition of heat shock protein 90 with low-dose geldanamycin. Mol Cancer Ther 5: 170-178.

Li Z, Chen Y, Cao D, Wang Y, Chen G, et al. (2006) Glucocorticoid up-regulates transforming growth factor-beta (TGF-beta) type II receptor and enhances TGF-beta signaling in human prostate cancer PC-3 cells. Endocrinology 147: 5259-5267.

Lessard L, Karakiewicz PI, Bellon-Gagnon P, Alam-Fahmy M, Ismail HA, et al. (2006) Nuclear localization of nuclear factor-kappaB p65 in primary prostate tumors is highly predictive of pelvic lymph node metastases. Clin Cancer Res 12: 5741-5745.

Labbozzetta M, Notarbartolo M, Poma P, Giannitrapani L, Cervello M, et al. (2006) Significance of autologous interleukin-6 production in the HA22T/VGH cell model of hepatocellular carcinoma. Ann N Y Acad Sci 1089: 268-275.

Kim JH, Xu C, Keum YS, Reddy B, Conney A, et al. (2006) Inhibition of EGFR signaling in human prostate cancer PC-3 cells by combination treatment with beta-phenylethyl isothiocyanate and curcumin. Carcinogenesis 27: 475-482.

Kao JJ (2006) The NF-kappaB inhibitor pyrrolidine dithiocarbamate blocks IL-1beta induced hyaluronan synthase 1 (HAS1) mRNA transcription, pointing at NF-kappaB dependence of the gene HAS1. Exp Gerontol 41: 641-647.

Josson S, Xu Y, Fang F, Dhar SK, St Clair DK, et al. (2006) RelB regulates manganese superoxide dismutase gene and resistance to ionizing radiation of prostate cancer cells. Oncogene 25: 1554-1559.

Jiang Z, Clemens PR (2006) Cellular caspase-8-like inhibitory protein (cFLIP) prevents inhibition of muscle cell differentiation induced by cancer cells. FASEB J 20: 2570-2572.

Jiang BH, Liu LZ, Schafer R, Flynn DC, Barnett JB (2006) A novel role for 3, 4-dichloropropionanilide (DCPA) in the inhibition of prostate cancer cell migration, proliferation, and hypoxia-inducible factor 1alpha expression. BMC Cancer 6: 204.

Hwang YS, Hodge JC, Sivapurapu N, Lindholm PF (2006) Lysophosphatidic acid stimulates PC-3 prostate cancer cell Matrigel invasion through activation of RhoA and NF-kappaB activity. Mol Carcinog 45: 518-529.

Hughes-Fulford M, Li CF, Boonyaratanakornkit J, Sayyah S (2006) Arachidonic acid activates phosphatidylinositol 3-kinase signaling and induces gene expression in prostate cancer. Cancer Res 66: 1427-1433.

Huangfu WC, Omori E, Akira S, Matsumoto K, Ninomiya-Tsuji J (2006) Osmotic stress activates the TAK1-JNK pathway while blocking TAK1-mediated NF-kappaB activation: TAO2 regulates TAK1 pathways. J Biol Chem 281: 28802-28810.

Hermani A, De Servi B, Medunjanin S, Tessier PA, Mayer D (2006) S100A8 and S100A9 activate MAP kinase and NF-kappaB signaling pathways and trigger translocation of RAGE in human prostate cancer cells. Exp Cell Res 312: 184-197.

Hassan F, Islam S, Tumurkhuu G, Naiki Y, Koide N, et al. (2006) Intracellular expression of toll-like receptor 4 in neuroblastoma cells and their unresponsiveness to lipopolysaccharide. BMC Cancer 6: 281.

Harkonen PL, Vaananen HK (2006) Monocyte-macrophage system as a target for estrogen and selective estrogen receptor modulators. Ann N Y Acad Sci 1089: 218-227.

Guo H, Yu JH, Chen K, Ye ZQ, Liu GC (2006) [Curcumin-induced apoptosis in androgen-dependent prostate cancer cell line LNCaP in vitro]. Zhonghua Nan Ke Xue 12: 141-144.

Guo H, Yu JH, Chen K, Ye ZQ (2006) [Curcumin-induced the expression of inhibitor kappaBalpha protein in human prostate cancer cells]. Zhonghua Wai Ke Za Zhi 44: 1256-1259.

Girvan AC, Teng Y, Casson LK, Thomas SD, Juliger S, et al. (2006) AGRO100 inhibits activation of nuclear factor-kappaB (NF-kappaB) by forming a complex with NF-kappaB essential modulator (NEMO) and nucleolin. Mol Cancer Ther 5: 1790-1799.

Dougall WC, Chaisson M (2006) The RANK/RANKL/OPG triad in cancer-induced bone diseases. Cancer Metastasis Rev 25: 541-549.

Zhi H, Yang XJ, Kuhnmuench J, Berg T, Thill R, et al. (2009) SmgGDS is up-regulated in prostate carcinoma and promotes tumour phenotypes in prostate cancer cells. J Pathol 217: 389-397.

Vykhovanets EV, Shukla S, Maclennan GT, Vykhovanets OV, Bodner DR, et al. (2009) Il-1beta-induced post-transition effect of NF-kappaB provides time-dependent wave of signals for initial phase of intrapostatic inflammation. Prostate.

Singh-Gupta V, Zhang H, Banerjee S, Kong D, Raffoul JJ, et al. (2009) Radiation-induced HIF-1alpha cell survival pathway is inhibited by soy isoflavones in prostate cancer cells. Int J Cancer 124: 1675-1684.

Peant B, Diallo JS, Dufour F, Le Page C, Delvoye N, et al. (2009) Over-expression of IkappaB-kinase-epsilon (IKKepsilon/IKKi) induces secretion of inflammatory cytokines in prostate cancer cell lines. Prostate.

Narayanan NK, Nargi D, Horton L, Reddy BS, Bosland MC, et al. (2009) Inflammatory processes of prostate tissue microenvironment drive rat prostate carcinogenesis: preventive effects of celecoxib. Prostate 69: 133-141.

Koeneman KS (2009) Receptor activator of NF-kappaB ligand (RANKL) expression is associated with epithelial to mesenchymal transition in human prostate cancer cells Odero-Marah VA, Wang R, Chu G, Zayzafoon M, Xu J, Shi C, Marshall FF, Zhau HE, Chung LW, Molecular Urology and Therapeutics Program, Department of Urology and Winship Cancer Institute, Emory University School of Medicine, Atlanta, GA. Urol Oncol 27: 111-112.

Hung SH, Shen KH, Wu CH, Liu CL, Shih YW (2009) alpha-Mangostin Suppresses PC-3 Human Prostate Carcinoma Cell Metastasis by Inhibiting Matrix Metalloproteinase-2/9 and Urokinase-Plasminogen Expression through the JNK Signaling Pathway. J Agric Food Chem.

Huerta-Yepez S, Vega M, Escoto-Chavez SE, Murdock B, Sakai T, et al. (2009) Nitric oxide sensitizes tumor cells to TRAIL-induced apoptosis via inhibition of the DR5 transcription repressor Yin Yang 1. Nitric Oxide 20: 39-52.

Graham TR, Odero-Marah VA, Chung LW, Agrawal KC, Davis R, et al. (2009) PI3K/Akt-dependent transcriptional regulation and activation of BMP-2-Smad signaling by NF-kappaB in metastatic prostate cancer cells. Prostate 69: 168-180.

Fernandez-Martinez AB, Bajo AM, Sanchez-Chapado M, Prieto JC, Carmena MJ (2009) Vasoactive intestinal peptide behaves as a pro-metastatic factor in human prostate cancer cells. Prostate.

Deeb D, Gao X, Jiang H, Dulchavsky SA, Gautam SC (2009) Oleanane Triterpenoid CDDO-Me inhibits growth and induces apoptosis in prostate cancer cells by independently targeting pro-survival Akt and mTOR. Prostate.

Dangi-Garimella S, Yun J, Eves EM, Newman M, Erkeland SJ, et al. (2009) Raf kinase inhibitory protein suppresses a metastasis signalling cascade involving LIN28 and let-7. EMBO J.

Chinnakannu K, Chen D, Li Y, Wang Z, Dou QP, et al. (2009) Cell cycle-dependent effects of 3,3'-diindolylmethane on proliferation and apoptosis of prostate cancer cells. J Cell Physiol 219: 94-99.

Cai Y, Wang J, Li R, Ayala G, Ittmann M, et al. (2009) GGAP2/PIKE-a directly activates both the Akt and nuclear factor-kappaB pathways and promotes prostate cancer progression. Cancer Res 69: 819-827.

Zheng X, Chang RL, Cui XX, Avila G, Huang MT, et al. (2008) Inhibition of NF-kappaB by (E)3-[(4-methylphenyl)-sulfonyl]-2-propenenitrile (BAY11-7082; BAY) is associated with enhanced 12-O-tetradecanoylphorbol-13-acetate-induced growth suppression and apoptosis in human prostate cancer PC-3 cells. Int J Oncol 32: 257-264.

Zhau HE, Odero-Marah V, Lue HW, Nomura T, Wang R, et al. (2008) Epithelial to mesenchymal transition (EMT) in human prostate cancer: lessons learned from ARCaP model. Clin Exp Metastasis 25: 601-610.

Zhang X, Huang X, Olumi AF (2008) Repression of NF-kappaB and activation of AP-1 enhance apoptosis in prostate cancer cells. Int J Cancer 124: 1980-1989.

You Z, Dong Y, Kong X, Beckett LA, Gandour-Edwards R, et al. (2008) Midkine is a NF-kappaB-inducible gene that supports prostate cancer cell survival. BMC Med Genomics 1: 6.

691. Yap WN, Chang PN, Han HY, Lee DT, Ling MT, et al. (2008) Gamma-tocotrienol suppresses prostate cancer cell proliferation and invasion through multiple-signalling pathways. Br J Cancer 99: 1832-1841.

Yan X, Shen H, Jiang H, Zhang C, Hu D, et al. (2008) External Qi of Yan Xin Qigong induces G2/M arrest and apoptosis of androgen-independent prostate cancer cells by inhibiting Akt and NF-kappa B pathways. Mol Cell Biochem 310: 227-234.

Xu Y, Fang F, St Clair DK, Sompol P, Josson S, et al. (2008) SN52, a novel nuclear factor-kappaB inhibitor, blocks nuclear import of RelB:p52 dimer and sensitizes prostate cancer cells to ionizing radiation. Mol Cancer Ther 7: 2367-2376.

Win HY, Acevedo-Duncan M (2008) Atypical protein kinase C phosphorylates IKKalphabeta in transformed non-malignant and malignant prostate cell survival. Cancer Lett 270: 302-311.

Wilson C, Scullin P, Worthington J, Seaton A, Maxwell P, et al. (2008) Dexamethasone potentiates the antiangiogenic activity of docetaxel in castration-resistant prostate cancer. Br J Cancer 99: 2054-2064.

Wilson C, Purcell C, Seaton A, Oladipo O, Maxwell PJ, et al. (2008) Chemotherapy-induced CXC-chemokine/CXC-chemokine receptor signaling in metastatic prostate cancer cells confers resistance to oxaliplatin through potentiation of nuclear factor-kappaB transcription and evasion of apoptosis. J Pharmacol Exp Ther 327: 746-759.

Tsui KH, Feng TH, Hsieh WC, Chang PL, Juang HH (2008) Expression of interleukin-6 is downregulated by 17-(allylamino)-17-demethoxygeldanamycin in human prostatic carcinoma cells. Acta Pharmacol Sin 29: 1334-1341.

Song JH, Kandasamy K, Kraft AS (2008) ABT-737 induces expression of the death receptor 5 and sensitizes human cancer cells to TRAIL-induced apoptosis. J Biol Chem 283: 25003-25013.

Solomon LA, Ali S, Banerjee S, Munkarah AR, Morris RT, et al. (2008) Sensitization of ovarian cancer cells to cisplatin by genistein: the role of NF-kappaB. J Ovarian Res 1: 9.

Smith MR (2008) Osteoclast targeted therapy for prostate cancer: bisphosphonates and beyond. Urol Oncol 26: 420-425.

Singh RK, Lange TS, Kim KK, Shaw SK, Brard L (2008) A novel indole ethyl isothiocyanate (7Me-IEITC) with anti-proliferative and pro-apoptotic effects on platinum-resistant human ovarian cancer cells. Gynecol Oncol 109: 240-249.

Shukla S, Gupta S (2008) Apigenin-induced prostate cancer cell death is initiated by reactive oxygen species and p53 activation. Free Radic Biol Med 44: 1833-1845.

Shaikh IA, Brown I, Schofield AC, Wahle KW, Heys SD (2008) Docosahexaenoic acid enhances the efficacy of docetaxel in prostate cancer cells by modulation of apoptosis: the role of genes associated with the NF-kappaB pathway. Prostate 68: 1635-1646.

Royuela M, Rodriguez-Berriguete G, Fraile B, Paniagua R (2008) TNF-alpha/IL-1/NF-kappaB transduction pathway in human cancer prostate. Histol Histopathol 23: 1279-1290.

Rabi T, Shukla S, Gupta S (2008) Betulinic acid suppresses constitutive and TNFalpha-induced NF-kappaB activation and induces apoptosis in human prostate carcinoma PC-3 cells. Mol Carcinog 47: 964-973.

Perez-Martinez FC, Alonso V, Sarasa JL, Manzarbeitia F, Vela-Navarrete R, et al. (2008) Receptor activator of nuclear factor-kappaB ligand (RANKL) as a novel prognostic marker in prostate carcinoma. Histol Histopathol 23: 709-715.

Penna G, Fibbi B, Amuchastegui S, Corsiero E, Laverny G, et al. (2008) The vitamin D receptor agonist elocalcitol inhibits IL-8-dependent benign prostatic hyperplasia stromal cell proliferation and inflammatory response by targeting the RhoA/Rho kinase and NF-kB pathways. Prostate.

Papadopoulou N, Charalampopoulos I, Anagnostopoulou V, Konstantinidis G, Foller M, et al. (2008) Membrane androgen receptor activation triggers down-regulation of PI-3K/Akt/NF-kappaB activity and induces apoptotic responses via Bad, FasL and caspase-3 in DU145 prostate cancer cells. Mol Cancer 7: 88.

Odero-Marah VA, Wang R, Chu G, Zayzafoon M, Xu J, et al. (2008) Receptor activator of NF-kappaB Ligand (RANKL) expression is associated with epithelial to mesenchymal transition in human prostate cancer cells. Cell Res 18: 858-870.

Niciforovic A, Djordjevic J, Adzic M, Vucic V, Mitrasinovic PM, et al. (2008) Experimental and systems biology studies of the molecular basis for the radioresistance of prostate carcinoma cells. Ann Biomed Eng 36: 831-838.

Liu Y, Mo JQ, Hu Q, Boivin G, Levin L, et al. (2008) Targeted overexpression of vav3 oncogene in prostatic epithelium induces nonbacterial prostatitis and prostate cancer. Cancer Res 68: 6396-6406.

Lee SY, Yuk DY, Song HS, Yoon do Y, Jung JK, et al. (2008) Growth inhibitory effects of obovatol through induction of apoptotic cell death in prostate and colon cancer by blocking of NF-kappaB. Eur J Pharmacol 582: 17-25.

Lee SO, Pinder E, Chun JY, Lou W, Sun M, et al. (2008) Interleukin-4 stimulates androgen-independent growth in LNCaP human prostate cancer cells. Prostate 68: 85-91.

Lai TH, Fong YC, Fu WM, Yang RS, Tang CH (2008) Osteoblasts-derived BMP-2 enhances the motility of prostate cancer cells via activation of integrins. Prostate 68: 1341-1353.

Kwon O, Kim KA, He L, Jung M, Jeong SJ, et al. (2008) Complex formation of p65/RelA with nuclear Akt1 for enhanced transcriptional activation of NF-kappaB. Biochem Biophys Res Commun 365: 771-776.

Ko S, Shi L, Kim S, Song CS, Chatterjee B (2008) Interplay of nuclear factor-kappaB and B-myb in the negative regulation of androgen receptor expression by tumor necrosis factor alpha. Mol Endocrinol 22: 273-286.

Kikuno N, Shiina H, Urakami S, Kawamoto K, Hirata H, et al. (2008) Genistein mediated histone acetylation and demethylation activates tumor suppressor genes in prostate cancer cells. Int J Cancer 123: 552-560.

Jiang S, Zu Y, Fu Y, Zhang Y, Efferth T (2008) Activation of the mitochondria-driven pathway of apoptosis in human PC-3 prostate cancer cells by a novel hydrophilic paclitaxel derivative, 7-xylosyl-10-deacetylpaclitaxel. Int J Oncol 33: 103-111.

Hui H, Fernando MA, Heaney AP (2008) The alpha1-adrenergic receptor antagonist doxazosin inhibits EGFR and NF-kappaB signalling to induce breast cancer cell apoptosis. Eur J Cancer 44: 160-166.

Hafeez BB, Siddiqui IA, Asim M, Malik A, Afaq F, et al. (2008) A dietary anthocyanidin delphinidin induces apoptosis of human prostate cancer PC3 cells in vitro and in vivo: involvement of nuclear factor-kappaB signaling. Cancer Res 68: 8564-8572.

Golovine K, Uzzo RG, Makhov P, Crispen PL, Kunkle D, et al. (2008) Depletion of intracellular zinc increases expression of tumorigenic cytokines VEGF, IL-6 and IL-8 in prostate cancer cells via NF-kappaB-dependent pathway. Prostate 68: 1443-1449.

Golovine K, Makhov P, Uzzo RG, Shaw T, Kunkle D, et al. (2008) Overexpression of the zinc uptake transporter hZIP1 inhibits nuclear factor-kappaB and reduces the malignant potential of prostate cancer cells in vitro and in vivo. Clin Cancer Res 14: 5376-5384.

Fenton JI, Birmingham JM, Hursting SD, Hord NG (2008) Adiponectin blocks multiple signaling cascades associated with leptin-induced cell proliferation in Apc Min/+ colon epithelial cells. Int J Cancer 122: 2437-2445.

dos Santos NR, Williame M, Gachet S, Cormier F, Janin A, et al. (2008) RelB-dependent stromal cells promote T-cell leukemogenesis. PLoS ONE 3: e2555.

Diallo JS, Betton B, Parent N, Peant B, Lessard L, et al. (2008) Enhanced killing of androgen-independent prostate cancer cells using inositol hexakisphosphate in combination with proteasome inhibitors. Br J Cancer 99: 1613-1622.

Deeb D, Gao X, Dulchavsky SA, Gautam SC (2008) CDDO-Me inhibits proliferation, induces apoptosis, down-regulates Akt, mTOR, NF-kappaB and NF-kappaB-regulated antiapoptotic and proangiogenic proteins in TRAMP prostate cancer cells. J Exp Ther Oncol 7: 31-39.

Dan HC, Cooper MJ, Cogswell PC, Duncan JA, Ting JP, et al. (2008) Akt-dependent regulation of NF-{kappa}B is controlled by mTOR and Raptor in association with IKK. Genes Dev 22: 1490-1500.

Barabutis N, Schally AV (2008) Antioxidant activity of growth hormone-releasing hormone antagonists in LNCaP human prostate cancer line. Proc Natl Acad Sci U S A 105: 20470-20475.

Banerjee S, Li Y, Wang Z, Sarkar FH (2008) Multi-targeted therapy of cancer by genistein. Cancer Lett 269: 226-242.

Ash SC, Yang DQ, Britt DE (2008) LYRIC/AEG-1 overexpression modulates BCCIPalpha protein levels in prostate tumor cells. Biochem Biophys Res Commun 371: 333-338.

Armstrong AP, Miller RE, Jones JC, Zhang J, Keller ET, et al. (2008) RANKL acts directly on RANK-expressing prostate tumor cells and mediates migration and expression of tumor metastasis genes. Prostate 68: 92-104.

Andrzejewski T, Deeb D, Gao X, Danyluk A, Arbab AS, et al. (2008) Therapeutic efficacy of curcumin/TRAIL combination regimen for hormone-refractory prostate cancer. Oncol Res 17: 257-267.

Domingo-Domenech J, Oliva C, Rovira A, Codony-Servat J, Bosch M, et al. (2006) Interleukin 6, a nuclear factor-kappaB target, predicts resistance to docetaxel in hormone-independent prostate cancer and nuclear factor-kappaB inhibition by PS-1145 enhances docetaxel antitumor activity. Clin Cancer Res 12: 5578-5586.

Diallo JS, Peant B, Lessard L, Delvoye N, Le Page C, et al. (2006) An androgen-independent androgen receptor function protects from inositol hexakisphosphate toxicity in the PC3/PC3(AR) prostate cancer cell lines. Prostate 66: 1245-1256.

Chen G, Sircar K, Aprikian A, Potti A, Goltzman D, et al. (2006) Expression of RANKL/RANK/OPG in primary and metastatic human prostate cancer as markers of disease stage and functional regulation. Cancer 107: 289-298.

Bhuiyan MM, Li Y, Banerjee S, Ahmed F, Wang Z, et al. (2006) Down-regulation of androgen receptor by 3,3'-diindolylmethane contributes to inhibition of cell proliferation and induction of apoptosis in both hormone-sensitive LNCaP and insensitive C4-2B prostate cancer cells. Cancer Res 66: 10064-10072.

Barve V, Ahmed F, Adsule S, Banerjee S, Kulkarni S, et al. (2006) Synthesis, molecular characterization, and biological activity of novel synthetic derivatives of chromen-4-one in human cancer cells. J Med Chem 49: 3800-3808.

Bao BY, Yao J, Lee YF (2006) 1alpha, 25-dihydroxyvitamin D3 suppresses interleukin-8-mediated prostate cancer cell angiogenesis. Carcinogenesis 27: 1883-1893.

Xu C, Shen G, Chen C, Gelinas C, Kong AN (2005) Suppression of NF-kappaB and NF-kappaB-regulated gene expression by sulforaphane and PEITC through IkappaBalpha, IKK pathway in human prostate cancer PC-3 cells. Oncogene 24: 4486-4495.

Whang PG, Schwarz EM, Gamradt SC, Dougall WC, Lieberman JR (2005) The effects of RANK blockade and osteoclast depletion in a model of pure osteoblastic prostate cancer metastasis in bone. J Orthop Res 23: 1475-1483.

Wang G, Silva J, Krishnamurthy K, Tran E, Condie BG, et al. (2005) Direct binding to ceramide activates protein kinase Czeta before the formation of a pro-apoptotic complex with PAR-4 in differentiating stem cells. J Biol Chem 280: 26415-26424.

Syrovets T, Gschwend JE, Buchele B, Laumonnier Y, Zugmaier W, et al. (2005) Inhibition of IkappaB kinase activity by acetyl-boswellic acids promotes apoptosis in androgen-independent PC-3 prostate cancer cells in vitro and in vivo. J Biol Chem 280: 6170-6180.

Shukla S, Maclennan GT, Marengo SR, Resnick MI, Gupta S (2005) Constitutive activation of P I3 K-Akt and NF-kappaB during prostate cancer progression in autochthonous transgenic mouse model. Prostate 64: 224-239.

Li Y, Chinni SR, Sarkar FH (2005) Selective growth regulatory and pro-apoptotic effects of DIM is mediated by AKT and NF-kappaB pathways in prostate cancer cells. Front Biosci 10: 236-243.

Li Y, Ahmed F, Ali S, Philip PA, Kucuk O, et al. (2005) Inactivation of nuclear factor kappaB by soy isoflavone genistein contributes to increased apoptosis induced by chemotherapeutic agents in human cancer cells. Cancer Res 65: 6934-6942.

Lessard L, Begin LR, Gleave ME, Mes-Masson AM, Saad F (2005) Nuclear localisation of nuclear factor-kappaB transcription factors in prostate cancer: an immunohistochemical study. Br J Cancer 93: 1019-1023.

Lee SO, Lou W, Nadiminty N, Lin X, Gao AC (2005) Requirement for NF-(kappa)B in interleukin-4-induced androgen receptor activation in prostate cancer cells. Prostate 64: 160-167.

Le Page C, Koumakpayi IH, Lessard L, Saad F, Mes-Masson AM (2005) Independent role of phosphoinositol-3-kinase (PI3K) and casein kinase II (CK-2) in EGFR and Her-2-mediated constitutive NF-kappaB activation in prostate cancer cells. Prostate 65: 306-315.

Le Page C, Koumakpayi IH, Lessard L, Mes-Masson AM, Saad F (2005) EGFR and Her-2 regulate the constitutive activation of NF-kappaB in PC-3 prostate cancer cells. Prostate 65: 130-140.

Kuroda K, Horiguchi Y, Nakashima J, Kikuchi E, Kanao K, et al. (2005) Prevention of cancer cachexia by a novel nuclear factor {kappa}B inhibitor in prostate cancer. Clin Cancer Res 11: 5590-5594.

Kukreja P, Abdel-Mageed AB, Mondal D, Liu K, Agrawal KC (2005) Up-regulation of CXCR4 expression in PC-3 cells by stromal-derived factor-1alpha (CXCL12) increases endothelial adhesion and transendothelial migration: role of MEK/ERK signaling pathway-dependent NF-kappaB activation. Cancer Res 65: 9891-9898.

Kim KM, Song JJ, An JY, Kwon YT, Lee YJ (2005) Pretreatment of acetylsalicylic acid promotes tumor necrosis factor-related apoptosis-inducing ligand-induced apoptosis by down-regulating BCL-2 gene expression. J Biol Chem 280: 41047-41056.

Kim JH, Kim B, Cai L, Choi HJ, Ohgi KA, et al. (2005) Transcriptional regulation of a metastasis suppressor gene by Tip60 and beta-catenin complexes. Nature 434: 921-926.

Kim BY, Kim KA, Kwon O, Kim SO, Kim MS, et al. (2005) NF-kappaB inhibition radiosensitizes Ki-Ras-transformed cells to ionizing radiation. Carcinogenesis 26: 1395-1403.

Keller ET, Fu Z, Brennan M (2005) The biology of a prostate cancer metastasis suppressor protein: Raf kinase inhibitor protein. J Cell Biochem 94: 273-278.

Kashfi K, Rigas B (2005) Molecular targets of nitric-oxide-donating aspirin in cancer. Biochem Soc Trans 33: 701-704.

Jin F, Liu X, Zhou Z, Yue P, Lotan R, et al. (2005) Activation of nuclear factor-kappaB contributes to induction of death receptors and apoptosis by the synthetic retinoid CD437 in DU145 human prostate cancer cells. Cancer Res 65: 6354-6363.

Inoue H, Nishimura K, Oka D, Nakai Y, Shiba M, et al. (2005) Prostate cancer mediates osteoclastogenesis through two different pathways. Cancer Lett 223: 121-128.

Huang YT, Pan SL, Guh JH, Chang YL, Lee FY, et al. (2005) YC-1 suppresses constitutive nuclear factor-kappaB activation and induces apoptosis in human prostate cancer cells. Mol Cancer Ther 4: 1628-1635.

Gupta SV, McGowen RM, Callewaert DM, Brown TR, Li Y, et al. (2005) Quantitative chemiluminescent immunoassay for NF-kappaB-DNA binding activity. J Immunoassay Immunochem 26: 125-143.

Guo CY, Wang XF, Xu KX, Dong JQ, Huang XB, et al. (2005) [Inhibitory effect of doxazosin on the growth of transplanted tumor of prostate cancer cell PC-3 in nude mice]. Beijing Da Xue Xue Bao 37: 273-277.

Gunawardena K, Campbell LD, Meikle AW (2005) Antiandrogen-like actions of an antioxidant on survivin, Bcl-2 and PSA in human prostate cancer cells. Cancer Detect Prev 29: 389-395.

Ghosh AK, Steele R, Ray RB (2005) c-myc Promoter-binding protein 1 (MBP-1) regulates prostate cancer cell growth by inhibiting MAPK pathway. J Biol Chem 280: 14325-14330.

Gabai VL, Budagova KR, Sherman MY (2005) Increased expression of the major heat shock protein Hsp72 in human prostate carcinoma cells is dispensable for their viability but confers resistance to a variety of anticancer agents. Oncogene 24: 3328-3338.

Floryk D, Huberman E (2005) Differentiation of androgen-independent prostate cancer PC-3 cells is associated with increased nuclear factor-kappaB activity. Cancer Res 65: 11588-11596.

Fernando MA, Heaney AP (2005) Alpha1-adrenergic receptor antagonists: novel therapy for pituitary adenomas. Mol Endocrinol 19: 3085-3096.

Fan S, Gao M, Meng Q, Laterra JJ, Symons MH, et al. (2005) Role of NF-kappaB signaling in hepatocyte growth factor/scatter factor-mediated cell protection. Oncogene 24: 1749-1766.

Fahy BN, Schlieman MG, Mortenson MM, Virudachalam S, Bold RJ (2005) Targeting BCL-2 overexpression in various human malignancies through NF-kappaB inhibition by the proteasome inhibitor bortezomib. Cancer Chemother Pharmacol 56: 46-54.

Domingo-Domenech J, Mellado B, Ferrer B, Truan D, Codony-Servat J, et al. (2005) Activation of nuclear factor-kappaB in human prostate carcinogenesis and association to biochemical relapse. Br J Cancer 93: 1285-1294.

Dobrovolskaia MA, Kozlov SV (2005) Inflammation and cancer: when NF-kappaB amalgamates the perilous partnership. Curr Cancer Drug Targets 5: 325-344.

Dilley WG, Kalyanaraman S, Verma S, Cobb JP, Laramie JM, et al. (2005) Global gene expression in neuroendocrine tumors from patients with the MEN1 syndrome. Mol Cancer 4: 9.

Deeb DD, Jiang H, Gao X, Divine G, Dulchavsky SA, et al. (2005) Chemosensitization of hormone-refractory prostate cancer cells by curcumin to TRAIL-induced apoptosis. J Exp Ther Oncol 5: 81-91.

Zhang J, Lu Y, Dai J, Yao Z, Kitazawa R, et al. (2004) In vivo real-time imaging of TGF-beta-induced transcriptional activation of the RANK ligand gene promoter in intraosseous prostate cancer. Prostate 59: 360-369.

Zelivianski S, Glowacki R, Lin MF (2004) Transcriptional activation of the human prostatic acid phosphatase gene by NF-kappaB via a novel hexanucleotide-binding site. Nucleic Acids Res 32: 3566-3580.

Xiao W, Hodge DR, Wang L, Yang X, Zhang X, et al. (2004) Co-operative functions between nuclear factors NFkappaB and CCAT/enhancer-binding protein-beta (C/EBP-beta) regulate the IL-6 promoter in autocrine human prostate cancer cells. Prostate 61: 354-370.

Wang G, Reed E, Li QQ (2004) Apoptosis in prostate cancer: progressive and therapeutic implications (Review). Int J Mol Med 14: 23-34.

Vayalil PK, Katiyar SK (2004) Treatment of epigallocatechin-3-gallate inhibits matrix metalloproteinases-2 and -9 via inhibition of activation of mitogen-activated protein kinases, c-jun and NF-kappaB in human prostate carcinoma DU-145 cells. Prostate 59: 33-42.

Sweeney C, Li L, Shanmugam R, Bhat-Nakshatri P, Jayaprakasan V, et al. (2004) Nuclear factor-kappaB is constitutively activated in prostate cancer in vitro and is overexpressed in prostatic intraepithelial neoplasia and adenocarcinoma of the prostate. Clin Cancer Res 10: 5501-5507.

Suh J, Rabson AB (2004) NF-kappaB activation in human prostate cancer: important mediator or epiphenomenon? J Cell Biochem 91: 100-117.

Shukla S, MacLennan GT, Fu P, Patel J, Marengo SR, et al. (2004) Nuclear factor-kappaB/p65 (Rel A) is constitutively activated in human prostate adenocarcinoma and correlates with disease progression. Neoplasia 6: 390-400.

Shukla S, Gupta S (2004) Molecular mechanisms for apigenin-induced cell-cycle arrest and apoptosis of hormone refractory human prostate carcinoma DU145 cells. Mol Carcinog 39: 114-126.

Shukla S, Gupta S (2004) Suppression of constitutive and tumor necrosis factor alpha-induced nuclear factor (NF)-kappaB activation and induction of apoptosis by apigenin in human prostate carcinoma PC-3 cells: correlation with down-regulation of NF-kappaB-responsive genes. Clin Cancer Res 10: 3169-3178.

Shimada K, Nakamura M, Ishida E, Kishi M, Matsuyoshi S, et al. (2004) The molecular mechanism of sensitization to Fas-mediated apoptosis by 2-methoxyestradiol in PC3 prostate cancer cells. Mol Carcinog 39: 1-9.

Sarkar FH, Li Y (2004) Indole-3-carbinol and prostate cancer. J Nutr 134: 3493S-3498S.

Ross JS, Kallakury BV, Sheehan CE, Fisher HA, Kaufman RP, Jr., et al. (2004) Expression of nuclear factor-kappa B and I kappa B alpha proteins in prostatic adenocarcinomas: correlation of nuclear factor-kappa B immunoreactivity with disease recurrence. Clin Cancer Res 10: 2466-2472.

Rege YD, Rangnekar VM (2004) Molecular therapy intervention prospects in prostate cancer. Curr Pharm Des 10: 523-530.

Raj GV, Sekula JA, Guo R, Madden JF, Daaka Y (2004) Lysophosphatidic acid promotes survival of androgen-insensitive prostate cancer PC3 cells via activation of NF-kappaB. Prostate 61: 105-113.

Penninkhof F, Grootegoed JA, Blok LJ (2004) Identification of REPS2 as a putative modulator of NF-kappaB activity in prostate cancer cells. Oncogene 23: 5607-5615.

Pandey M, Bajaj GD, Rath PC (2004) Induction of the interferon-inducible RNA-degrading enzyme, RNase L, by stress-inducing agents in the human cervical carcinoma cells. RNA Biol 1: 21-27.

Newcomb EW (2004) Flavopiridol: pleiotropic biological effects enhance its anti-cancer activity. Anticancer Drugs 15: 411-419.

McEleny K, Coffey R, Morrissey C, Fitzpatrick JM, Watson RW (2004) Caffeic acid phenethyl ester-induced PC-3 cell apoptosis is caspase-dependent and mediated through the loss of inhibitors of apoptosis proteins. BJU Int 94: 402-406.

McCarty MF (2004) Targeting multiple signaling pathways as a strategy for managing prostate cancer: multifocal signal modulation therapy. Integr Cancer Ther 3: 349-380.

Lu T, Burdelya LG, Swiatkowski SM, Boiko AD, Howe PH, et al. (2004) Secreted transforming growth factor beta2 activates NF-kappaB, blocks apoptosis, and is essential for the survival of some tumor cells. Proc Natl Acad Sci U S A 101: 7112-7117.

Liu CA, Wang MJ, Chi CW, Wu CW, Chen JY (2004) Overexpression of rho effector rhotekin confers increased survival in gastric adenocarcinoma. J Biomed Sci 11: 661-670.

Lee JY, Je JH, Jung KJ, Yu BP, Chung HY (2004) Induction of endothelial iNOS by 4-hydroxyhexenal through NF-kappaB activation. Free Radic Biol Med 37: 539-548.

Lee EC, Tenniswood M (2004) Programmed cell death and survival pathways in prostate cancer cells. Arch Androl 50: 27-32.

Kim HY, Yu R, Kim JS, Kim YK, Sung MK (2004) Antiproliferative crude soy saponin extract modulates the expression of IkappaBalpha, protein kinase C, and cyclooxygenase-2 in human colon cancer cells. Cancer Lett 210: 1-6.

Keller ET, Fu Z, Yeung K, Brennan M (2004) Raf kinase inhibitor protein: a prostate cancer metastasis suppressor gene. Cancer Lett 207: 131-137.

Jiang J, Slivova V, Valachovicova T, Harvey K, Sliva D (2004) Ganoderma lucidum inhibits proliferation and induces apoptosis in human prostate cancer cells PC-3. Int J Oncol 24: 1093-1099.

Je JH, Lee JY, Jung KJ, Sung B, Go EK, et al. (2004) NF-kappaB activation mechanism of 4-hydroxyhexenal via NIK/IKK and p38 MAPK pathway. FEBS Lett 566: 183-189.

Ismail HA, Lessard L, Mes-Masson AM, Saad F (2004) Expression of NF-kappaB in prostate cancer lymph node metastases. Prostate 58: 308-313.

Ikezoe T, Yang Y, Saito T, Koeffler HP, Taguchi H (2004) Proteasome inhibitor PS-341 down-regulates prostate-specific antigen (PSA) and induces growth arrest and apoptosis of androgen-dependent human prostate cancer LNCaP cells. Cancer Sci 95: 271-275.

Gunawardena K, Campbell LD, Meikle AW (2004) Combination therapy with vitamins C plus E inhibits survivin and human prostate cancer cell growth. Prostate 59: 319-327.

Dijsselbloem N, Vanden Berghe W, De Naeyer A, Haegeman G (2004) Soy isoflavone phyto-pharmaceuticals in interleukin-6 affections. Multi-purpose nutraceuticals at the crossroad of hormone replacement, anti-cancer and anti-inflammatory therapy. Biochem Pharmacol 68: 1171-1185.

Deeb D, Jiang H, Gao X, Hafner MS, Wong H, et al. (2004) Curcumin sensitizes prostate cancer cells to tumor necrosis factor-related apoptosis-inducing ligand/Apo2L by inhibiting nuclear factor-kappaB through suppression of IkappaBalpha phosphorylation. Mol Cancer Ther 3: 803-812.

Cinar B, Yeung F, Konaka H, Mayo MW, Freeman MR, et al. (2004) Identification of a negative regulatory cis-element in the enhancer core region of the prostate-specific antigen promoter: implications for intersection of androgen receptor and nuclear factor-kappaB signalling in prostate cancer cells. Biochem J 379: 421-431.

Chun KS, Surh YJ (2004) Signal transduction pathways regulating cyclooxygenase-2 expression: potential molecular targets for chemoprevention. Biochem Pharmacol 68: 1089-1100.

Califice S, Waltregny D, Castronovo V, van den Brule F (2004) [Prostate carcinoma cell lines and apoptosis: a review]. Rev Med Liege 59: 704-710.

Amorino GP, Parsons SJ (2004) Neuroendocrine cells in prostate cancer. Crit Rev Eukaryot Gene Expr 14: 287-300.

Aggarwal BB, Bhardwaj A, Aggarwal RS, Seeram NP, Shishodia S, et al. (2004) Role of resveratrol in prevention and therapy of cancer: preclinical and clinical studies. Anticancer Res 24: 2783-2840.

Zerbini LF, Wang Y, Cho JY, Libermann TA (2003) Constitutive activation of nuclear factor kappaB p50/p65 and Fra-1 and JunD is essential for deregulated interleukin 6 expression in prostate cancer. Cancer Res 63: 2206-2215.

Sliva D, Sedlak M, Slivova V, Valachovicova T, Lloyd FP, Jr., et al. (2003) Biologic activity of spores and dried powder from Ganoderma lucidum for the inhibition of highly invasive human breast and prostate cancer cells. J Altern Complement Med 9: 491-497.

Sliva D (2003) Ganoderma lucidum (Reishi) in cancer treatment. Integr Cancer Ther 2: 358-364.

Sakamoto KM, Kim KB, Verma R, Ransick A, Stein B, et al. (2003) Development of Protacs to target cancer-promoting proteins for ubiquitination and degradation. Mol Cell Proteomics 2: 1350-1358.

Park JI, Lee MG, Cho K, Park BJ, Chae KS, et al. (2003) Transforming growth factor-beta1 activates interleukin-6 expression in prostate cancer cells through the synergistic collaboration of the Smad2, p38-NF-kappaB, JNK, and Ras signaling pathways. Oncogene 22: 4314-4332.

Mack PC, Davies AM, Lara PN, Gumerlock PH, Gandara DR (2003) Integration of the proteasome inhibitor PS-341 (Velcade) into the therapeutic approach to lung cancer. Lung Cancer 41 Suppl 1: S89-96.

Lloyd FP, Jr., Slivova V, Valachovicova T, Sliva D (2003) Aspirin inhibits highly invasive prostate cancer cells. Int J Oncol 23: 1277-1283.

Liao X, Zhang L, Thrasher JB, Du J, Li B (2003) Glycogen synthase kinase-3beta suppression eliminates tumor necrosis factor-related apoptosis-inducing ligand resistance in prostate cancer. Mol Cancer Ther 2: 1215-1222.

Li Y, Li X, Sarkar FH (2003) Gene expression profiles of I3C- and DIM-treated PC3 human prostate cancer cells determined by cDNA microarray analysis. J Nutr 133: 1011-1019.

Lafuente MJ, Martin P, Garcia-Cao I, Diaz-Meco MT, Serrano M, et al. (2003) Regulation of mature T lymphocyte proliferation and differentiation by Par-4. EMBO J 22: 4689-4698.

Kandouz M, Nie D, Pidgeon GP, Krishnamoorthy S, Maddipati KR, et al. (2003) Platelet-type 12-lipoxygenase activates NF-kappaB in prostate cancer cells. Prostaglandins Other Lipid Mediat 71: 189-204.

Ikezoe T, Yang Y, Heber D, Taguchi H, Koeffler HP (2003) PC-SPES: a potent inhibitor of nuclear factor-kappa B rescues mice from lipopolysaccharide-induced septic shock. Mol Pharmacol 64: 1521-1529.

Hodge JC, Bub J, Kaul S, Kajdacsy-Balla A, Lindholm PF (2003) Requirement of RhoA activity for increased nuclear factor kappaB activity and PC-3 human prostate cancer cell invasion. Cancer Res 63: 1359-1364.

Hastak K, Gupta S, Ahmad N, Agarwal MK, Agarwal ML, et al. (2003) Role of p53 and NF-kappaB in epigallocatechin-3-gallate-induced apoptosis of LNCaP cells. Oncogene 22: 4851-4859.

Garcia-Cao I, Lafuente MJ, Criado LM, Diaz-Meco MT, Serrano M, et al. (2003) Genetic inactivation of Par4 results in hyperactivation of NF-kappaB and impairment of JNK and p38. EMBO Rep 4: 307-312.

Flynn V, Jr., Ramanitharan A, Moparty K, Davis R, Sikka S, et al. (2003) Adenovirus-mediated inhibition of NF-kappaB confers chemo-sensitization and apoptosis in prostate cancer cells. Int J Oncol 23: 317-323.

El-Guendy N, Zhao Y, Gurumurthy S, Burikhanov R, Rangnekar VM (2003) Identification of a unique core domain of par-4 sufficient for selective apoptosis induction in cancer cells. Mol Cell Biol 23: 5516-5525.

El-Guendy N, Rangnekar VM (2003) Apoptosis by Par-4 in cancer and neurodegenerative diseases. Exp Cell Res 283: 51-66.

Dhanalakshmi S, Agarwal R, Agarwal C (2003) Inhibition of NF-kappaB pathway in grape seed extract-induced apoptotic death of human prostate carcinoma DU145 cells. Int J Oncol 23: 721-727.

Catz SD, Johnson JL (2003) BCL-2 in prostate cancer: a minireview. Apoptosis 8: 29-37.

Altuwaijri S, Lin HK, Chuang KH, Lin WJ, Yeh S, et al. (2003) Interruption of nuclear factor kappaB signaling by the androgen receptor facilitates 12-O-tetradecanoylphorbolacetate-induced apoptosis in androgen-sensitive prostate cancer LNCaP cells. Cancer Res 63: 7106-7112.

Agarwal C, Dhanalakshmi S, Singh RP, Agarwal R (2003) Inositol hexaphosphate inhibits constitutive activation of NF- kappa B in androgen-independent human prostate carcinoma DU145 cells. Anticancer Res 23: 3855-3861.

Zhang H, Fu W (2002) NDPP1 is a novel CARD domain containing protein which can inhibit apoptosis and suppress NF-kappaB activation. Int J Oncol 20: 1035-1040.

Tozawa K, Okamoto T, Hayashi Y, Sasaki S, Kawai N, et al. (2002) N-acetyl-L-cysteine enhances chemotherapeutic effect on prostate cancer cells. Urol Res 30: 53-58.

Sliva D, Labarrere C, Slivova V, Sedlak M, Lloyd FP, Jr., et al. (2002) Ganoderma lucidum suppresses motility of highly invasive breast and prostate cancer cells. Biochem Biophys Res Commun 298: 603-612.

Shen W, Waldschmidt M, Zhao X, Ratliff T, Krieg AM (2002) Antitumor mechanisms of oligodeoxynucleotides with CpG and polyG motifs in murine prostate cancer cells: decrease of NF-kappaB and AP-1 binding activities and induction of apoptosis. Antisense Nucleic Acid Drug Dev 12: 155-164.

Segev DL, Hoshiya Y, Hoshiya M, Tran TT, Carey JL, et al. (2002) Mullerian-inhibiting substance regulates NF-kappa B signaling in the prostate in vitro and in vivo. Proc Natl Acad Sci U S A 99: 239-244.

Sarkar FH, Li Y (2002) Mechanisms of cancer chemoprevention by soy isoflavone genistein. Cancer Metastasis Rev 21: 265-280.

Pajonk F, Himmelsbach J, Riess K, Sommer A, McBride WH (2002) The human immunodeficiency virus (HIV)-1 protease inhibitor saquinavir inhibits proteasome function and causes apoptosis and radiosensitization in non-HIV-associated human cancer cells. Cancer Res 62: 5230-5235.

Nakamura K, Yasunaga Y, Segawa T, Ko D, Moul JW, et al. (2002) Curcumin down-regulates AR gene expression and activation in prostate cancer cell lines. Int J Oncol 21: 825-830.

Mayo MW, Madrid LV, Westerheide SD, Jones DR, Yuan XJ, et al. (2002) PTEN blocks tumor necrosis factor-induced NF-kappa B-dependent transcription by inhibiting the transactivation potential of the p65 subunit. J Biol Chem 277: 11116-11125.

Lundqvist A, Choudhury A, Nagata T, Andersson T, Quinn G, et al. (2002) Recombinant adenovirus vector activates and protects human monocyte-derived dendritic cells from apoptosis. Hum Gene Ther 13: 1541-1549.

Li Y, Sarkar FH (2002) Inhibition of nuclear factor kappaB activation in PC3 cells by genistein is mediated via Akt signaling pathway. Clin Cancer Res 8: 2369-2377.

Li Y, Sarkar FH (2002) Gene expression profiles of genistein-treated PC3 prostate cancer cells. J Nutr 132: 3623-3631.

Kimura K, Gelmann EP (2002) Propapoptotic effects of NF-kappaB in LNCaP prostate cancer cells lead to serine protease activation. Cell Death Differ 9: 972-980.

Kim BY, Gaynor RB, Song K, Dritschilo A, Jung M (2002) Constitutive activation of NF-kappaB in Ki-ras-transformed prostate epithelial cells. Oncogene 21: 4490-4497.

Huang Y, Fang Y, Dziadyk JM, Norris JS, Fan W (2002) The possible correlation between activation of NF-kappaB/IkappaB pathway and the susceptibility of tumor cells to paclitaxel-induced apoptosis. Oncol Res 13: 113-122.

Hour TC, Chen J, Huang CY, Guan JY, Lu SH, et al. (2002) Curcumin enhances cytotoxicity of chemotherapeutic agents in prostate cancer cells by inducing p21(WAF1/CIP1) and C/EBPbeta expressions and suppressing NF-kappaB activation. Prostate 51: 211-218.

Gupta S, Afaq F, Mukhtar H (2002) Involvement of nuclear factor-kappa B, Bax and Bcl-2 in induction of cell cycle arrest and apoptosis by apigenin in human prostate carcinoma cells. Oncogene 21: 3727-3738.

Gunawardena K, Murray DK, Swope RE, Meikle AW (2002) Inhibition of nuclear factor kappaB induces apoptosis following treatment with tumor necrosis factor alpha and an antioxidant in human prostate cancer cells. Cancer Detect Prev 26: 229-237.

Gunawardena K, Murray DK, Meikle AW (2002) Testosterone is a potential augmentor of antioxidant-induced apoptosis in human prostate cancer cells. Cancer Detect Prev 26: 105-113.

Gilman CP, Mattson MP (2002) Do apoptotic mechanisms regulate synaptic plasticity and growth-cone motility? Neuromolecular Med 2: 197-214.

Gasparian AV, Yao YJ, Kowalczyk D, Lyakh LA, Karseladze A, et al. (2002) The role of IKK in constitutive activation of NF-kappaB transcription factor in prostate carcinoma cells. J Cell Sci 115: 141-151.

El-Rayes BF, Grignon R, Aslam N, Aranha O, Sarkar FH (2002) Ciprofloxacin inhibits cell growth and synergises the effect of etoposide in hormone resistant prostate cancer cells. Int J Oncol 21: 207-211.

Dhanalakshmi S, Singh RP, Agarwal C, Agarwal R (2002) Silibinin inhibits constitutive and TNFalpha-induced activation of NF-kappaB and sensitizes human prostate carcinoma DU145 cells to TNFalpha-induced apoptosis. Oncogene 21: 1759-1767.

Coffey RN, Watson RW, O'Neill AJ, Mc Eleny K, Fitzpatrick JM (2002) Androgen-mediated resistance to apoptosis. Prostate 53: 300-309.

Chendil D, Das A, Dey S, Mohiuddin M, Ahmed MM (2002) Par-4, a pro-apoptotic gene, inhibits radiation-induced NF kappa B activity and Bcl-2 expression leading to induction of radiosensitivity in human prostate cancer cells PC-3. Cancer Biol Ther 1: 152-160.

Catz SD, Babior BM, Johnson JL (2002) JFC1 is transcriptionally activated by nuclear factor-kappaB and up-regulated by tumour necrosis factor alpha in prostate carcinoma cells. Biochem J 367: 791-799.

Zhang J, Johnston G, Stebler B, Keller ET (2001) Hydrogen peroxide activates NFkappaB and the interleukin-6 promoter through NFkappaB-inducing kinase. Antioxid Redox Signal 3: 493-504.

Zhang J, Dai J, Qi Y, Lin DL, Smith P, et al. (2001) Osteoprotegerin inhibits prostate cancer-induced osteoclastogenesis and prevents prostate tumor growth in the bone. J Clin Invest 107: 1235-1244.

Sanlioglu S, Luleci G, Thomas KW (2001) Simultaneous inhibition of Rac1 and IKK pathways sensitizes lung cancer cells to TNFalpha-mediated apoptosis. Cancer Gene Ther 8: 897-905.

Nishimura K, Nonomura N, Satoh E, Harada Y, Nakayama M, et al. (2001) Potential mechanism for the effects of dexamethasone on growth of androgen-independent prostate cancer. J Natl Cancer Inst 93: 1739-1746.

Mukhopadhyay A, Bueso-Ramos C, Chatterjee D, Pantazis P, Aggarwal BB (2001) Curcumin downregulates cell survival mechanisms in human prostate cancer cell lines. Oncogene 20: 7597-7609.

Meyer-Siegler K (2001) COX-2 specific inhibitor, NS-398, increases macrophage migration inhibitory factor expression and induces neuroendocrine differentiation in C4-2b prostate cancer cells. Mol Med 7: 850-860.

Kasof GM, Lu JJ, Liu D, Speer B, Mongan KN, et al. (2001) Tumor necrosis factor-alpha induces the expression of DR6, a member of the TNF receptor family, through activation of NF-kappaB. Oncogene 20: 7965-7975.

Huang S, Pettaway CA, Uehara H, Bucana CD, Fidler IJ (2001) Blockade of NF-kappaB activity in human prostate cancer cells is associated with suppression of angiogenesis, invasion, and metastasis. Oncogene 20: 4188-4197.

Gustin JA, Maehama T, Dixon JE, Donner DB (2001) The PTEN tumor suppressor protein inhibits tumor necrosis factor-induced nuclear factor kappa B activity. J Biol Chem 276: 27740-27744.

Gurumurthy S, Vasudevan KM, Rangnekar VM (2001) Regulation of apoptosis in prostate cancer. Cancer Metastasis Rev 20: 225-243.

Chinni SR, Li Y, Upadhyay S, Koppolu PK, Sarkar FH (2001) Indole-3-carbinol (I3C) induced cell growth inhibition, G1 cell cycle arrest and apoptosis in prostate cancer cells. Oncogene 20: 2927-2936.

Chakraborty M, Qiu SG, Vasudevan KM, Rangnekar VM (2001) Par-4 drives trafficking and activation of Fas and Fasl to induce prostate cancer cell apoptosis and tumor regression. Cancer Res 61: 7255-7263.

Catz SD, Johnson JL (2001) Transcriptional regulation of bcl-2 by nuclear factor kappa B and its significance in prostate cancer. Oncogene 20: 7342-7351.

Shi Q, Le X, Wang B, Xiong Q, Abbruzzese JL, et al. (2000) Regulation of interleukin-8 expression by cellular pH in human pancreatic adenocarcinoma cells. J Interferon Cytokine Res 20: 1023-1028.

Muenchen HJ, Lin DL, Walsh MA, Keller ET, Pienta KJ (2000) Tumor necrosis factor-alpha-induced apoptosis in prostate cancer cells through inhibition of nuclear factor-kappaB by an IkappaBalpha "super-repressor". Clin Cancer Res 6: 1969-1977.

Lindholm PF, Bub J, Kaul S, Shidham VB, Kajdacsy-Balla A (2000) The role of constitutive NF-kappaB activity in PC-3 human prostate cancer cell invasive behavior. Clin Exp Metastasis 18: 471-479.

Camandola S, Mattson MP (2000) Pro-apoptotic action of PAR-4 involves inhibition of NF-kappaB activity and suppression of BCL-2 expression. J Neurosci Res 61: 134-139.

Yang JP, Hori M, Sanda T, Okamoto T (1999) Identification of a novel inhibitor of nuclear factor-kappaB, RelA-associated inhibitor. J Biol Chem 274: 15662-15670.

Sumitomo M, Tachibana M, Nakashima J, Murai M, Miyajima A, et al. (1999) An essential role for nuclear factor kappa B in preventing TNF-alpha-induced cell death in prostate cancer cells. J Urol 161: 674-679.

Ripple MO, Henry WF, Schwarze SR, Wilding G, Weindruch R (1999) Effect of antioxidants on androgen-induced AP-1 and NF-kappaB DNA-binding activity in prostate carcinoma cells. J Natl Cancer Inst 91: 1227-1232.

Palayoor ST, Youmell MY, Calderwood SK, Coleman CN, Price BD (1999) Constitutive activation of IkappaB kinase alpha and NF-kappaB in prostate cancer cells is inhibited by ibuprofen. Oncogene 18: 7389-7394.

Pajonk F, Pajonk K, McBride WH (1999) Inhibition of NF-kappaB, clonogenicity, and radiosensitivity of human cancer cells. J Natl Cancer Inst 91: 1956-1960.

Nalca A, Qiu SG, El-Guendy N, Krishnan S, Rangnekar VM (1999) Oncogenic Ras sensitizes cells to apoptosis by Par-4. J Biol Chem 274: 29976-29983.

Meighan-Mantha RL, Riegel AT, Suy S, Harris V, Wang FH, et al. (1999) Ionizing radiation stimulates octamer factor DNA binding activity in human carcinoma cells. Mol Cell Biochem 199: 209-215.

Mattson MP, Duan W, Chan SL, Camandola S (1999) Par-4: an emerging pivotal player in neuronal apoptosis and neurodegenerative disorders. J Mol Neurosci 13: 17-30.

Diaz-Meco MT, Lallena MJ, Monjas A, Frutos S, Moscat J (1999) Inactivation of the inhibitory kappaB protein kinase/nuclear factor kappaB pathway by Par-4 expression potentiates tumor necrosis factor alpha-induced apoptosis. J Biol Chem 274: 19606-19612.

Ni A, Chai KX, Chao L, Chao J (1998) Molecular cloning and expression of rat bradykinin B1 receptor. Biochim Biophys Acta 1442: 177-185.

Herrmann JL, Briones F, Jr., Brisbay S, Logothetis CJ, McDonnell TJ (1998) Prostate carcinoma cell death resulting from inhibition of proteasome activity is independent of functional Bcl-2 and p53. Oncogene 17: 2889-2899.

Chaudhary PM, Ferguson C, Nguyen V, Nguyen O, Massa HF, et al. (1998) Cloning and characterization of two Toll/Interleukin-1 receptor-like genes TIL3 and TIL4: evidence for a multi-gene receptor family in humans. Blood 91: 4020-4027.

Nelson SA, Robins DM (1997) Regulatory capacity of an androgen-specific enhancer of the mouse Slp gene in transgenic mice. Mol Cell Endocrinol 133: 89-97.

Keller ET, Chang C, Ershler WB (1996) Inhibition of NFkappaB activity through maintenance of IkappaBalpha levels contributes to dihydrotestosterone-mediated repression of the interleukin-6 promoter. J Biol Chem 271: 26267-26275.
