# Supplementary material for: Cancer Biomarker Discovery: The Entropic Hallmark
Source: PLoS One. 2010 Aug 18;5(8):e12262. doi: 10.1371/journal.pone.0012262 (PMC2923618; doi:10.1371/journal.pone.0012262)
Supplement: File S5 — A full list of gene references in this paper along with their descriptions from iHOP (http://www.ihop-net.org/UniPub/iHOP/). (0.23 MB DOC) [file pone.0012262.s005.doc]

## Glossary of Gene Names and Aliases discussed

(as provided by iHOP – Information hyperlinked over proteins)

**ADA**

**Name:** adenosine deaminase

**Aliases:** Adenosine aminohydrolase, Adenosine deaminase

<http://www.ihop-net.org/UniPub/iHOP/gismo/86240.html>

**AKT1**

**Name:** v-akt murine thymoma viral oncogene homolog 1

**Aliases:** AKT, C-AKT, MGC99656, PKB, PKB-ALPHA, PRKBA, Protein kinase B, RAC, RAC-ALPHA, RAC-alpha serine/threonine-protein kinase, RAC-PK-alpha

<http://www.ihop-net.org/UniPub/iHOP/gismo/86340.html>

**ALS2CR2**

**Name:** STE20-related kinase adaptor beta

**Aliases:** ALS2CR2, Amyotrophic lateral sclerosis 2 chromosomal region candidate gene 2 protein, CALS-21, ILP-interacting protein, ILPIP, ILPIPA, MGC102916, PAPK, PRO1038, Pseudokinase ALS2CR2, STE20-related kinase adapter protein beta, STRAD beta

<http://www.ihop-net.org/UniPub/iHOP/gismo/100576.html>

**AMACR**

**Name:** AMACR alpha-methylacyl-CoA racemase

**Aliases:** 2-methylacyl-CoA racemase, Alpha-methylacyl-CoA racemase, CBAS4, RACE

<http://www.ihop-net.org/UniPub/iHOP/gismo/96980.html>

**ANK1**

**Name:** ankyrin 1, erythrocytic

**Aliases:** ANK, Ankyrin-1, Ankyrin-R, Erythrocyte ankyrin, SPH1, SPH2

<http://www.ihop-net.org/UniPub/iHOP/gismo/86415.html>

**ANKRD7**

**Name:** ankyrin repeat domain 7

**Aliases:** Ankyrin repeat domain-containing protein 7, Testis-specific protein TSA806, TSA806

<http://www.ihop-net.org/UniPub/iHOP/gs/101050.html>

**AQP1**

**Name:** aquaporin 1 (Colton blood group)

**Aliases:** AQP-1, AQP-CHIP, Aquaporin-1, Aquaporin-CHIP, CHIP28, CO, MGC26324, Urine water channel, Water channel protein for red blood cells and kidney proximal tubule

<http://www.ihop-net.org/UniPub/iHOP/gismo/86487.html>

**AQP3**

**Name:** aquaporin 3 (Gill blood group)

**Aliases:** AQP-3, Aquaporin-3, GIL

<http://www.ihop-net.org/UniPub/iHOP/gismo/86489.html>

**AQP5**

**Name:** aquaporin 5

**Aliases:** AQP-5, Aquaporin-5

<http://www.ihop-net.org/UniPub/iHOP/gismo/86491.html>

**AQP6**

**Name:** aquaporin 6, kidney specific

**Aliases:** AQP2L, AQP-6, Aquaporin-2-like, Aquaporin-6, hKID, KID, Kidney-specific aquaporin

<http://www.ihop-net.org/UniPub/iHOP/gismo/86492.html>

**AQP8**

**Name:** aquaporin 8

**Aliases:** AQP-8, Aquaporin-8

<http://www.ihop-net.org/UniPub/iHOP/gismo/86472.html>

**AQP9**

**Name:** aquaporin 9

**Aliases:** AQP-9, Aquaporin-9, HsT17287, Small solute channel 1, SSC1

<http://www.ihop-net.org/UniPub/iHOP/gismo/86494.html>

**AR**

**Name:** androgen receptor

**Aliases:** AIS, Androgen receptor, DHTR, Dihydrotestosterone receptor, HUMARA, HYSP1, KD, NR3C4, Nuclear receptor subfamily 3 group C member 4, SBMA, SMAX1, TFM

<http://www.ihop-net.org/UniPub/iHOP/gismo/86495.html>

**ARPC1B**

**Name:** actin related protein 2/3 complex, subunit 1B, 41kDa

**Aliases:** Actin-related protein 2/3 complex subunit 1B, ARC41, Arp2/3 complex 41 kDa subunit, p40-ARC, p41-ARC

<http://www.ihop-net.org/UniPub/iHOP/gismo/95232.html>

**ARPC2**

**Name:** actin related protein 2/3 complex, subunit 2, 34kDa

**Aliases:** Actin-related protein 2/3 complex subunit 2, ARC34, Arp2/3 complex 34 kDa subunit, p34-Arc, p34-ARC, PNAS-139, PRO2446

<http://www.ihop-net.org/UniPub/iHOP/gismo/95245.html>

**ASAP**

***See MAP9***

**ATP6AP2**

**Name:** ATPase, H+ transporting, lysosomal accessory protein 2

**Aliases:** APT6M8-9, ATP6IP2, ATP6M8-9, ATPase H(+)-transporting lysosomal accessory protein 2, ATPase H(+)-transporting lysosomal-interacting protein 2, CAPER, ELDF10, Embryonic liver differentiation factor 10, ER-localized type I transmembrane adaptor, HT028, M8-9, MGC99577, MRXE, MSTP009, N14F, PSEC0072, Renin/prorenin receptor, Renin receptor, Vacuolar ATP synthase membrane sector-associated protein M8-9, V-ATPase M8.9 subunit, XMRE

<http://www.ihop-net.org/UniPub/iHOP/gismo/95291.html>

**AURKA**

**Name:** aurora kinase A

**Aliases:** AIK, ARK1, AurA, AURA, Aurora/IPL1-related kinase 1, AURORA2, Aurora-A, Aurora kinase A, Aurora-related kinase 1, Breast tumor-amplified kinase, BTAK, hARK1, MGC34538, Serine/threonine kinase 15, Serine/threonine-protein kinase 6, STK15, STK6, STK7

<http://www.ihop-net.org/UniPub/iHOP/gismo/92453.html>

**AURKB**

**Name:** aurora kinase B

**Aliases:** Aik2, AIK2, AIM1, AIM-1, ARK2, AurB, Aurora/IPL1-related kinase 2, Aurora-and Ipl1-like midbody-associated protein 1, Aurora-B, Aurora-related kinase 2, IPL1, Serine/threonine-protein kinase 12, STK-1, STK12, STK5

<http://www.ihop-net.org/UniPub/iHOP/gismo/94471.html>

**BCAT1**

**Name:** branched chain aminotransferase 1, cytosolic

**Aliases:** BCAT(c), BCT1, Branched-chain-amino-acid aminotransferase, cytosolic, DKFZp686E12175, ECA39, MECA39, PNAS-121, PP18, Protein ECA39

<http://www.ihop-net.org/UniPub/iHOP/gismo/86699.html>

**BCCIP**

**Name:** BRCA2 and CDKN1A interacting protein

**Aliases:** BCCIPalpha, BRCA2 and CDKN1A-interacting protein, P21-and CDK-associated protein 1, Protein TOK-1, TOK1, TOK-1

<http://www.ihop-net.org/UniPub/iHOP/gismo/101073.html>

**BRCA1**

**Name:** breast cancer 1, early onset

**Aliases:** BRCAI, BRCC1, Breast cancer type 1 susceptibility protein, BROVCA1, IRIS, PSCP, RING finger protein 53, RNF53

<http://www.ihop-net.org/UniPub/iHOP/gismo/86781.html>

**BRCA2**

**Name:** breast cancer 2, early onset

**Aliases:** BRCC2, Breast cancer type 2 susceptibility protein, BROVCA2, FACD, FAD, FAD1, FANCB, FANCD, FANCD1, Fanconi anemia group D1 protein

<http://www.ihop-net.org/UniPub/iHOP/gismo/86784.html>

**BST2**

**Name:** bone marrow stromal cell antigen 2

**Aliases:** Bone marrow stromal antigen 2, BST-2, CD317, HM1.24 antigen, Tetherin

<http://www.ihop-net.org/UniPub/iHOP/gismo/86791.html>

**BTG2**

**Name:** BTG family, member 2

**Aliases:** BTG family member 2, MGC126063, MGC126064, NGF-inducible anti-proliferative protein PC3, PC3, Protein BTG2, TIS21

<http://www.ihop-net.org/UniPub/iHOP/gismo/93404.html>

**BUB1**

**Name:** budding uninhibited by benzimidazoles 1 homolog (yeast)

**Aliases:** BUB1A, BUB1L, hBUB1, Mitotic checkpoint serine/threonine-protein kinase BUB1

<http://www.ihop-net.org/UniPub/iHOP/gismo/86806.html>

**c-FOS**

***See FOS***

**c-MYC**

***See MYC***

**C14orf123**

***See CHMP4A***

**C18orf45**

**Name:** chromosome 18 open reading frame 45

**Aliases:** FLJ44259, MGC11386, MGC138577, Transmembrane protein C18orf45

<http://www.ihop-net.org/UniPub/iHOP/mgi/104463.html>

**CCL2**

**Name:** chemokine (C-C motif) ligand 2

**Aliases:** C-C motif chemokine 2, GDCF-2, HC11, HSMCR30, MCAF, MCP1, MCP-1, MGC9434, Monocyte chemoattractant protein 1, Monocyte chemotactic and activating factor, Monocyte chemotactic protein 1, Monocyte secretory protein JE, SCYA2, Small-inducible cytokine A2, SMC-CF

<http://www.ihop-net.org/UniPub/iHOP/gismo/92036.html>

**CCL3**

**Name:** chemokine (C-C motif) ligand 3

**Aliases:** C-C motif chemokine 3, G0/G1 switch regulatory protein 19-1, G0S19-1, G0S19-1 protein, LD78ALPHA, Macrophage inflammatory protein 1-alpha, MIP1A, MIP-1-alpha, PAT 464.1, SCYA3, SIS-beta, Small-inducible cytokine A3, Tonsillar lymphocyte LD78 alpha protein

<http://www.ihop-net.org/UniPub/iHOP/gismo/92037.html>

**CCNG2**

**Name:** cyclin G2

**Aliases:** Cyclin-G2

<http://www.ihop-net.org/UniPub/iHOP/gismo/86984.html>

**CCT4**

**Name:** chaperonin containing TCP1, subunit 4 (delta)

**Aliases:** Cctd, CCTD, CCT-delta, CCT-DELTA, MGC126164, MGC126165, SRB, Stimulator of TAR RNA-binding, T-complex protein 1 subunit delta, TCP-1-delta

<http://www.ihop-net.org/UniPub/iHOP/gismo/95660.html>

**CD40**

**Name:** CD40 molecule, TNF receptor superfamily member 5

**Aliases:** B-cell surface antigen CD40, Bp50, CD40L receptor, CDw40, CDW40, MGC9013, p50, TNFRSF5, Tumor necrosis factor receptor superfamily member 5 precursor

<http://www.ihop-net.org/UniPub/iHOP/gs/87039.html>

**CD59**

**Name:** CD59 molecule, complement regulatory protein

**Aliases:** 16.3A5, 1F5, 1F5 antigen, 20 kDa homologous restriction factor, CD59 glycoprotein precursor, EJ16, EJ30, EL32, FLJ38134, FLJ92039, G344, HRF20, HRF-20, MACIF, MAC-inhibitory protein, MAC-IP, MEM43, MEM43 antigen, Membrane attack complex inhibition factor, Membrane inhibitor of reactive lysis, MGC2354, MIC11, MIN1, MIN2, MIN3, MIRL, MSK21, p18-20, Protectin, PROTECTIN

<http://www.ihop-net.org/UniPub/iHOP/gismo/87047.html>

**CD230**

***See PRNP***

**CD302**

**Name:** CD302 molecule

**Aliases:** BIMLEC, CD302 antigen, CLEC13A, C-type lectin BIMLEC, C-type lectin domain family 13 member A, DCL1, DCL-1, KIAA0022, Type I transmembrane C-type lectin receptor DCL-1

<http://www.ihop-net.org/UniPub/iHOP/gismo/95091.html>

**CDC7**

**Name:** cell division cycle 7 homolog (S. cerevisiae)

**Aliases:** CDC7L1, CDC7-related kinase, Cell division cycle 7-related protein kinase, HsCdc7, HsCDC7, Hsk1, huCdc7, huCDC7, MGC117361, MGC126237, MGC126238

<http://www.ihop-net.org/UniPub/iHOP/gismo/93737.html>

**CDCA5**

**Name:** cell division cycle associated 5

**Aliases:** Cell division cycle-associated protein 5, MGC16386, p35, Sororin, SORORIN

<http://www.ihop-net.org/UniPub/iHOP/gismo/105273.html>

**CDK4**

**Name:** CDK4 cyclin-dependent kinase 4

**Aliases:** Cell division protein kinase 4, CMM3, Cyclin-dependent kinase 4, MGC14458, PSK-J3

<http://www.ihop-net.org/UniPub/iHOP/gismo/87097.html>

**CDK7**

Name: CDK7 cyclin-dependent kinase 7

Aliases: 39 kDa protein kinase, CAK, CAK1, CDK-activating kinase, CDKN7, Cell division protein kinase 7, MO15, p39MO15, P39 Mo15, STK1, TFIIH basal transcription factor complex kinase subunit

<http://www.ihop-net.org/UniPub/iHOP/gismo/87100.html>

**CDKN1A**

**Name:** cyclin-dependent kinase inhibitor 1A (p21, Cip1)

**Aliases:** CAP20, CDK-interacting protein 1, CDKN1, CIP1, Cyclin-dependent kinase inhibitor 1, MDA6, MDA-6, Melanoma differentiation-associated protein 6, p21, P21, p21CIP1, p21Cip1/Waf1, PIC1, SDI1, WAF1

<http://www.ihop-net.org/UniPub/iHOP/gismo/87103.html>

**CDKN2A**

**Name:** cyclin-dependent kinase inhibitor 2A (melanoma, p16, inhibits CDK4)

**Aliases:** ARF, CDK4I, CDKN2, CMM2, Cyclin-dependent kinase 4 inhibitor A, Cyclin-dependent kinase inhibitor 2A, isoform 4, Cyclin-dependent kinase inhibitor 2A, isoforms 1/2/3, INK4, INK4a, MLM, MTS1, MTS-1, Multiple tumor suppressor 1, p14, p14ARF, p16, p16INK4, p16-INK4, p16INK4a, p16INK4A, p16-INK4a, p19, p19ARF, TP16

<http://www.ihop-net.org/UniPub/iHOP/gismo/87106.html>

**CDKN2C**

**Name:** cyclin-dependent kinase inhibitor 2C (p18, inhibits CDK4)

**Aliases:** CDKN6, Cyclin-dependent kinase 4 inhibitor C, Cyclin-dependent kinase 6 inhibitor, INK4C, p18, p18-INK4c, p18-INK4C, p18-INK6

<http://www.ihop-net.org/UniPub/iHOP/gismo/87108.html>

**CDKN2D**

**Name:** cyclin-dependent kinase inhibitor 2D (p19, inhibits CDK4)

**Aliases:** Cyclin-dependent kinase 4 inhibitor D, INK4D, p19, p19-INK4d, p19-INK4D

<http://www.ihop-net.org/UniPub/iHOP/gismo/87109.html>

**CDSN**

**Name:** corneodesmosin

**Aliases:** Corneodesmosin, D6S586E, HTSS, S protein

<http://www.ihop-net.org/UniPub/iHOP/gismo/87115.html>

**CENPF**

**Name:** centromere protein F, 350/400ka (mitosin)

**Aliases:** AH antigen, CENF, Centromere protein F, hcp-1, Kinetochore protein CENP-F, Mitosin, PRO1779

<http://www.ihop-net.org/UniPub/iHOP/gismo/87135.html>

**CGH**

***See HTC2***

**CHEK1**

**Name:** CHK1 checkpoint homolog (S. pombe)

**Aliases:** CHK1, Serine/threonine-protein kinase Chk1

<http://www.ihop-net.org/UniPub/iHOP/gismo/87179.html>

**CHI3L1**

**Name:** chitinase 3-like 1 (cartilage glycoprotein-39)

**Aliases:** 39 kDa synovial protein, ASRT7, Cartilage glycoprotein 39, CGP-39, Chitinase-3-like protein 1, DKFZp686N19119, FLJ38139, GP39, GP-39, hCGP-39, HC-gp39, HCGP-3P, YKL40, YKL-40, YYL-40

<http://www.ihop-net.org/UniPub/iHOP/gismo/87183.html>

**CHMP4A**

**Name:** chromatin modifying protein 4A

**Aliases:** C14orf123, CDA04, Charged multivesicular body protein 4a, CHMP4, CHMP4a, CHMP4B, Chromatin-modifying protein 4a, FLJ61658, hSnf-1, HSPC134, hVps32-1, MGC142093, MGC142095, Shax2, SHAX2, SNF7, SNF7-1, SNF7 homolog associated with Alix-2, Vacuolar protein-sorting-associated protein 32-1, Vps32-1, VPS32A

<http://www.ihop-net.org/UniPub/iHOP/gismo/98738.html>

**CITED1**

**Name:** Cbp/p300-interacting transactivator, with Glu/Asp-rich carboxy-terminal domain, 1

**Aliases:** Cbp/p300-interacting transactivator 1, Melanocyte-specific protein 1, MSG1

<http://www.ihop-net.org/UniPub/iHOP/gismo/90255.html>

**CKLFSF7**

***See CMTM7***

**CLDN1**

**Name:** claudin 1

**Aliases:** Claudin-1, CLD1, ILVASC, SEMP1, Senescence-associated epithelial membrane protein, UNQ481/PRO944

<http://www.ihop-net.org/UniPub/iHOP/gismo/94358.html>

**CMTM7**

**Name:** CKLF-like MARVEL transmembrane domain containing 7

**Aliases:** Chemokine-like factor superfamily member 7, CKLF-like MARVEL transmembrane domain-containing protein 7, CKLFSF7, FLJ30992

<http://www.ihop-net.org/UniPub/iHOP/mgi/105245.html>

**COL16A1**

**Name:** collagen, type XVI, alpha 1

**Aliases:** 447AA, Collagen alpha-1(XVI) chain, FP1572

<http://www.ihop-net.org/UniPub/iHOP/gismo/87346.html>

**COL17A1**

**Name:** collagen, type XVII, alpha 1

**Aliases:** 180 kDa bullous pemphigoid antigen 2, BA16H23.2, BP180, BPAG2, Bullous pemphigoid antigen 2, Collagen alpha-1(XVII) chain, FLJ60881, KIAA0204, LAD-1

<http://www.ihop-net.org/UniPub/iHOP/gismo/87347.html>

**COMMD6**

**Name:** COMM domain containing 6

**Aliases:** Acrg, COMM domain-containing protein 6, MSTP076

<http://www.ihop-net.org/UniPub/iHOP/mgi/108238.html>

**CRABP2**

**Name:** cellular retinoic acid binding protein 2

**Aliases:** Cellular retinoic acid-binding protein 2, Cellular retinoic acid-binding protein II, CRABP-II, RBP6

<http://www.ihop-net.org/UniPub/iHOP/gismo/87416.html>

**CSRP1**

**Name:** cysteine and glycine-rich protein 1

**Aliases:** CRP, CRP1, CSRP, CYRP, Cysteine and glycine-rich protein 1, Cysteine-rich protein 1, D1S181E, DKFZp686M148

<http://www.ihop-net.org/UniPub/iHOP/gismo/87490.html>

**CTBP2**

**Name:** C-terminal binding protein 2

**Aliases:** CtBP2, C-terminal-binding protein 2, ribeye

<http://www.ihop-net.org/UniPub/iHOP/gismo/87513.html>

**CTGF**

**Name:** connective tissue growth factor

**Aliases:** CCN2, Connective tissue growth factor, HCS24, Hypertrophic chondrocyte-specific protein 24, IGFBP8, MGC102839, NOV2

<http://www.ihop-net.org/UniPub/iHOP/gismo/87515.html>

**CXCL1**

**Name:** chemokine (C-X-C motif) ligand 1 (melanoma growth stimulating activity, alpha)

**Aliases:** C-X-C motif chemokine 1, FSP, GRO, GRO1, GROa, GROA, GRO-alpha(1-73), Growth-regulated alpha protein, Melanoma growth stimulatory activity, MGSA, MGSA-a, NAP-3, Neutrophil-activating protein 3, SCYB1

<http://www.ihop-net.org/UniPub/iHOP/gismo/88842.html>

**CXCL10**

**Name:** chemokine (C-X-C motif) ligand 10

**Aliases:** 10 kDa interferon-gamma-induced protein, C7, crg-2, C-X-C motif chemokine 10, Gamma-IP10, gIP-10, IFI10, INP10, IP-10, mob-1, SCYB10, Small-inducible cytokine B10

<http://www.ihop-net.org/UniPub/iHOP/gismo/89508.html>

**Cyclin G2**

***See CCNG2***

**CYFIP2**

**Name:** cytoplasmic FMR1 interacting protein 2

**Aliases:** Cytoplasmic FMR1-interacting protein 2, KIAA1168, p53-inducible protein 121, PIR121

<http://www.ihop-net.org/UniPub/iHOP/gismo/97828.html>

**DCR**

**Name:** Down syndrome chromosome region

**Aliases:** DSCR

<http://www.ihop-net.org/UniPub/iHOP/gismo/87649.html>

**DES**

**Name:** desmin

**Aliases:** CMD1I, CSM1, CSM2, Desmin, FLJ12025, FLJ39719, FLJ41013, FLJ41793

<http://www.ihop-net.org/UniPub/iHOP/gismo/87683.html>

**DKK1**

**Name:** dickkopf homolog 1 (Xenopus laevis)

**Aliases:** Dickkopf-1, Dickkopf-related protein 1, Dkk-1, DKK-1, hDkk-1, SK, UNQ492/PRO1008

<http://www.ihop-net.org/UniPub/iHOP/gismo/96438.html>

**DMN**

**Name:** synemin, intermediate filament protein

**Aliases:** Desmuslin, DMN, KIAA0353, SYN, Synemin

<http://www.ihop-net.org/UniPub/iHOP/gismo/96766.html>

**DNAJB1**

**Name:** DnaJ (Hsp40) homolog, subfamily B, member 1

**Aliases:** DNAJ1, DnaJ homolog subfamily B member 1, DnaJ protein homolog 1, Hdj1, HDJ1, HDJ-1, Heat shock 40 kDa protein 1, Heat shock protein 40, Hsp40, HSP40, HSPF1, Sis1

<http://www.ihop-net.org/UniPub/iHOP/gismo/89236.html>

**DNAJC6**

**Name:** DnaJ (Hsp40) homolog, subfamily C, member 6

**Aliases:** DJC6, DnaJ homolog subfamily C member 6, KIAA0473, MGC129914, MGC129915, MGC48436

<http://www.ihop-net.org/UniPub/iHOP/mgi/94996.html>

**DPP4**

**Name:** dipeptidyl-peptidase 4

**Aliases:** ADABP, ADCP2, Adenosine deaminase complexing protein 2, CD26, Dipeptidyl peptidase 4, Dipeptidyl peptidase IV, DPPIV, DPP IV, T-cell activation antigen CD26, TP103

<http://www.ihop-net.org/UniPub/iHOP/gismo/87784.html>

**DSC2**

**Name:** desmocollin 2

**Aliases:** ARVD11, CDHF2, Desmocollin-2, Desmocollin-3, Desmosomal glycoprotein II and III, DG2, DGII/III, DKFZp686I11137, DSC3

<http://www.ihop-net.org/UniPub/iHOP/gismo/87805.html>

**DSC54**

***See PRR16***

**DSCR**

***See DCR***

**DSG1**

**Name:** desmoglein 1

**Alias:** CDHF4, Desmoglein-1, Desmosomal glycoprotein 1, DG1, DGI, DSG, Pemphigus foliaceus antigen, PPKS1, SPPK1

<http://www.ihop-net.org/UniPub/iHOP/gismo/87809.html>

**DSP**

**Name:** desmoplakin

**Aliases:** 250/210 kDa paraneoplastic pemphigus antigen, Desmoplakin, DP, DPI, DPII, KPPS2, PPKS2

<http://www.ihop-net.org/UniPub/iHOP/gismo/87813.html>

**DST**

**Name:** dystonin

**Alias:** 230 kDa bullous pemphigoid antigen, BP240, BPA, BPAG1, Bullous pemphigoid antigen, Bullous pemphigoid antigen 1, isoform 7, Bullous pemphigoid antigen 1, isoforms 1/2/3/4/5/8, Bullous pemphigoid antigen 1, isoforms 6/9/10, CATX-15, D6S1101, DKFZp564B2416, DMH, DT, Dystonia musculorum protein, Dystonin, FLJ13425, FLJ21489, FLJ30627, FLJ32235, FLJ46791, Hemidesmosomal plaque protein, KIAA0465, KIAA0728, KIAA1470, MACF2, Trabeculin-beta

<http://www.ihop-net.org/UniPub/iHOP/gismo/86776.html>

**EFG**

***See GFM1***

**EGF**

**Name:** epidermal growth factor (beta-urogastrone)

**Aliases:** HOMG4, Pro-epidermal growth factor, URG

<http://www.ihop-net.org/UniPub/iHOP/gismo/87917.html>

**EMP1**

**Name:** epithelial membrane protein 1

**Aliases:** B4B, CL-20, EMP-1, Epithelial membrane protein 1, Protein B4B, TMP, Tumor-associated membrane protein

<http://www.ihop-net.org/UniPub/iHOP/gismo/87974.html>

**EPHB2**

**Name:** EPH receptor B2

**Aliases:** CAPB, DRT, Ephrin type-B receptor 2, EPHT3, EPTH3, ERK, Hek5, HEK5, MGC87492, PCBC, Receptor protein-tyrosine kinase HEK5, Renal carcinoma antigen NY-REN-47, Tyro5, TYRO5, Tyrosine-protein kinase receptor EPH-3, Tyrosine-protein kinase TYRO5

<http://www.ihop-net.org/UniPub/iHOP/gismo/88009.html>

**ERBB2**

**Name:** v-erb-b2 erythroblastic leukemia viral oncogene homolog 2, neuro/glioblastoma derived oncogene homolog (avian)

**Aliases:** CD340, C-erbB-2, HER2, HER-2, HER-2/neu, MLN 19, NEU, NEU proto-oncogene, NGL, p185erbB2, Receptor tyrosine-protein kinase erbB-2, TKR1, Tyrosine kinase-type cell surface receptor HER2

<http://www.ihop-net.org/UniPub/iHOP/gismo/88023.html>

**ERCC5**

**Name:** excision repair cross-complementing rodent repair deficiency, complementation group 5

**Aliases:**   COFS3, DNA excision repair protein ERCC-5, DNA repair protein complementing XP-G cells, ERCM2, UVDR, Xeroderma pigmentosum group G-complementing protein, XPG, XPGC

<http://www.ihop-net.org/UniPub/iHOP/gismo/88032.html>

**ESR1**

**Name:** estrogen receptor 1

**Aliases:** DKFZp686N23123, ER, Era, ER-alpha, ESR, ESRA, Estradiol receptor, Estrogen receptor, NR3A1, Nuclear receptor subfamily 3 group A member 1

<http://www.ihop-net.org/UniPub/iHOP/gismo/88056.html>

**EVPL**

**Name:** envoplakin

**Aliases:** 210 kDa cornified envelope precursor protein, 210 kDa paraneoplastic pemphigus antigen, Envoplakin, EVPK, p210

<http://www.ihop-net.org/UniPub/iHOP/gismo/88079.html>

**FHL2**

**Name:** four and a half LIM domains 2

**Aliases:** AAG11, DRAL, FHL-2, Four and a half LIM domains protein 2, LIM domain protein DRAL, Skeletal muscle LIM-protein 3, SLIM3, SLIM 3

<http://www.ihop-net.org/UniPub/iHOP/gismo/88220.html>

**FLT1**

**Name:** fms-related tyrosine kinase 1 (vascular endothelial growth factor/vascular permeability factor receptor)

**Aliases:** FLT, Flt-1, Fms-like tyrosine kinase 1, FRT, Tyrosine-protein kinase FRT, Tyrosine-protein kinase receptor FLT, Vascular endothelial growth factor receptor 1, Vascular permeability factor receptor, VEGFR1, VEGFR-1

<http://www.ihop-net.org/UniPub/iHOP/gismo/88264.html>

**FOS**

**Name:** v-fos FBJ murine osteosarcoma viral oncogene homolog

**Aliases:** AP-1, Cellular oncogene fos, c-fos, C-FOS, G0/G1 switch regulatory protein 7, G0S7, Proto-oncogene protein c-fos

<http://www.ihop-net.org/UniPub/iHOP/gismo/88293.html>

**FOXM1**

**Name:** forkhead box M1

**Aliases:** FKHL16, Forkhead box protein M1, Forkhead-related protein FKHL16, FOXM1B, Hepatocyte nuclear factor 3 forkhead homolog 11, HFH11, HFH-11, HNF-3, HNF-3/fork-head homolog 11, INS-1, M-phase phosphoprotein 2, MPHOSPH2, MPM-2 reactive phosphoprotein 2, MPP2, MPP-2, PIG29, TGT3, Transcription factor Trident, trident, TRIDENT, WIN, Winged-helix factor from INS-1 cells

<http://www.ihop-net.org/UniPub/iHOP/gismo/88249.html>

**FPR2**

**Name:** formyl peptide receptor 2

**Aliases:** ALXR, FMLP-related receptor I, FMLP-R-I, FMLP-R-II, FMLPX, Formyl peptide receptor-like 1, FPR2A, FPRH1, FPRH2, FPRL1, HM63, Lipoxin A4 receptor, LXA4R, LXA4 receptor, N-formyl peptide receptor 2, RFP

<http://www.ihop-net.org/UniPub/iHOP/gismo/88298.html>

**FPRL1**

***See FPR2***

**FTH1**

**Name:** ferritin, heavy polypeptide 1

**Aliases:** Cell proliferation-inducing gene 15 protein, Ferritin heavy chain, Ferritin H subunit, FHC, FTH, FTHL6, MGC104426, OK/SW-cl.84, PIG15, PLIF

<http://www.ihop-net.org/UniPub/iHOP/gismo/88434.html>

**GAPDH**

**Name:** glyceraldehyde-3-phosphate dehydrogenase

**Aliases:** CDABP0047, G3PD, GAPD, Glyceraldehyde-3-phosphate dehydrogenase, MGC88685, OK/SW-cl.12

<http://www.ihop-net.org/UniPub/iHOP/gismo/88530.html>

**GCHFR**

**Name:** GTP cyclohydrolase I feedback regulator

**Aliases:** GFRP, GTP cyclohydrolase 1 feedback regulatory protein, GTP cyclohydrolase I feedback regulatory protein, HsT16933, MGC138467, MGC138469, p35, P35

<http://www.ihop-net.org/UniPub/iHOP/gismo/88576.html>

**GFM1**

**Name:** G elongation factor, mitochondrial 1

**Aliases:** COXPD1, EFG, EFG1, EFGM, EGF1, Elongation factor G1, Elongation factor G 1, mitochondrial, FLJ12662, FLJ13632, FLJ20773, GFM, hEFG1, mEF-G 1

<http://www.ihop-net.org/UniPub/iHOP/gs/104582.html>

**GJB5**

Name: gap junction protein, beta 5, 31.1kDa

Aliases: Connexin-31.1, Cx31.1, CX31.1, Gap junction beta-5 protein

<http://www.ihop-net.org/UniPub/iHOP/gismo/88637.html>

**GPR115**

**Name:** G protein-coupled receptor 115

**Aliases:** FLJ38076, G-protein coupled receptor PGR18, PGR18

<http://www.ihop-net.org/UniPub/iHOP/gismo/108929.html>

**GRIPAP1**

**Name:** GRIP1 associated protein 1

**Aliases:** DKFZp434P0630, GRASP1, GRASP-1, GRIP1-associated protein 1, KIAA1167, MGC126593, MGC126595, MPMGp800B12492Q3

<http://www.ihop-net.org/UniPub/iHOP/gismo/101123.html>

**GSTP1**

**Name:** glutathione S-transferase pi 1

**Aliases:** DFN7, FAEES3, Glutathione S-transferase P, GST3, GST class-pi, GSTP1-1, PI

<http://www.ihop-net.org/UniPub/iHOP/gismo/88871.html>

**GSTM1**

**Name:** glutathione S-transferase mu 1

**Aliases:** Glutathione S-transferase Mu 1, GST1, GST class-mu 1, GSTM1-1, GSTM1a-1a, GSTM1b-1b, GTH4, GTM1, H-B, HB subunit 4, MGC26563, MU, MU-1

<http://www.ihop-net.org/UniPub/iHOP/gismo/88865.html>

**HBEGF**

**Name:** heparin-binding EGF-like growth factor

**Aliases:** DTR, DTS, DTSF, HEGFL, Proheparin-binding EGF-like growth factor

<http://www.ihop-net.org/UniPub/iHOP/gismo/87819.html>

**HER2**

***See ERBB2***

**HLA-A**

**Name:** major histocompatibility complex, class I, A

**Aliases:** A-1, A-10, A-28, A-9, Aw-19, Aw-24, Aw-33, Aw-34, Aw-36, Aw-43, Aw-66, Aw-68, Aw-69, Aw-74, Aw-80, FLJ26655, HLAA, HLA class I histocompatibility antigen, A-11 alpha chain, HLA class I histocompatibility antigen, A-1 alpha chain, HLA class I histocompatibility antigen, A-23 alpha chain, HLA class I histocompatibility antigen, A-24 alpha chain, HLA class I histocompatibility antigen, A-25 alpha chain, HLA class I histocompatibility antigen, A-26 alpha chain, HLA class I histocompatibility antigen, A-29 alpha chain, HLA class I histocompatibility antigen, A-2 alpha chain, HLA class I histocompatibility antigen, A-30 alpha chain, HLA class I histocompatibility antigen, A-31 alpha chain, HLA class I histocompatibility antigen, A-32 alpha chain, HLA class I histocompatibility antigen, A-33 alpha chain, HLA class I histocompatibility antigen, A-34 alpha chain, HLA class I histocompatibility antigen, A-36 alpha chain, HLA class I histocompatibility antigen, A-3 alpha chain, HLA class I histocompatibility antigen, A-43 alpha chain, HLA class I histocompatibility antigen, A-66 alpha chain, HLA class I histocompatibility antigen, A-68 alpha chain, HLA class I histocompatibility antigen, A-69 alpha chain, HLA class I histocompatibility antigen, A-74 alpha chain, HLA class I histocompatibility antigen, A-80 alpha chain, MHC class I antigen A*1, MHC class I antigen A*11, MHC class I antigen A*2, MHC class I antigen A*23, MHC class I antigen A*24, MHC class I antigen A*25, MHC class I antigen A*26, MHC class I antigen A*29, MHC class I antigen A*3, MHC class I antigen A*30, MHC class I antigen A*31, MHC class I antigen A*32, MHC class I antigen A*33, MHC class I antigen A*34, MHC class I antigen A*36, MHC class I antigen A*43, MHC class I antigen A*66, MHC class I antigen A*68, MHC class I antigen A*69, MHC class I antigen A*74, MHC class I antigen A*80

<http://www.ihop-net.org/UniPub/iHOP/gismo/89016.html>

**HPN**

**Name:** hepsin

**Aliases:** Serine protease hepsin, TMPRSS1, Transmembrane protease, serine 1

<http://www.ihop-net.org/UniPub/iHOP/gismo/89153.html>

**HTC2**

**Name:** hypertrichosis 2 (generalized, congenital)

**Aliases:** CGH, HCG

<http://www.ihop-net.org/UniPub/iHOP/gs/89241.html>

**IFI6**

**Name:** interferon, alpha-inducible protein 6

**Aliases:** 6-16, FAM14C, G1P3, IFI616, Ifi-6-16, IFI-6-16, Interferon alpha-inducible protein 6, Interferon-induced protein 6-16

<http://www.ihop-net.org/UniPub/iHOP/gismo/88473.html>

**IFI16**

**Name:** interferon, gamma-inducible protein 16

**Aliases:** Gamma-interferon-inducible protein Ifi-16, IFI 16, IFNGIP1, Interferon-inducible myeloid differentiation transcriptional activator, MGC9466, PYHIN2

<http://www.ihop-net.org/UniPub/iHOP/gismo/89322.html>

**IL4R**

**Name:** interleukin 4 receptor

**Aliases:** 582J2.1, CD124, IL4RA, IL-4R-alpha, Interleukin-4 receptor alpha chain

<http://www.ihop-net.org/UniPub/iHOP/gismo/89449.html>

**INK4A**

***See CDKN2A***

**ITGA3**

**Name:** integrin, alpha 3 (antigen CD49C, alpha 3 subunit of VLA-3 receptor)

**Aliases:** CD49 antigen-like family member C, CD49c, CD49C, FLJ34631, FLJ34704, FRP-2, Galactoprotein B3, GAPB3, GAP-B3, Integrin alpha-3, MSK18, VCA-2, VL3A, VLA3a, VLA-3 alpha chain

<http://www.ihop-net.org/UniPub/iHOP/gismo/89554.html>

**ITGA7**

**Name:** integrin, alpha 7

**Aliases:** Integrin alpha-7, UNQ406/PRO768

<http://www.ihop-net.org/UniPub/iHOP/gismo/89557.html>

**JUNB**

**Name:** jun B proto-oncogene

**Aliases:** Transcription factor jun-B

<http://www.ihop-net.org/UniPub/iHOP/gismo/89601.html>

**JUP**

**Name:** junction plakoglobin

**Aliases:** ARVD12, Catenin gamma, CTNNG, Desmoplakin-3, Desmoplakin III, DP3, DPIII, Junction plakoglobin, PDGB, PKGB

<http://www.ihop-net.org/UniPub/iHOP/gismo/89603.html>

**KET**

***See TP63***

**KIAA0152**

**Name:** malectin

**Aliases:** KIAA0152, Malectin

<http://www.ihop-net.org/UniPub/iHOP/gismo/94936.html>

**KIAA1191**

**Name:** KIAA1191

**Aliases:** FLJ21022, p60MONOX, UPF0498 protein KIAA1191

<http://www.ihop-net.org/UniPub/iHOP/mgi/101344.html>

**KIAA1324**

**Name:** KIAA1324

**Aliases:** EIG121, Estrogen-induced gene 121 protein, maba1, MGC150624, UNQ2426/PRO4985, UPF0577 protein KIAA1324

<http://www.ihop-net.org/UniPub/iHOP/gismo/101514.html>

**KIF23**

**Name:** kinesin family member 23

**Aliases:** CHO1, Kinesin-like protein 5, Kinesin-like protein KIF23, KNSL5, Mitotic kinesin-like protein 1, MKLP1, MKLP-1

<http://www.ihop-net.org/UniPub/iHOP/gismo/94699.html>

**KLF4**

**Names:** Kruppel-like factor 4 (gut)

**Aliases:** Epithelial zinc finger protein EZF, EZF, GKLF, Gut-enriched krueppel-like factor, Krueppel-like factor 4

<http://www.ihop-net.org/UniPub/iHOP/gismo/94551.html>

**KLF6**

**Name:** Kruppel-like factor 6

**Aliases:** BCD1, B-cell-derived protein 1, CBA1, COPEB, Core promoter element-binding protein, CPBP, DKFZp686N0199, GBF, GC-rich sites-binding factor GBF, Krueppel-like factor 6, PAC1, Proto-oncogene BCD1, ST12, Transcription factor Zf9, Zf9, ZF9

<http://www.ihop-net.org/UniPub/iHOP/gismo/87354.html>

**KLK3**

**Name:** kallikrein-related peptidase 3

**Aliases:** APS, Gamma-seminoprotein, hK3, Kallikrein-3, KLK2A1, P-30 antigen, Prostate-specific antigen, PSA, Semenogelase, Seminin

<http://www.ihop-net.org/UniPub/iHOP/gismo/86483.html>

**KLK4**

**Name:** kallikrein-related peptidase 4

**Aliases:** ARM1, EMSP, EMSP1, Enamel matrix serine proteinase 1, Kallikrein-4, Kallikrein-like protein 1, KLK-L1, MGC116827, MGC116828, Prostase, PROSTASE, PRSS17, PSTS, Serine protease 17

<http://www.ihop-net.org/UniPub/iHOP/gismo/94809.html>

**KRT1**

**Name:** keratin 1

**Aliases:** 67 kDa cytokeratin, CK1, CK-1, Cytokeratin-1, EHK1, Hair alpha protein, K1, Keratin, type II cytoskeletal 1, Keratin-1, KRT1A, KRTA

<http://www.ihop-net.org/UniPub/iHOP/gismo/89718.html>

**KRT5**

**Name:** keratin 5

**Aliases:** 58 kDa cytokeratin, CK5, CK-5, Cytokeratin-5, DDD, EBS2, K5, Keratin, type II cytoskeletal 5, Keratin-5, KRT5A

<http://www.ihop-net.org/UniPub/iHOP/gismo/89722.html>

**KRT13**

**Name:** keratin 13

**Aliases:** CK13, CK-13, Cytokeratin-13, K13, Keratin, type I cytoskeletal 13, Keratin-13, MGC161462, MGC3781

<http://www.ihop-net.org/UniPub/iHOP/gismo/89730.html>

**LAMB2**

**Name:** laminin, beta 2 (laminin S)

**Aliases:** Laminin B1s chain, Laminin subunit beta-2, LAMS, S-laminin

<http://www.ihop-net.org/UniPub/iHOP/gismo/89780.html>

**LCE1C**

**Name:** late cornified envelope 1C

**Aliases:** Late cornified envelope protein 1C, Late envelope protein 3, LEP3

<http://www.ihop-net.org/UniPub/iHOP/mgi/112142.html>

**LEF1**

**Name:** lymphoid enhancer-binding factor 1

**Aliases:** DKFZp586H0919, LEF-1, Lymphoid enhancer-binding factor 1, T cell-specific transcription factor 1-alpha, TCF1ALPHA, TCF1-alpha

<http://www.ihop-net.org/UniPub/iHOP/gismo/99252.html>

**LIG3**

**Name:** ligase III, DNA, ATP-dependent

**Aliases:** DNA ligase 3, DNA ligase III

<http://www.ihop-net.org/UniPub/iHOP/gismo/89841.html>

**LMNA**

**Name:** lamin A/C

**Aliases:** 70 kDa lamin, CDCD1, CDDC, CMD1A, CMT2B1, EMD2, FPL, FPLD, HGPS, IDC, Lamin-A/C, LDP1, LFP, LGMD1B, LMN1, LMNC, PRO1, Renal carcinoma antigen NY-REN-32

<http://www.ihop-net.org/UniPub/iHOP/gismo/89859.html>

**LGALS7**

**Name:** lectin, galactoside-binding, soluble, 7

**Aliases:** GAL7, Gal-7, Galectin-7, HKL-14, LGALS7A, p53-induced gene 1 protein, PI7, PIG1, TP53I1

<http://www.ihop-net.org/UniPub/iHOP/gismo/89827.html>

**LRIG3**

**Name:** leucine-rich repeats and immunoglobulin-like domains 3

**Aliases:** FLJ26573, FLJ90440, KIAA3016, Leucine-rich repeats and immunoglobulin-like domains protein 3, LIG3, LIG-3, UNQ287/PRO326/PRO335

<http://www.ihop-net.org/UniPub/iHOP/gismo/105773.html>

**MAFG**

**Name:** v-maf musculoaponeurotic fibrosarcoma oncogene homolog G (avian)

**Aliases:** hMAF, MGC13090, MGC20149, Transcription factor MafG, V-maf musculoaponeurotic fibrosarcoma oncogene homolog G

<http://www.ihop-net.org/UniPub/iHOP/gismo/89946.html>

**MAP9**

**Name:** microtubule-associated protein 9

**Aliases:** ASAP, Aster-associated protein, FLJ21159, Microtubule-associated protein 9

<http://www.ihop-net.org/UniPub/iHOP/gismo/102924.html>

**MAOA**

**Name:** monoamine oxidase A

**Aliases:** MAO-A, Monoamine oxidase type A

<http://www.ihop-net.org/UniPub/iHOP/gismo/89975.html>

**MCM9**

**Name:** minichromosome maintenance complex component 9

**Aliases:** C6orf61, dJ329L24.1, dJ329L24.3, DNA replication licensing factor MCM9, FLJ13942, FLJ20170, FLJ56845, hMCM9, MCMDC1, MGC35304, Mini-chromosome maintenance deficient 9, Mini-chromosome maintenance deficient domain-containing protein 1

http://www.ihop-net.org/UniPub/iHOP/gismo/109262.html

**MCMDC1**

***See MCM9***

**MCP-1**

***See CCL2***

**MID1**

**Name:** midline 1 (Opitz/BBB syndrome)

**Aliases:** BBBG1, FXY, GBBB1, Midin, MIDIN, Midline-1, Midline 1 RING finger protein, OGS1, OS, OSX, RING finger protein 59, RNF59, TRIM18, Tripartite motif-containing protein 18, XPRF, ZNFXY

<http://www.ihop-net.org/UniPub/iHOP/gismo/90110.html>

**MALAT1**

**Name:** metastasis associated lung adenocarcinoma transcript 1 (non-protein coding)

**Aliases:** HCN, MALAT-1, Metastasis-associated lung adenocarcinoma transcript 1, NCRNA00047, NEAT2, PRO1073, PRO2853

<http://www.ihop-net.org/UniPub/iHOP/gismo/112617.html>

**MKLP-1**

**Name:** kinesin family member 23

**Aliases:** CHO1, Kinesin-like protein 5, Kinesin-like protein KIF23, KNSL5, Mitotic kinesin-like protein 1, MKLP1, MKLP-1

<http://www.ihop-net.org/UniPub/iHOP/gismo/94699.html>

**MST1**

**Name:** macrophage stimulating 1 (hepatocyte growth factor-like)

**Aliases:** D3F15S2, DNF15S2, Hepatocyte growth factor-like protein, HGFL, Macrophage-stimulating protein, Macrophage stimulatory protein, MSP, NF15S2

<http://www.ihop-net.org/UniPub/iHOP/gismo/90301.html>

**MST2**

***See STK3***

**MYC**

**Name:** v-myc myelocytomatosis viral oncogene homolog (avian)

**Aliases:** bHLHe39, c-Myc, MRTL, Myc proto-oncogene protein, Transcription factor p64

<http://www.ihop-net.org/UniPub/iHOP/gismo/90405.html>

**NANOG**

**Name:** Nanog homeobox

**Aliases:** FLJ12581, FLJ40451, hNanog, Homeobox protein NANOG, Homeobox transcription factor Nanog

<http://www.ihop-net.org/UniPub/iHOP/gismo/102955.html>

**NEK6**

**Name:** NIMA (never in mitosis gene a)-related kinase 6

**Aliases:** NimA-related protein kinase 6, Protein kinase SID6-1512, Serine/threonine-protein kinase Nek6, SID6-1512

<http://www.ihop-net.org/UniPub/iHOP/gismo/95835.html>

**NF-kappaB**

***See NFKB1 and NFKB2***

**NCAPD3**

**Name:** non-SMC condensin II complex, subunit D3

**Aliases:** CAPD3, CAP-D3, Condensin-2 complex subunit D3, FLJ42888, hCAP-D3, hcp-6, hHCP-6, KIAA0056, MGC104671, Non-SMC condensin II complex subunit D3

<http://www.ihop-net.org/UniPub/iHOP/gismo/96745.html>

**NCAPG2**

**Name:** non-SMC condensin II complex, subunit G2

**Aliases:** CAP-G2, Chromosome-associated protein G2, Condensin-2 complex subunit G2, FLJ20311, hCAP-G2, Leucine zipper protein 5, LUZP5, MTB, Non-SMC condensin II complex subunit G2

<http://www.ihop-net.org/UniPub/iHOP/gismo/100145.html>

**NFATC1**

**Name:** nuclear factor of activated T-cells, cytoplasmic, calcineurin-dependent 1

**Aliases:** MGC138448, NFAT2, NFATc, NFATC, NF-ATc, NF-ATC, NF-ATc1, NFAT transcription complex cytosolic component, Nuclear factor of activated T-cells, cytoplasmic 1

<http://www.ihop-net.org/UniPub/iHOP/gismo/90556.html>

**NFKB1**

**Name:** nuclear factor of kappa light polypeptide gene enhancer in B-cells 1

**Aliases:** DKFZp686C01211, DNA-binding factor KBF1, EBP-1, KBF1, MGC54151, NF-kappa-B, NF-kappabeta, NFKB-p105, NFKB-p50, Nuclear factor NF-kappa-B p105 subunit, p105, p50

<http://www.ihop-net.org/UniPub/iHOP/gismo/90569.html>

**NFKB2**

**Name:** nuclear factor of kappa light polypeptide gene enhancer in B-cells 2 (p49/p100)

**Aliases:** DNA-binding factor KBF2, H2TF1, Lymphocyte translocation chromosome 10, Lyt10, LYT10, LYT-10, Nuclear factor NF-kappa-B p100 subunit, Oncogene Lyt-10

<http://www.ihop-net.org/UniPub/iHOP/gismo/90570.html>

**NKAP**

**Name:** NFKB activating protein

**Aliases:** FLJ22626, NF-kappa-B-activating protein

<http://www.ihop-net.org/UniPub/iHOP/gismo/102677.html>

**NKRF**

**Name:** NFKB repressing factor

**Aliases:** ITBA4, ITBA4 protein, NF-kappa-B-repressing factor, NFkB-repressing factor, NRF, Transcription factor NRF

<http://www.ihop-net.org/UniPub/iHOP/gismo/100913.html>

**NRP2**

**Name:** neuropilin 2

**Aliases:** MGC126574, Neuropilin-2, NP2, NPN2, PRO2714, Vascular endothelial cell growth factor 165 receptor 2, VEGF165R2

<http://www.ihop-net.org/UniPub/iHOP/gismo/94180.html>

**NUDC**

**Name:** nuclear distribution gene C homolog (A. nidulans)

**Aliases:** HNUDC, MNUDC, NPD011, Nuclear distribution protein C homolog, Nuclear migration protein nudC, NudC

<http://www.ihop-net.org/UniPub/iHOP/gismo/95785.html>

**OCT4**

***See POU5F1*.**

**P21**

***See CDKN1A***

**P4HB**

**Name:** prolyl 4-hydroxylase, beta polypeptide

**Aliases:** Cellular thyroid hormone-binding protein, DSI, ERBA2L, GIT, P4Hbeta, p55, PDI, PDIA1, PHDB, PO4DB, PO4HB, PROHB, Prolyl 4-hydroxylase subunit beta, Protein disulfide-isomerase

<http://www.ihop-net.org/UniPub/iHOP/gismo/90793.html>

**Paxillin**

***See PXN***

**PCDH21**

**Name:** protocadherin 21

**Aliases:** DKFZp434A132, KIAA1775, Photoreceptor cadherin, prCAD, PRCAD, Protocadherin-21

<http://www.ihop-net.org/UniPub/iHOP/gismo/104958.html>

**PDK2**

**Name:** pyruvate dehydrogenase kinase, isozyme 2

**Aliases:** PDHK2, Pyruvate dehydrogenase kinase isoform 2

<http://www.ihop-net.org/UniPub/iHOP/gismo/90914.html>

**PDXP**

**Name:** pyridoxal (pyridoxine, vitamin B6) phosphatase

**Aliases:** CIN, dJ37E16.5, FLJ32703, PLP, PLPP, PLP phosphatase, Pyridoxal phosphate phosphatase

<http://www.ihop-net.org/UniPub/iHOP/gismo/101246.html>

**PIM1**

**Name:** pim-1 oncogene

**Aliases:** Proto-oncogene serine/threonine-protein kinase Pim-1

<http://www.ihop-net.org/UniPub/iHOP/gismo/91035.html>

**PISD**

**Name:** phosphatidylserine decarboxylase

**Aliases:** DJ858B16, dJ858B16.2, DKFZp566G2246, Phosphatidylserine decarboxylase proenzyme, PSD, PSDC, PSSC

<http://www.ihop-net.org/UniPub/iHOP/gismo/97110.html>

**PLK1**

**Name:** polo-like kinase 1 (Drosophila)

**Aliases:** PLK, PLK-1, Polo-like kinase 1, Serine/threonine-protein kinase 13, Serine/threonine-protein kinase PLK1, STPK13

<http://www.ihop-net.org/UniPub/iHOP/gismo/91088.html>

**PKP1**

**Name:** plakophilin 1 (ectodermal dysplasia/skin fragility syndrome)

**Aliases:** B6P, Band-6 protein, MGC138829, Plakophilin-1

<http://www.ihop-net.org/UniPub/iHOP/gismo/91059.html>

**POU5F1**

**Name:** POU class 5 homeobox 1

**Aliases:** MGC22487, OCT3, Oct-3, Oct4, OCT4, Oct-4, Octamer-binding transcription factor 3, OTF3, OTF4, POU domain, class 5, transcription factor 1

<http://www.ihop-net.org/UniPub/iHOP/gismo/91197.html>

**pRB**

***See RB1***

**PRNP**

**Name:** prion protein

**Aliases:** ASCR, CD230, CJD, GSS, Major prion protein, MGC26679, prion, PRIP, PrP, PRP, PrP27-30, PrP33-35C, PrPc

<http://www.ihop-net.org/UniPub/iHOP/gismo/91350.html>

**PRR16**

**Name:** proline rich 16

**Aliases:** DSC54, Mesenchymal stem cell protein DSC54, MGC104614, Proline-rich protein 16

<http://www.ihop-net.org/UniPub/iHOP/gismo/99379.html>

**PSA**

***See KLK3***

**PSEN2**

**Name:** presenilin 2 (Alzheimer disease 4)

**Aliases:** AD3L, AD3LP, AD4, AD5, E5-1, Presenilin-2, PS2, PS-2, PSNL2, STM2, STM-2

<http://www.ihop-net.org/UniPub/iHOP/gismo/91387.html>

**PSF**

***See SFPQ***

**PTEN**

**Name:** phosphatase and tensin homolog

**Aliases:** 10q23del, BZS, MGC11227, MHAM, MMAC1, Mutated in multiple advanced cancers 1, Phosphatase and tensin homolog, Phosphatidylinositol-3,4,5-trisphosphate 3-phosphatase and dual-specificity protein phosphatase PTEN, PTEN1, TEP1

<http://www.ihop-net.org/UniPub/iHOP/gismo/91451.html>

**PTGER3**

**Name:** prostaglandin E receptor 3 (subtype EP3)

**Aliases:** EP3, EP3e, EP3-I, EP3-II, EP3-III, EP3-IV, MGC141828, MGC141829, MGC27302, PGE2-R, PGE receptor, EP3 subtype, Prostaglandin E2 receptor EP3 subtype, Prostanoid EP3 receptor

<http://www.ihop-net.org/UniPub/iHOP/gismo/91456.html>

**PTTG1**

**Name:** pituitary tumor-transforming 1

**Aliases:** EAP1, Esp1-associated protein, hPTTG, HPTTG, MGC126883, MGC138276, Pituitary tumor-transforming gene 1 protein, PTTG, securin, Securin, Tumor-transforming protein 1, TUTR1

<http://www.ihop-net.org/UniPub/iHOP/gismo/94488.html>

**PXN**

**Name:** paxillin

**Aliases:** Paxillin

<http://www.ihop-net.org/UniPub/iHOP/gismo/91546.html>

**RAF1**

**Name:** v-raf-1 murine leukemia viral oncogene homolog 1

**Aliases:** cRaf, CRAF, c-Raf, C-RAF, NS5, RAF, Raf-1, RAF proto-oncogene serine/threonine-protein kinase

<http://www.ihop-net.org/UniPub/iHOP/gismo/91610.html>

**RAP1B**

**Name:** RAP1B, member of RAS oncogene family

**Aliases:** DKFZp586H0723, GTP-binding protein smg p21B, K-REV, OK/SW-cl.11, RAL1B, Ras-related protein Rap-1b

<http://www.ihop-net.org/UniPub/iHOP/gismo/91622.html>

**RASSF1**

**Name:** Ras association (RalGDS/AF-6) domain family member 1

**Aliases:** 123F2, NORE2A, Ras association domain-containing protein 1, RASSF1A, RDA32, REH3P21

<http://www.ihop-net.org/UniPub/iHOP/gismo/96194.html>

**RB1**

**Name:** retinoblastoma 1

**Aliases:** OSRC, p105-Rb, pp110, pRb, Rb, RB, Retinoblastoma-associated protein

<http://www.ihop-net.org/UniPub/iHOP/gismo/91639.html>

**RN5S2@**

**Name:** RNA, 5S cluster 2

**Aliases:** N/A

<http://www.ihop-net.org/UniPub/iHOP/mgi/91735.html>

**RNF40**

**Name:** ring finger protein 40

**Aliases:** 95 kDa retinoblastoma-associated protein, BRE1B, BRE1-B, DKFZp686K191, E3 ubiquitin-protein ligase BRE1B, KIAA0661, MGC13051, RBP95, RING finger protein 40, STARING

<http://www.ihop-net.org/UniPub/iHOP/gismo/94978.html>

**SARA1**

**Name:** SAR1 homolog A (S. cerevisiae)

**Aliases:** COPII-associated small GTPase, GTP-binding protein SAR1a, masra2, SAR1, Sara, SARA, SARA1

<http://www.ihop-net.org/UniPub/iHOP/gismo/101098.html>

**SEC61A1**

**Name:** Sec61 alpha 1 subunit (S. cerevisiae)

**Aliases:** HSEC61, Protein transport protein Sec61 subunit alpha isoform 1, SEC61, SEC61A, Sec61 alpha-1

<http://www.ihop-net.org/UniPub/iHOP/gismo/98855.html>

**SEMA6A**

**Name:** sema domain, transmembrane domain (TM), and cytoplasmic domain, (semaphorin) 6A

**Aliases:** HT018, KIAA1368, SEMA, SEMA6A1, SEMA6A-1, Semaphorin-6A, Semaphorin-6A-1, Semaphorin VIA, SEMAQ, Sema VIA, VIA

<http://www.ihop-net.org/UniPub/iHOP/gismo/101532.html>

**SFPQ**

**Name:** splicing factor proline/glutamine-rich (polypyrimidine tract binding protein associated)

**Aliases:** 100 kDa DNA-pairing protein, DNA-binding p52/p100 complex, 100 kDa subunit, hPOMp100, Polypyrimidine tract-binding protein-associated-splicing factor, POMP100, PSF, PTB-associated-splicing factor, Splicing factor, proline-and glutamine-rich

<http://www.ihop-net.org/UniPub/iHOP/gismo/92104.html>

**SGTA**

**Name:** small glutamine-rich tetratricopeptide repeat (TPR)-containing, alpha

**Aliases:** alphaSGT, Alpha-SGT, hSGT, SGT, SGT1, Small glutamine-rich tetratricopeptide repeat-containing protein alpha, UBP, Vpu-binding protein

<http://www.ihop-net.org/UniPub/iHOP/gismo/92132.html>

**SHC1**

**Name:** SHC (Src homology 2 domain containing) transforming protein 1

**Aliases:** FLJ26504, p66, SH2 domain protein C1, SHC, SHCA, SHC-transforming protein 1, Src homology 2 domain-containing-transforming protein C1

<http://www.ihop-net.org/UniPub/iHOP/gismo/92146.html>

**SHC4**

**Name:** SHC (Src homology 2 domain containing) family, member 4

**Aliases:** hShcD, MGC34023, Rai-like protein, RaLP, SH2 domain protein C4, SHCD, SHC-transforming protein 4, Src homology 2 domain-containing-transforming protein C4, UNQ6438/PRO21364

<http://www.ihop-net.org/UniPub/iHOP/gismo/116410.html>

**SLC12A2**

**Name:** solute carrier family 12 (sodium/potassium/chloride transporters), member 2

**Aliases:** Basolateral Na-K-Cl symporter, BSC, BSC2, Bumetanide-sensitive sodium-(potassium)-chloride cotransporter 1, MGC104233, NKCC1, Solute carrier family 12 member 2

<http://www.ihop-net.org/UniPub/iHOP/gismo/92234.html>

**SLAMF1**

**Name:** signaling lymphocytic activation molecule family member 1

**Aliases:** CD150, CDw150, IPO-3, Signaling lymphocytic activation molecule, SLAM

<http://www.ihop-net.org/UniPub/iHOP/gismo/92182.html>

**SLIT3**

**Name:** slit homolog 3 (Drosophila)

**Aliases:** FLJ10764, KIAA0814, MEGF5, Multiple epidermal growth factor-like domains 5, SLIL2, SLIT1, slit2, Slit-3, Slit homolog 3 protein, UNQ691/PRO1336

<http://www.ihop-net.org/UniPub/iHOP/gismo/92260.html>

**SNORA60**

**Name:** small nucleolar RNA, H/ACA box 60

**Aliases:** ACA60

<http://www.ihop-net.org/UniPub/iHOP/mgi/1490287.html>

**SNHG1**

**Name:** small nucleolar RNA host gene 1 (non-protein coding)

**Aliases:** NCRNA00057, U22HG, UHG

<http://www.ihop-net.org/UniPub/iHOP/mgi/2075735.html>

**SNHG8**

**Name:** small nucleolar RNA host gene 8 (non-protein coding)

**Aliases:** N/A

<http://www.ihop-net.org/UniPub/iHOP/mgi/3092347.html>

**SOX2**

**Name:** SRY (sex determining region Y)-box 2

**Aliases:** ANOP3, MCOPS3, MGC2413, Transcription factor SOX-2

<http://www.ihop-net.org/UniPub/iHOP/gismo/92327.html>

**SOX9**

**Name:** SRY (sex determining region Y)-box 9

**Aliases:** CMD1, CMPD1, SRA1, Transcription factor SOX-9

<http://www.ihop-net.org/UniPub/iHOP/gismo/92332.html>

**SPP1**

**Name:** secreted phosphoprotein 1

**Aliases:** BNSP, Bone sialoprotein 1, BSPI, ETA-1, MGC110940, Nephropontin, OPN, Osteopontin, PSEC0156, Secreted phosphoprotein 1, SPP-1, Urinary stone protein, Uropontin

**SRD5A2**

**Name:** steroid-5-alpha-reductase, alpha polypeptide 2 (3-oxo-5 alpha-steroid delta 4-dehydrogenase alpha 2)

**Aliases:** 4-dehydrogenase alpha 2)   3-oxo-5-alpha-steroid 4-dehydrogenase 2, 5 alpha-SR2, SR type 2, Steroid 5-alpha-reductase 2, Type II 5-alpha reductase

<http://www.ihop-net.org/UniPub/iHOP/gismo/92381.html>

**STAT3**

**Name:** signal transducer and activator of transcription 3 (acute-phase response factor)

**Aliases:** Acute-phase response factor, APRF, FLJ20882, HIES, MGC16063, Signal transducer and activator of transcription 3

<http://www.ihop-net.org/UniPub/iHOP/gismo/92438.html>

**STAT6**

**Name:** signal transducer and activator of transcription 6, interleukin-4 induced

**Aliases:** D12S1644, IL-4 Stat, IL-4-STAT, Signal transducer and activator of transcription 6, STAT6B, STAT6C

<http://www.ihop-net.org/UniPub/iHOP/gismo/92442.html>

**STEAP**

***See STEAP1, STEAP2, STEAP3 and STEAP4***

**STEAP1**

**Name:** six transmembrane epithelial antigen of the prostate 1

**Aliases:** Metalloreductase STEAP1, MGC19484, PRSS24, Six-transmembrane epithelial antigen of prostate 1, STEAP

<http://www.ihop-net.org/UniPub/iHOP/gismo/97804.html>

**STEAP2**

**Name:** six transmembrane epithelial antigen of the prostate 2

**Aliases:** IPCA1, IPCA-1, Metalloreductase STEAP2, PCANAP1, Prostate cancer-associated protein 1, Protein upregulated in metastatic prostate cancer, PUMPCn, Six-transmembrane epithelial antigen of prostate 2, SixTransMembrane protein of prostate 1, STAMP1, STMP, UNQ6507/PRO23203

<http://www.ihop-net.org/UniPub/iHOP/gismo/109532.html>

**STEAP3**

**Name:** STEAP family member 3

**Aliases:** dudlin-2, Dudulin-2, hpHyde, hTSAP6, Metalloreductase STEAP3, pHyde, Six-transmembrane epithelial antigen of prostate 3, STMP3, TSAP6, Tumor suppressor-activated pathway protein 6

<http://www.ihop-net.org/UniPub/iHOP/gismo/100441.html>

**STEAP4**

**Name:** STEAP family member 4

**Aliases:** DKFZp666D049, FLJ23153, Metalloreductase STEAP4, Six-transmembrane epithelial antigen of prostate 4, SixTransMembrane protein of prostate 2, STAMP2, TIARP, TNFAIP9, Tumor necrosis factor, alpha-induced protein 9

<http://www.ihop-net.org/UniPub/iHOP/gismo/102764.html>

**STK3**

**Name:** serine/threonine kinase 3 (STE20 homolog, yeast)

**Aliases:** FLJ90748, KRS1, Mammalian STE20-like protein kinase 2, MST2, MST-2, Serine/threonine-protein kinase 3, Serine/threonine-protein kinase Krs-1, STE20-like kinase MST2

<http://www.ihop-net.org/UniPub/iHOP/gismo/92451.html>

**STK4**

**Name:** serine/threonine kinase 4

**Aliases:** DKFZp686A2068, KRS2, Mammalian STE20-like protein kinase 1, MST1, MST-1, Serine/threonine-protein kinase 4, Serine/threonine-protein kinase Krs-2, STE20-like kinase MST1, YSK3

<http://www.ihop-net.org/UniPub/iHOP/gismo/92452.html>

**STK6**

***See AURKA***

**TACSTD2**

**Name:** tumor-associated calcium signal transducer 2

**Aliases:** Cell surface glycoprotein Trop-2, EGP-1, GA733, GA733-1, M1S1, Pancreatic carcinoma marker protein GA733-1, TROP2, Tumor-associated calcium signal transducer 2

<http://www.ihop-net.org/UniPub/iHOP/gismo/89921.html>

**TBX1**

**Name:** T-box 1

**Aliases:** CAFS, CTHM, DGCR, DGS, DORV, T-box protein 1, T-box transcription factor TBX1, TBX1C, Testis-specific T-box protein, TGA, VCFS

<http://www.ihop-net.org/UniPub/iHOP/gismo/92549.html>

**TERF2**

**Name:** telomeric repeat binding factor 2

**Aliases:** Telomeric DNA-binding protein, Telomeric repeat-binding factor 2, TRBF2, TRF2, TTAGGG repeat-binding factor 2

<http://www.ihop-net.org/UniPub/iHOP/gismo/92660.html>

**TERF2IP**

**Name:** telomeric repeat binding factor 2, interacting protein

**Aliases:** DRIP5, hRap1, PP8000, RAP1, Telomeric repeat-binding factor 2-interacting protein 1, TRF2-interacting telomeric protein Rap1

<http://www.ihop-net.org/UniPub/iHOP/gismo/99848.html>

**TERT**

**Name:** telomerase reverse transcriptase

**Aliases:** EST2, hEST2, HEST2, TCS1, Telomerase-associated protein 2, Telomerase catalytic subunit, Telomerase reverse transcriptase, TP2, TRT

<http://www.ihop-net.org/UniPub/iHOP/gismo/92661.html>

**TFDP1**

**Name:** transcription factor Dp-1

**Aliases:** DP1, Dp-1, DRTF1, DRTF1-polypeptide 1, E2F dimerization partner 1, Transcription factor Dp-1

<http://www.ihop-net.org/UniPub/iHOP/gismo/92672.html>

**TGM3**

**Name:** transglutaminase 3 (E polypeptide, protein-glutamine-gamma-glutamyltransferase)

**Aliases:** MGC126249, MGC126250, Protein-glutamine gamma-glutamyltransferase E, TG(E), TGase E, TGE, Transglutaminase-3

<http://www.ihop-net.org/UniPub/iHOP/gismo/92698.html>

**THBS3**

**Name:** thrombospondin 3

**Aliases:** MGC119564, MGC119565, Thrombospondin-3, TSP3

<http://www.ihop-net.org/UniPub/iHOP/gismo/92704.html>

**TMED3**

**Name:** transmembrane emp24 protein transport domain containing 3

**Aliases:** C15orf22, Membrane protein p24B, MGC133022, p24B, P24B, Transmembrane emp24 domain-containing protein 3, UNQ5357/PRO1078

<http://www.ihop-net.org/UniPub/iHOP/gismo/96836.html>

**TNF**

**Name:** tumor necrosis factor (TNF superfamily, member 2)

**Aliases:** Cachectin, DIF, TNFA, TNF-a, TNF-alpha, TNFSF2, Tumor necrosis factor, Tumor necrosis factor ligand superfamily member 2

<http://www.ihop-net.org/UniPub/iHOP/gismo/92766.html>

**TNFRSF5**

***See CD40***

**TP53**

**Name:** tumor protein p53

**Aliases:** Antigen NY-CO-13, Cellular tumor antigen p53, FLJ92943, LFS1, p53, P53, Phosphoprotein p53, TRP53, Tumor suppressor p53

<http://www.ihop-net.org/UniPub/iHOP/gismo/92798.html>

**TP63**

**Name:** tumor protein p63

**Aliases:** AIS, B(p51A), B(p51B), Chronic ulcerative stomatitis protein, CUSP, EEC3, Keratinocyte transcription factor KET, KET, LMS, NBP, OFC8, p40, p51, p53CP, p63, P63, p73H, P73H, p73L, P73L, RHS, SHFM4, TP53CP, TP53L, TP73L, Transformation-related protein 63, Tumor protein 63, Tumor protein p73-like

<http://www.ihop-net.org/UniPub/iHOP/gismo/94006.html>

**TRF2**

***See TERF2***

**VPS39**

**Name:** vacuolar protein sorting 39 homolog (S. cerevisiae)

**Aliases:** FLJ21681, FLJ46546, hVam6p, KIAA0770, TLP, VAM6, Vam6/Vps39-like protein

<http://www.ihop-net.org/UniPub/iHOP/gismo/96768.html>

**WARS**

**Name:** tryptophanyl-tRNA synthetase

**Aliases:** GAMMA-2, hWRS, IFI53, IFP53, Interferon-induced protein 53, TrpRS, Tryptophan--tRNA ligase, Tryptophanyl-tRNA synthetase, cytoplasmic, WRS

<http://www.ihop-net.org/UniPub/iHOP/gismo/93081.html>

**WDFY3**

**Name:** WD repeat and FYVE domain containing 3

**Aliases:** Alfy, ALFY, Autophagy-linked FYVE protein, KIAA0993, MGC16461, WD repeat and FYVE domain-containing protein 3, ZFYVE25

<http://www.ihop-net.org/UniPub/iHOP/gismo/96487.html>

**XBP1**

**Name:** X-box binding protein 1

**Aliases:** Tax-responsive element-binding protein 5, TREB5, X-box-binding protein 1, XBP-1, XBP2

<http://www.ihop-net.org/UniPub/iHOP/gismo/93121.html>

**XPA**

**Name:** xeroderma pigmentosum, complementation group A

**Aliases:** DNA repair protein complementing XP-A cells, Xeroderma pigmentosum group A-complementing protein, XP1, XPAC

<http://www.ihop-net.org/UniPub/iHOP/gismo/93134.html>

**ZAK**

**Name:** sterile alpha motif and leucine zipper containing kinase AZK

**Aliases:** AZK, Cervical cancer suppressor gene 4 protein, HCCS4, HCCS-4, Leucine zipper-and sterile alpha motif-containing kinase, Mitogen-activated protein kinase kinase kinase MLT, Mixed lineage kinase-related kinase, MLK7, mlklak, MLK-like mitogen-activated protein triple kinase, MLK-related kinase, MLT, MLTK, MRK, Sterile alpha motif-and leucine zipper-containing kinase AZK

<http://www.ihop-net.org/UniPub/iHOP/gismo/99623.html>
